# Contrasting Patterns in Ambient PM_2.5_ Exposure Disparity Across Population Subgroups in Urban and Rural India

**DOI:** 10.1029/2025GH001387

**Published:** 2026-01-24

**Authors:** Debajit Sarkar, Alok Kumar, Fahad Imam, Santu Ghosh, Julian D. Marshall, Joshua Apte, Luke D. Knibs, Pallavi Pant, Yang Liu, Sagnik Dey

**Affiliations:** ^1^ Centre for Atmospheric Sciences Indian Institute of Technology Delhi New Delhi India; ^2^ St. John's Medical College Bangalore India; ^3^ Department of Civil and Environmental Engineering University of Washington Seattle WA USA; ^4^ Department of Civil and Engineering University of California Berkeley Berkeley CA USA; ^5^ School of Public Health University of Sydney Camperdown NSW Australia; ^6^ Health Effects Institute Boston MA USA; ^7^ Rollins School of Public Health Emory University Atlanta GA USA; ^8^ Adjunct Faculty Korea University Seoul South Korea; ^9^ School of Public Policy Indian Institute of Technology Delhi New Delhi India

**Keywords:** ambient PM_2.5_, exposure, disparity, demographic subgroup, India

## Abstract

Ambient PM_2.5_ exposure poses the greatest environmental risk to public health in India. While several studies have quantified the changing patterns of exposure, the extent of inequality in exposure among population subgroups at the sub‐national scale remains unknown. In this study, we examined the disparity in ambient PM_2.5_ exposure across various population subgroups in urban and rural India and analyzed its changes in recent years by integrating satellite‐derived PM_2.5_ concentrations (1‐km × 1‐km) with sociodemographic information from the 4th (2015–2016) and 5th (2019–2021) rounds of the National Family Health Survey. We found a larger absolute disparity (60–90 µgm^−3^) in high socio‐demographic index (SDI) states compared to middle and lower SDI states. Moreover, we discovered that ambient PM_2.5_ exposure was higher (indicated by relative disparities in terms of *Z*_score_) among the top and bottom quantiles of wealth index and the other backward caste subgroup (*Z*_score_ > ±0.02, *p* < 0.1) than among their demographic counterparts in middle and high SDI states. From 2015–2016 to 2019–2021, the disparity in ambient PM_2.5_ exposure across subgroups increased in urban areas, while it either remained static or decreased in rural areas. India's urban‐centric approach to addressing air pollution may further exacerbate disparities among diverse demographics. Therefore, we recommend the formulation of targeted policies aimed at reducing ambient PM_2.5_ exposure and alleviating disparities by prioritizing actions for the vulnerable subgroups.

## Plain Language Summary

Long‐term exposure to ambient fine particulate matter (PM_2.5_) poses a significant health burden for the Indian population. Studies from the United States and China have reported an unequal distribution of ambient PM_2.5_ among population subgroups. Whether this applies to a diverse country like India remains uncertain. We integrated satellite‐derived ambient PM_2.5_ concentrations with socio‐demographic data from a nationwide survey to first estimate the weighted mean PM_2.5_ exposure at the sub‐national scale for various population subgroups. We then identified vulnerable subgroups classified by gender, caste, and wealth index. Our findings indicate that exposure variation is greater among both wealthier and poorer subgroups, with increasing disparities in urban areas compared to rural areas in recent years. These results underscore the need for tailored mitigation actions for vulnerable subgroups facing higher air pollution levels in regions with considerable inequality.

## Key Points

- Demographic subgroups in India were disproportionately exposed to varying levels of ambient PM_2.5_ exposure
- Urban‐rural heterogeneity was larger among the high SDI states and subgroups of top and bottom quantiles of wealth‐Index breathed more polluted air than other subgroups
- Inequality in ambient PM_2.5_ exposure across the population subgroups increased in urban regions but remained static in rural regions

## 1 Introduction

Air pollution is a leading environmental risk to public health around the globe (Balakrishnan et al., 2019). Though air pollution levels have declined in developed countries, many low and middle‐income countries (LMICs), including India, continue to experience high pollution levels (de Bont et al., 2024). Depending on the spatial distribution of demographics, exposure to ambient PM_2.5_ (fine particles with an aerodynamic diameter less than 2.5 μm) among various population subgroups may vary, leading to disparities that inhibit progress toward achieving various Sustainable Development Goals (SDGs) (Rafaj et al., 2018). A significant body of environmental justice (EJ) research from the United States and elsewhere has reported heterogeneous distributions of ambient PM_2.5_ across population subgroups characterized by race‐ethnicity, sociodemographic classes, location (i.e., urban and rural regions), and time points (Bullock et al., 2018; Clark et al., 2017; Rosofsky et al., 2018; Salazar et al., 2019). The general consensus is that ambient air pollution burdens socially disadvantaged and marginalized subgroups more than their wealthier counterparts (Clark et al., 2022; Di, Wang, et al., 2017; Di, Dai, et al., 2017; Kerr et al., 2024; Wang et al., 2017). However, studies from China have reported a reverse pattern, indicating that subgroups with higher socioeconomic status experience elevated levels of air pollution. Such findings have prompted efforts to consider inequities when implementing clean air policies in these regions (deSouza et al., 2023).

India, the most populous country, has one of the highest levels of air pollution globally (Katoch et al., 2023). Chronic exposure to ambient PM_2.5_ was responsible for 0.95 million (95% confidence intervals: 0.62–1.26) premature deaths and 27.4 million (17.7–36.3) disability‐adjusted life years (DALYs) in India in 2021 (Murray et al., 2024). Several recent studies reported spatial patterns of population‐weighted PM_2.5_ exposure, its temporal variation, and the associated health effects, including state‐level estimates of mortality and DALY burden under the Global Burden of Disease India (GBD‐India) study (Chowdhury et al., 2020; Dey et al., 2020; Murray et al., 2020; Pandey et al., 2021). However, the disparity in ambient PM_2.5_ exposure across population subgroups has remained mostly unexplored in those studies.

Existing studies in India exploring social justice primarily focused on societal access (e.g., education, drinking water, healthcare access) through descriptive statistics rather than air pollution exposure (Barik & Thorat, 2015; Garg et al., 2022; Rani, 2022). Only recently has ambient PM_2.5_ exposure attributable to coal‐fired power plants been found to be higher among poorer and lower‐caste subgroups in the eastern states of India (Kopas et al., 2020). Air pollution‐related mortality has been shown to be higher than average for indigent sub‐populations across the central and southern peninsular states (Sengupta et al., 2022). Other than these two studies and the one by deSouza et al. (2023), where variations in exposure across demographic classes were examined. Due to their submicron size, PM_2.5_ particles can undergo long‐range atmospheric transport from emission sources (Rafaj et al., 2018; Rosofsky et al., 2018). In geographically expansive states, even the individuals belonging to the same demographic subgroup may experience varying levels of ambient PM_2.5_ concentration depending on their proximity to emission sources and other contextual socioeconomic factors (Kerr et al., 2024). To our knowledge, no study has systematically quantified the maximum intra‐state variation in PM_2.5_ concentration or the “*absolute disparity*,” particularly in India, where states vary widely in size and geographic characteristics. More importantly, there is a lack of comprehensive assessment of the “*relative disparity*” in ambient PM_2.5_ exposure across demographic subgroups within India. As a socially diverse country, India exhibits substantial variability in socioeconomic conditions, emission source‐types, and local‐scale meteorology and land cover across its urban and rural regions (Dey et al., 2020; Katoch et al., 2023). These differences can result in substantial spatial heterogeneity in ambient PM_2.5_ concentrations and associated exposures among sub‐populations. Therefore, assessing urban‐rural disparities in ambient PM_2.5_ across states and demographic subgroups is critical for informing targeted, equity‐focused policy interventions aimed at achieving environmental justice in the near future. Moreover, spatiotemporal variations in air pollution levels and demographics can alter the disparity in ambient PM_2.5_ across sub‐populations over time (Clark et al., 2017; Colmer et al., 2020; Rosofsky et al., 2018). To our knowledge, this aspect has yet to be comprehensively addressed in the Indian context, which has constrained the implementation of effective targeted clean air policies and action plans to mitigate regional heterogeneity in ambient PM_2.5_ exposure and associated health burdens.

Here, we addressed these crucial issues and examined the disparities in intra‐state variations in ambient PM_2.5_ concentration (absolute disparity) and its exposure among urban and rural population subgroups categorized by gender, wealth‐index, and caste across the states with varying socio‐demographic index (SDI) levels. Based on the relative disparity analysis (denoted by *Z*_score_; see *Methods*), we further identified the vulnerable subgroups within each demographic category.

## 2 Methods

For the exposure disparity assessment, we utilized individual‐level demographic data from the nationally‐representative 4th (2015–2016) and 5th (2019–2021) rounds of the National Family Health Survey (NFHS) (*DHS‐India*) across 30 states and union territories (UTs) (Figures S1 and S2 in Supporting Information S1), along with satellite‐derived annual average ambient PM_2.5_ concentrations (Katoch et al., 2023) (at a 1‐km × 1‐km spatial resolution) for these corresponding time periods. Geo‐spatial buffers (2 km for urban and 5 km for rural regions, respectively) were created around NFHS clusters, which are embedded with geocodes, to first extract localized long‐term PM_2.5_ estimates. These PM_2.5_‐estimates were then integrated with cluster‐level demographic data to assign individual‐level ambient PM_2.5_ exposure. Using this merged data set, we computed empirical statistics to assess ambient PM_2.5_ exposure across population subgroups in both urban and rural settings and examined temporal changes between the two NFHS rounds. We first provide a detailed description of the data sets, followed by the methodology used to estimate absolute and relative disparities in exposure across demographic subgroups.

### 2.1 National Family Health Survey

#### 2.1.1 Sampling Framework of NFHS

The NFHS is a nationally representative household survey that measures population demographics, household characteristics, health, and nutrition. It employs a uniform sample design, ensuring representation at national, state/UT, and district levels, with each survey cluster categorized into urban and rural areas based on assessments of per‐capita income, possession of societal assets, average educational level, and overall socioeconomic status of various demographic subgroups. Each rural stratum within the districts is further sub‐stratified into smaller substrata, taking into account the village population and the proportions of scheduled caste (SC) and scheduled tribe (ST) subgroups.

The 2011 census served as the sampling frame for selecting Primary Sampling Units (PSUs), or clusters. Within each defined rural sampling stratum, a sample of villages was chosen as PSUs or survey clusters; prior to the PSU selection, the clusters were arranged according to the literacy rates of women over 6 years old. Within each urban sampling stratum, a sample of Census Enumeration Blocks was selected as PSUs. Before selection, these clusters were arranged based on the proportion of SC and ST subgroups. In a later stage of selection, a fixed number of 22 households were randomly chosen within each cluster using equal probability systematic selection from a newly created list of households in the selected PSUs. This list was generated from the mapping and household listing operation conducted in each selected PSU before household selection in the second stage. Selected PSUs with an estimated number of at least 300 households were divided into segments of approximately 100–150 households. In the second stage, in every selected urban or rural cluster, 22 households were randomly chosen using systematic sampling. Further details on the sampling framework employed by the NFHS can be found in the publicly‐accessible NFHS reports (*DHS‐India*).

The NFHS is conducted over an extended period, typically spanning 12–24 months. For instance, NFHS‐4 was conducted between January 2015 and December 2016, and NFHS‐5 between June 2019 and April 2021. Data collection does not occur uniformly across all regions; rather, it is staggered over time and varies by state and union territory based on logistical and administrative planning. Each survey cluster is associated with a specific month and year of enumeration, which is recorded in the data set (*DHS‐India*).

Detailed descriptions of the sampling design, weight computation, and estimation of standard error are included in the national report of the NFHSs. Overall, NFHS‐4 covered a total of 28,527 clusters from 640 districts and interviewed 811,808 male and female respondents; while NFHS‐5 encompassed 30,197 clusters from 707 districts in India, totaling 825,954 individuals (some larger districts were divided to create smaller districts, which increased the total number of districts). The individual‐level data sets for males and females were obtained from the DHS‐India database following the submission and approval of a project proposal (*DHS‐India*).

#### 2.1.2 Strategy to Ensure Survey Data Quality

Due to the sample size and complexity of the NFHS survey framework, detailed strategies were developed to minimize non‐sampling errors and ensure high data quality. Several comprehensive manuals were prepared to maintain uniform survey procedures across the subregions, monitored by various field agencies, senior project officers, consultants, and staff. To transfer field data daily, computer‐assisted personal interviewing (CAPI) was utilized, where data quality was thoroughly checked, and real‐time feedback was provided to the field agencies and teams. This survey framework included a provision for generating error messages to address internal inconsistencies in the data, allowing for immediate corrections. Experts designed this application to highlight any inconsistencies in the responses of completed interviews for instant checking and rectification. More importantly, the CAPI programs assist in generating field‐check tables on key indicators and individual‐level demographic information daily, which were reviewed by the Quality Assurance Team (QAT) in the central office to facilitate necessary feedback to the teams working in the field across different parts of the country.

#### 2.1.3 Socio‐Demographic Information

We analyzed NFHS data to gather demographic information about participants' gender, wealth index, and caste. The *wealth‐index* serves as a composite measure of an individual's overall living standard, calculated using data on participants' ownership of various consumer items, such as televisions and cars, as well as dwelling characteristics, including flooring materials, sources of drinking water, toilet facilities, and other socioeconomic and demographic factors related to wealth status. Each household from which asset information was collected was assigned a weight score generated through principal component analysis. Each individual received a standardized score for each asset, with scores varying based on whether they owned that particular asset. These individual scores were summed for each participant and then standardized according to the standard normal distribution. The sample was subsequently divided into five population quantiles: *richest*, *richer*, *middle class*, *poorer*, and *poorest* (with decreasing quantile estimates).

Based on the Indian *caste system* (Thapa et al., 2021), which is predetermined at birth (Ghurye, 1969), the survey identified individuals as *general*, *Other Backward Class (OBC)*, SC and ST. In our analysis, we combined the last two categories as SC + ST. We also aggregated statistics for *males* and *females* based on the relative proportions across the NHFS clusters.

### 2.2 Ambient PM_2.5_ Concentration

We relied on satellite‐derived ambient PM_2.5_ concentrations due to significant spatial gaps in the ground‐based monitoring network across India (Brauer et al., 2019). The detailed algorithm and error matrix of the satellite‐PM_2.5_ data set were detailed in our earlier work (Katoch et al., 2023). Briefly, we first filled the gaps in daily‐mean level‐2 aerosol optical depth (AOD) product from the Moderate Resolution Imaging Spectroradiometer (MODIS) using machine learning algorithm and then predicted daily‐scale (i.e., one value per‐day across each grid) ambient PM_2.5_ concentration at 1‐km × 1‐km spatial resolution. Extensive evaluation of satellite‐PM_2.5_ against coincident ground‐based measurements across India reveals robust performance (*R* ^2^ = 0.97 and RMSE = 10.35 µgm^−3^) (Figure S3 in Supporting Information S1), indicating that this data set can be utilized as ambient air pollution estimates for subsequent analyses. For this study, we aggregated daily ambient PM_2.5_ estimates to the annual scale corresponding to the NFHS survey periods. For instance, we used the annual average estimates of PM_2.5_ concentration from 2015 to 2016 to align with the NFHS‐4 time period and from 2019 to 2021 to match with NFHS‐5, ensuring consistency in the temporal resolution between the satellite‐derived air pollution estimate and the NFHS data sets. We assumed that respondents remained in their respective clusters throughout the survey duration and were continuously exposed to that cluster‐level chronic ambient PM_2.5_ exposure. This assumption enabled the temporal alignment between air pollution estimate and the demographic information of NFHS.

### 2.3 Weighted PM_2.5_ Exposure for Subgroups

We then extracted the annual mean PM_2.5_ concentration for each survey cluster (defined as urban or rural) by averaging PM_2.5_ concentrations within a radius of 2 km (for urban) and 5 km (for rural) surrounding their geo‐locations (centroid) using ArcGIS (*v3.8*) software. The subgroup‐weighted PM_2.5_ exposure (PWC) for a district and state was estimated through a weighted average of cluster‐level PM_2.5_ concentrations, integrated with the variation in population subgroup fractions across the clusters within the district and state. We incorporated the assigned cluster‐specific “*sample‐weight*” in our assessments, which signifies the proportion of the population subgroup (representative of census‐demography), within each cluster, as estimated by survey data. If there are *m* clusters in a region, PWC is defined as,

(1) $${PWC}_{ik}=\frac{\sum_{j=1}^{m}C_{ij}\times P_{ijk}}{\sum_{j=1}^{m}P_{ijk}}$$

Where *PWC*_*ik*_ represents the sub‐population weighted mean concentration of ambient PM_2.5_ for the *kth* population subgroup of the *ith* region, *C*_*ij*_ denotes the ambient PM_2.5_ concentration at the *jth* cluster of the *ith* region, and *P*_*ijk*_ indicates the estimated proportion of the *kth* population subgroup at the *jth* cluster of the *ith* region.

We accounted for the sample weight of every cluster reported by the NFHS to estimate PWCs. Sample weights were used to assess how representative the sample was of the actual census demographics of that region. For instance, a sample weight of 500,000 for a survey cluster represents a population of 500,000 from the census across India (*DHS‐India*). The estimated PWC also depends on the distribution of population subgroups within each cluster in a region, along with its respective PM_2.5_ concentration level. A higher estimated PWC of a subgroup within any region indicates that a larger proportion of that sub‐population resides in locations where the ambient PM_2.5_ concentration is higher within that geographic entity, and vice versa (Aunan et al., 2018).

We performed the Kolmogorov‐Smirnov (KS) test (Nahm, 2016) to assess the significance level (denoted by *p* < 0.1) of the difference among the subgroup‐specific PWCs in urban and rural regions. We quantified the number of districts in which the estimated PWC was higher (*p* < 0.1) among females, four wealth (richer, middle‐class, poorer, poorest), and two caste‐subgroups (OBC and SC + ST) as compared to their reference subgroup counterparts (males, richest, and general) across urban and rural regions.

### 2.4 Estimation of Absolute and Relative Disparity

We first used the Global Human Settlement Layer (GHSL) database to categorize the grids (1‐km × 1‐km) into urban and rural regions across India (*GHSL‐database*). The GHSL database classifies the grids into seven major categories: *city*, *dense town*, *semi‐dense town*, *suburban or peri‐urban area*, *village*, *dispersed rural area*, and *no settlement layer*, and is available from 1990 to 2020 at five‐year intervals. We combined the settlement layer information of the first four categories as “urban” and the latter three as “rural” for 2015 and 2020 (concurrent with the NFHS time‐points) and integrated it with satellite‐derived air pollution estimates to obtain the distributions over urban and rural regions of India and across the states. We estimated absolute disparity by considering the difference between the 99th (highest value) and 1st (lowest value) percentiles (excluding outliers in this analysis) of the ambient PM_2.5_ concentration distribution among the overall population and individuals within each subgroup.

We categorized the subregions into three socio‐demographic indices (SDIs): low SDI (≤0.53), middle SDI (0.54–0.6), and high SDI (>0.6), as presented in the GBD‐India using a combination of log‐distributed per‐capita income, mean education level (for those over 15 years), and fertility rate in women (<25 years).

Next, we estimated the relative disparity in terms of the effect size estimate or *Z*_score_ (Aoki, 2020),

(2) $$Z_{\text{score}}=\frac{\left(M_{1}-M_{2}\right)}{S_{\text{pooled}}},\;S_{\text{pooled}}=\sqrt{\frac{{{\left(n_{1}-1\right)\times S}_{1}}^{2}+\left(n_{2}-1\right)\times {S_{2}}^{2}}{n_{1}+n_{2}-2}}$$

We first estimated the district‐level PWCs across the subgroups and log‐transformed them to convert into a Z‐distribution (minimizing the heteroscedasticity in the distributions at higher PM_2.5_ level) (Feng et al., 2014). For two sub‐populations, *M*_1_ and *M*_2_ (in Equation 2) are the arithmetic means of the PWCs of subgroup_1_ and subgroup_2_, respectively; $S_{1}$ and $S_{2}$ denote the standard deviations of the ambient PM_2.5_ distribution for the two subgroups, with *n*_1_ and *n*_2_ being the sample sizes (the number of districts across India or within each state). When calculating the *Z*_score_, we compared each subgroup to its reference counterparts. For the four wealth subgroups, the reference was the richest subgroup; for the OBC and SC + ST subgroups, the reference was the *general* subgroup, with females being compared to their *male* counterparts. The pooled standard deviation (*S*_pooled_) was assessed to incorporate the differential standard deviations of the sub‐populations and minimize heterogeneity, consistent with Okun's approximation (Okun, 2015). A positive *Z*_score_ of +1 indicates that the estimated PWC of subgroup_1_ was higher by one pooled standard deviation compared to the estimated PWC of subgroup_2_, while negative values indicate the opposite (Aoki, 2020).

Previous *EJ* studies have employed various inequality metrics such as the Atkinson Index, Gini Coefficient, Theil Index, and Concentration Index (Malakar & Mishra, 2017; Rao et al., 2021; Rosofsky et al., 2018; Tian et al., 2021) to quantify exposure disparities among sub‐populations. However, these measures primarily capture the magnitude of disparity and fail to identify which subgroups are disproportionately burdened. Additionally, they do not account for heterogeneity in subgroup‐specific exposure distributions, such as differences in standard deviations. To our knowledge, the application of the *Z*_score_ metric to assess exposure disparities across demographic subgroups is novel and never been used before in the existing inequality‐assessment frameworks. This approach offers dual advantages: it not only highlights inequality hotspots‐regions where disparities are particularly pronounced, but also identifies the subgroups more burdened by higher PWC, based on the magnitude and direction (*sign*) of the *Z*_score_, respectively (Aoki, 2020). We reported the estimated relative disparities in ambient PM_2.5_ exposure at the national, three SDI subregions, and state levels across the demographic subgroups. In the main text, we presented the mean *Z*_score_ estimates among each demographic subgroup‐pairs and identified the states where the mean standardized differences in PWCs satisfied the significance threshold of *p* < 0.1. In a sense, we rejected the *null hypothesis* with 90% confidence, indicating that the estimated PWCs between the sub‐populations of concern were statistically different and exposure disparity exists. The corresponding *p*‐values associated with the mean *Z*_score_ estimates are provided in Figure S4 of Supporting Information S1. We divided the state‐level estimates of *Z*_score_ among the sub‐populations by their national‐level estimate to determine whether the disparity increases (i.e., ratio >1) when the spatial scale of analysis changes from the national to subnational level (Figure S5 in Supporting Information S1). In the NFHS framework, buffer radii of 2 km for urban clusters and 5 km for rural clusters are conventionally applied to generate geospatial buffers. This convention reflects the higher density and smaller spatial extent of urban clusters, compared with the larger, more spatially dispersed rural clusters. A smaller urban buffer minimizes the inclusion of surrounding areas that may not represent the local exposure environment, whereas a larger rural buffer accounts for sparser settlement patterns and the need to capture representative ambient conditions (*DHS‐India*). To maintain consistency with this convention, our primary analysis applied a 2 km radius to urban clusters and a 5 km radius to rural clusters. To evaluate the robustness of our spatial exposure assessment, we conducted sensitivity analyses in which these radii were varied, and the resulting mean PWCs and *Z*_score_ estimates were compared with baseline values (following the NFHS framework). In the 1st sensitivity analysis, a uniform 2 km radius was applied to both urban and rural clusters; in the 2nd sensitivity analysis, a uniform 5 km radius was applied to both. We presented the results from our sensitivity analyses at the national level, across the three SDI subregions, and at the state level in Tables S1–S5 of Supporting Information S1.

In India, sub‐population‐level environmental, social, and health policies are often designed with a specific focus on either urban or rural regions within the states or subregions (Balakrishnan et al., 2019; Katoch et al., 2023). To ensure alignment with the spatial scope of these policy frameworks, our exposure disparity assessments were conducted over urban and rural areas at the state level, enhancing the relevance and applicability of the findings for targeted, policy‐driven interventions. To quantify intra‐state variation in ambient PM_2.5_ concentrations (*absolute disparity*), we used the GHSL database, while the demographic information from the NFHS was employed to assess sub‐population‐weighted exposure disparities across demographic subgroups (*relative disparity*). Note that the GHSL and NFHS data sets were used independently to classify urban and rural grids or clusters, respectively, and each was separately integrated with satellite‐derived ambient PM_2.5_ estimate. However, these two data sets were not combined in our analysis to categorize urban/rural regions. Although GHSL and NFHS apply different methodological frameworks to classify grids or clusters as urban and rural, we obtained a good agreement (*r* = 0.59, RMSE = 0.21; Figure S6 in Supporting Information S1) between the state‐level urban fractions derived from the GHSL and the sample‐weighted estimates from NFHS across survey rounds. Additionally, we conducted a concordance test to evaluate the agreement between NFHS and GHSL cluster classifications (urban and rural) across India over the NFHS time points. The analysis revealed higher diagonal proportions, with concordance coefficients of 0.717 (between GHSL‐2020 and NFHS‐5) and 0.714 (between GHSL‐2015 and NFHS‐4) (Table S6 in Supporting Information S1), indicating stronger alignment between urban‐urban and rural‐rural coincident geo‐locations in these data sets. This concordance suggests that both data sets reliably capture the spatio‐temporal heterogeneity of urban‐rural distribution across Indian states, and justifies our approach of using GHSL data set to estimate absolute disparities across the states.

## 3 Results

### 3.1 National Level Disparity in Ambient PM_2.5_ Exposure Across the Subgroups

Satellite‐derived ambient PM_2.5_ exposure ranged from 28.6 to 86 µgm^−3^ (mean: 51.5±16.7 µgm^−3^) in urban regions and from 30 to 80.6 µgm^−3^ (mean: 53±16.3 µgm^−3^) in rural areas during NFHS‐5 (2019–2021) (Table S7 in Supporting Information S1). At the national level, we observed higher PWC among the top (richest, 52.5–55.5 µgm^−3^) and bottom (poorest, 56.1–58.8 µgm^−3^) quantiles of the wealth index in both urban and rural regions compared to other wealth subgroups (Table 1). The general subgroup experienced higher ambient PM_2.5_ levels (55.5–56.5 µgm^−3^) than their demographic counterparts in both regions. However, the disparity in ambient air pollution was minimal between males and females in both areas. Overall, the disparities in estimated PWCs among the wealth subgroups were greater in both regions (*Z*_score_ > ±0.09). However, at the national level, we found moderate disparity (*Z*_score_ > ±0.04) among the caste subgroups.

**Table 1:** Assessment of Ambient PM_2.5_ Exposure Across the Population Subgroups Stratified by Gender, Wealth‐Index, and Caste Subgroups of NFHS‐5

Urban region								
		Ambient PM_2.5_			Population fraction (%) of subgroup			
Population subgroups		PWC (CV)	Z_score_ (95% CI)	Sub‐population fraction (in %)	>66th percentile (>67.3 µgm^−3^)	34th–66th percentile (40.8–67.3 µgm^−3^)	<34th percentile (<40.8 µgm^−3^)	No. of districts
Gender	Male	51.7 (0.336)	Reference	52	51.7	57.2	48.8	Reference
	Female	51.6 (0.334)	0.0025 (0.0019–0.0031)	48	48.3	42.8	51.2	250 (692)
Wealth	Richest	52.5 (0.317)	Reference	19.5	22.43	17.41	19.24	Reference
	Richer	50 (0.314)	0.0915 (0.0677–0.1153)	27.8	26.61	27.99	29.11	111 (449)
	Middle‐class	51 (0.315)	0.0943 (0.0688–0.1198)	24	22.53	24.51	25.38	117 (449)
	Poorer	53.3 (0.313)	−0.0965 (−0.0772–0.1158)	18	17.04	19.02	17.54	113 (449)
	Poorest	56.1 (0.316)	−0.0119 (−0.0092–−0.0146)	10.7	11.4	11.06	8.73	102 (449)
Caste	General	55.5 (0.325)	Reference	42.2	29.95	33.03	28.6	Reference
	OBC	49.7 (0.323)	0.0464 (0.0325–0.0603)	30.8	47.09	37.25	45.58	267 (624)
	SC + ST	51.4 (0.322)	0.0537 (0.0376–0.0698)	27	22.96	29.73	25.83	295 (624)
**Rural region**								
		**Ambient PM**_**2.5**_ **estimates**			**Population fraction (%) of subgroup**			
**Population subgroups**		PWC (CV)	*Z*_score_ (95% CI)	**Sub‐population fraction (in %)**	>66th percentile (>60.8 µgm^−3^)	34th–66th percentile (37.6–60.8 µgm^−3^)	<34th percentile (<37.6 µgm^−3^)	**No. of districts**
Gender	Male	54.2 (0.310)	Reference	51	49.2	52.4	52.2	Reference
	Female	55.3 (0.311)	−0.0438 (−0.0333–0.0543)	49	50.8	47.6	47.8	335 (689)
Wealth	Richest	55.5 (0.314)	Reference	12.2	12.5	12.14	11.46	Reference
	Richer	51.2 (0.31)	0.1138 (0.0956–0.132)	19.9	17.42	21.35	24.18	313 (649)
	Middle‐class	52.3 (0.312)	0.0917 (0.0724–0.111)	25.2	23.2	25.82	30.6	290 (649)
	Poorer	56.6 (0.319)	−0.0148 (−0.0121–0.0175)	26	27.63	25.21	22.7	287 (649)
	Poorest	58.8 (0.322)	−0.0671 (−0.0537–0.0805)	16.7	19.24	15.49	11.05	283 (649)
Caste	General	56.5 (0.302)	Reference	23.8	22.41	25.44	24.37	Reference
	OBC	56.3 (0.305)	0.0647 (0.0472–0.0822)	43.6	47.01	39.62	44.86	322 (633)
	SC + ST	53.4 (0.303)	0.0028 (0.0024–0.0032)	32.6	30.58	34.94	30.77	288 (633)

*Note.* “CV” signifies the Coefficient of Variation In the “*No. of districts*” columns, the numbers denote the number of districts where the corresponding subgroups breathed in higher ambient PM_2.5_ concentration (*p* < 0.1) as compared to their reference subgroup counterparts. The numeric values in parentheses indicate the number of districts (out of 707) where the standardized *Z*_score_ estimates have p‐values within the considered significance threshold (*p* < 0.1).”

We estimated that wealthier subgroups were less likely to reside in cleaner urban areas. For instance, the proportions of the richest and richer subgroups were higher in urban areas where the ambient PM_2.5_ level was elevated (>66th percentile of the distribution). However, we found higher PWCs for poorer and poorest subgroups in rural regions. The richest and poorest subgroups have the smallest population shares across the states but are exposed to high levels of air pollution, particularly in urban areas (Table 1). The OBC subgroup was more prevalent in both high and moderate air pollution regions (>34th percentile), followed by the general and SC + ST subgroups across urban and rural regions, respectively. We found that females, the other four wealth subgroups, and two caste subgroups inhaled lower PM_2.5_ concentrations than their reference subgroup counterparts in the urban regions of several districts (Table 1). However, we observed the opposite trends in the rural areas of a greater number of districts.

During NFHS‐4, mean air pollution exposure [urban: 57.7 ± 18.8 µgm^−3^ (range: 31.2–93.2 µgm^−3^); rural: 58 ± 18 µgm^−3^ (range: 30.9–86.8 µgm^−3^)] was higher across the states compared to recent years (NFHS‐5) (Table S8 in Supporting Information S1). However, we observed similar distributions of PWCs across the sub‐populations in both NFHSs. At the national level, we estimated higher PWCs among the richest and poorest sub‐populations (57–66 µgm^−3^), as well as among general and OBCs (59–64 µgm^−3^) compared to the middle‐class and SC + ST subgroups. A larger fraction of wealthier subgroups, general and OBC individuals breathed in higher levels of ambient PM_2.5_ in urban regions, whereas their subgroup counterparts predominantly occupied rural areas with higher PM_2.5_ levels. Similar to NFHS‐5, we found higher PWCs among males, the richest and general subgroups compared to their demographic counterparts in both regions of most districts.

### 3.2 Urban‐Rural Heterogeneity in Ambient PM_2.5_ Exposure

**Figure 1:**
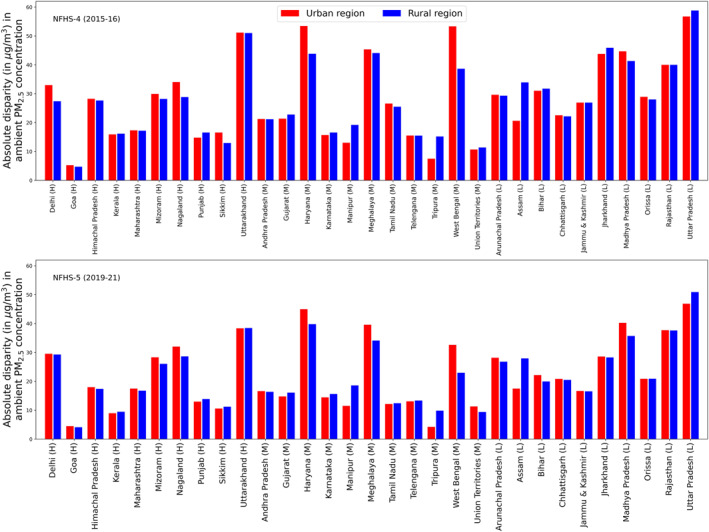
Estimated absolute disparity (AD, in µgm^−3^) in ambient PM_2.5_ concentration across urban and rural regions of the states (*top panel* for NFHS‐4 and *bottom panel* for NFHS‐5). The states are arranged in alphabetic order and classified into high (H), middle (M), and low (L) SDI categories.

There is an absolute disparity in ambient PM_2.5_ exposure (the difference between the 99th and 1st percentile estimates) among the overall population, varying across high, middle, and low socio‐demographic index (SDI) states and demonstrating urban‐rural heterogeneity. During NFHS‐5, we observed a larger disparity in the urban areas of Haryana, Madhya Pradesh, Meghalaya, and Uttar Pradesh (42–45 µgm^−3^); whereas the disparity was greater in the rural regions (33–48 µgm^−3^) of Uttar Pradesh, Uttarakhand, and Haryana (Figure 1, *bottom panel*). The absolute disparity was more pronounced across larger geographic states in central and northern India, while substantial urban‐rural heterogeneity was found among the southern peninsular states. During NFHS‐4, the absolute disparity was higher (by 10–15 µgm^−3^) in both regions compared to NFHS‐5 (Figure 1, *top panel*), although the pattern of absolute disparity remained consistent across the states. We conducted a sensitivity analysis to estimate absolute disparities in ambient PM_2.5_ exposure across states using the urban‐rural cluster information from NFHS. As shown in Table S9 of Supporting Information S1, the results for urban and rural regions were highly comparable, supporting the robustness of our approach and justifying the use of the GHSL data set for urban‐rural classification.

We estimated that the urban‐rural disparity in ambient PM_2.5_ concentration among the overall population decreased from NHFS‐4 to NHFS‐5. Previous studies from developed countries have reported that spatial variations in air pollution levels and demographic distribution are key drivers of the disparity in ambient PM_2.5_ across sub‐populations and regional heterogeneity. Over these time points, the demographic distribution of the subgroups across the subregions did not change substantially (Figure S7 in Supporting Information S1). However, air pollution levels declined across much of India during the NFHS‐5 time point, primarily due to abrupt emission reductions associated with the year‐long COVID‐19‐induced stricter lockdown, alongside the effective implementation of stricter air pollution mitigation policies and favorable meteorological conditions (Katoch et al., 2023; Xie et al., 2024). Hence, the reduction in absolute disparity and its urban‐rural heterogeneity in most states could be attributed to ambient PM_2.5_ abatement. Interestingly, we observed a higher absolute disparity in a few smaller states, namely Uttarakhand, Meghalaya, Haryana, Nagaland, and Mizoram (30–40 µgm^−3^), which is consistent with greater heterogeneity (high standard deviation) in the demographic distribution of the subgroups (Figure S8 in Supporting Information S1), despite the lower levels of air pollution in these subregions.

### 3.3 Identifying the Subgroups Burdened by Higher Ambient Air Pollution

**Figure 2:**
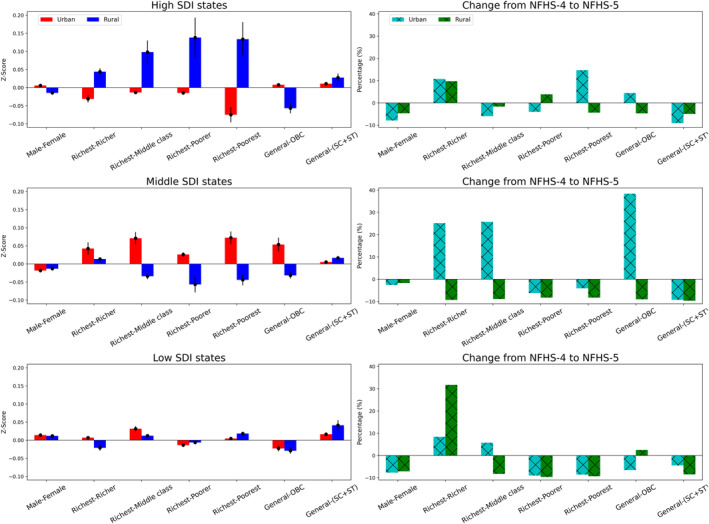
Estimated relative disparity (denoted by *Z*_score_) across the subgroups in the urban and rural regions in high, middle, and low SDI states in NFHS‐5 (*left column*); and its changes (in‐%) from NFHS‐4 (*right column*). For this assessment, we considered males, richest, and general subgroups as the reference to estimate the difference in mean PWCs across their demographic counterparts. For inference, a positive *Z*_score_ depicts that the estimated PWC in any of the reference subgroups is higher than its demographic counterpart, normalized by their pooled standard deviation. Similarly, a negative *Z*_score_ infers the opposite. The *cyan* and *green* hatched bars depict the %‐changes in relative disparity (change in absolute values of *Z*_score_) from NFHS‐4 (2015–2016) to NFHS‐5 (2019–2021).

In this section, we demonstrated the relative disparity among high, middle, and low SDI states and identified the subgroups burdened by higher ambient PM_2.5_ levels across urban and rural regions. In urban areas, we estimated the largest relative disparity within the subgroups of middle SDI states, followed by high and low SDI subregions (Figure 2, *left* column). However, the relative disparity decreased from high to low SDI states in rural areas. In the urban regions of middle SDI states, the wealthiest subgroup faced higher ambient air pollution (*Z*_score_ of 0.073, *p* < 0.1), with relative disparity increasing (from 0.026 to 0.073) as the wealth‐index disparity grew among sub‐populations (from richest‐richer to richest‐poorest). In high SDI states, the deprived subgroups endured higher ambient PM_2.5_ concentrations (0.015–0.075).

Across the caste subgroups, general urbanites were more densely populated in the polluted areas of high and middle SDI states (0.011–0.054) compared to their demographic sub‐populations. Conversely, in rural regions, relative disparity increased (0.044–0.134) as the wealth‐index gap widened across high SDI states. However, deprived sub‐populations faced a greater burden (−0.044 to −0.057) from air pollution in middle SDI subregions. Furthermore, we observed a higher PWC among the OBC subgroup (−0.029 to −0.057) than among the general group across the three SDI states, while the general subgroup experienced greater PM_2.5_ exposure (0.017–0.041) than the SC + ST. In contrast, the male‐female disparity in ambient PM_2.5_ levels was lower across the three SDI states.

The right column (Figure 2) illustrates changes in relative disparity (changes in absolute *Z*_score_ estimates) over different time points. In middle SDI states, relative disparities between the richest‐richer and the richest‐middle class increased substantially in urban areas (26%–28%), while disparities among the richest and deprived subgroups decreased. The general‐OBC relative disparity also rose considerably (38%) in urban regions, while it fell in rural areas. In rural areas of middle and low SDI states, we observed a reduction in relative disparity across most wealth and caste subgroups, except for the richest and richer subgroups, which increased by 32%. In high SDI states, we noted mixed trends in changes in relative disparity across both regions. Furthermore, the male‐female disparity decreased (5%–8%) over the time points across all SDI states.

**Figure 3:**
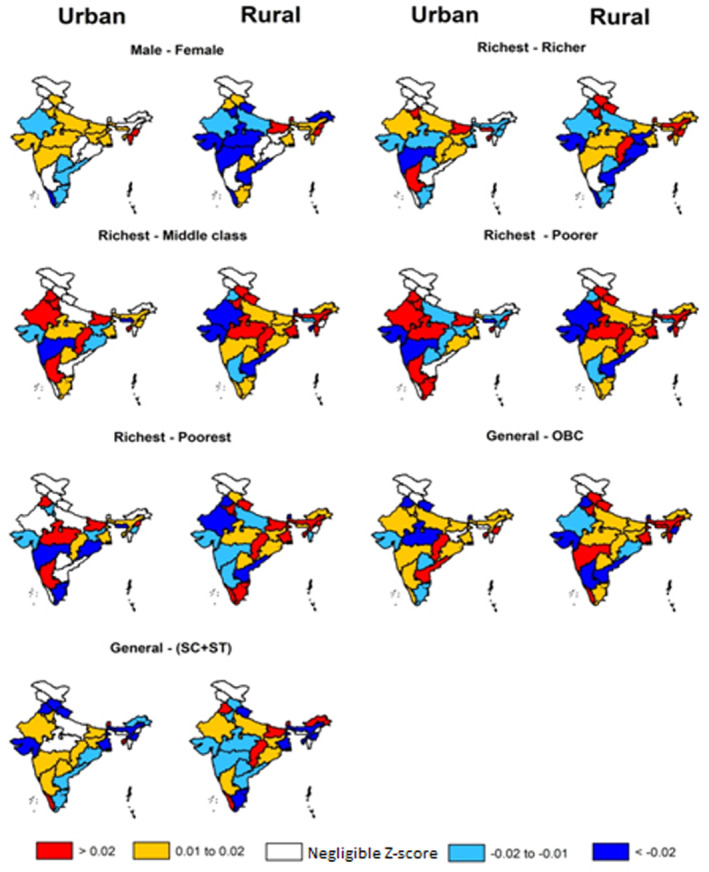
Estimated state‐level relative disparity (denoted by *Z*_score_) across the subgroups stratified by gender, wealth, and caste in the urban and rural regions across the states in NFHS‐5. States highlighted with *colored*‐background represent regions where the mean *Z*_score_ estimates (standardized mean differences in PWCs between sub‐populations) indicate statistically significant disparity (*p* < 0.1), rejecting the *null hypothesis* of equal or comparable PWCs at the 90% confidence level. For better interpretation, we considered a mean *Z*_score_ difference within ±0.01 standard deviations between the estimated PWCs of two subgroups as negligible, and accordingly, these states were highlighted with a *white*‐background. Figure S9 and S10 in Supporting Information S1 depict the *Z*_score_ estimates for NFHS‐4 and the change in relative disparity over the time points, respectively.

Next, we demonstrated the relative disparity at the state level (Figure 3), where deprived sub‐populations (the poorer and poorest) suffered from higher ambient PM_2.5_ levels (*Z*_score_: −0.273, *p* < 0.1) than their richer counterparts in the urban regions of central and southern peninsular states (e.g., Maharashtra and Karnataka) and in the rural regions of western states. Conversely, higher PWC was estimated among the richest subgroup (0.099) in the rural regions of the northeast and IGP states, and in the urban regions of central, western, and southern states. Moreover, we estimated a higher PWC among the richest (0.246) than the middle‐class subgroup, especially in rural areas. However, the disparity in PWCs among the richest and richer was minimal across most of the states.

Among the caste subgroups, we estimated higher PWC in general subgroup as compared to the OBC across most of the states (0.266); whereas SC + ST subgroup suffered from higher PM_2.5_ concentration than the general in the rural areas of NE, central, and southern peninsular states (−0.065). However, we estimated opposite patterns in estimated PWCs between general and SC + ST subgroups across the urban and rural regions in many states (Figure 3). Among males and females, we estimated higher PWC in males in the urban regions of the states; while the PWC was higher among females in the rural areas. We estimated that the states with larger geographic area, that is, Maharashtra, Uttar Pradesh, Rajasthan, Madhya Pradesh, and Chhattisgarh, experienced comparatively higher inequality (>±0.02) in the distribution of ambient PM_2.5_ concentration across most of the subgroups.

Our sensitivity analyses of varying cluster radii (2 and 5 km for both urban and rural clusters, respectively) have shown comparable *Z*_score_ estimates across the population subgroups, as compared to their baseline estimates following the conventional NFHS framework (Tables S1, S3 and S5 in Supporting Information S1). Note that in some subgroups, the sensitivity analyses produced *Z*_score_ estimates with absolute magnitudes comparable to their baseline estimates but with opposite signs. We hypothesized that this reversal in direction occurs when baseline subgroup differences are small or closer to zero, making them sensitive to modest shifts in assigned exposure values resulting from changes in buffer radii. Such shifts are most likely due to clusters located near sharp exposure gradients (e.g., peri‐urban or mixed‐use areas), where the inclusion or exclusion of nearby high‐ or low‐concentration grids can alter the relative ordering of subgroup mean PWCs (Clark et al., 2022). Importantly, the comparable magnitudes of the *Z*_score_ estimates across sensitivity settings indicate that the overall scale of subgroup disparities is preserved, confirming the robustness of our findings to variations in spatial exposure assignment.

From 2015–2016 to 2019–2021, we estimated that relative disparity among the wealth‐subgroups (poorest, poorer, and middle‐class) increased (>15%) as compared to the richest in the urban and rural regions of central, NE, and southern peninsular states, while reduced in the IGP states (richer and middle‐class subgroups relative to richest). Among the caste‐subgroups, relative disparity between general and OBC subgroups increased in the urban regions of IGP and central states; but substantially reduced in the rural areas of central and southern peninsular states (<20%). However, the disparity in estimated PWCs between the general and SC + ST sub‐populations increased in the rural regions of the northern states, while it substantially decreased in the urban areas across most states (Figure S10 in Supporting Information S1). We observed no considerable changes in relative disparity among the wealth subgroups in the urban and rural regions of Delhi and Goa, and among the caste subgroups in the northeastern states. Moreover, the change in relative disparity between males and females over time was minimal.

### 3.4 Variation in Ambient PM_2.5_ Exposure Across the Individuals of Each Subgroup

**Figure 4:**
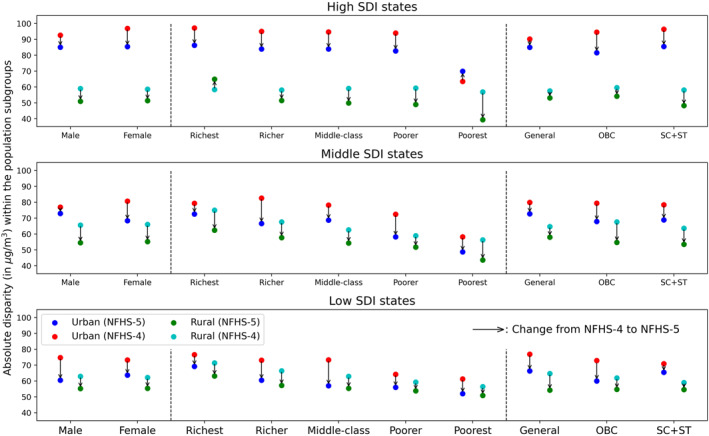
Absolute disparity in ambient PM_2.5_ concentration within each population subgroup across the urban and rural regions of the high (*top* panel), middle (*middle* panel), and low (*bottom* panel) SDI states. The direction of vertical arrows indicates the change in absolute disparity from NFHS‐4 (2015–2016) to NFHS‐5 (2019–2021).

We estimated absolute disparity within the subgroups across the high, middle, and low SDI states and found that individuals in each subgroup experienced different levels of ambient PM_2.5_ in urban and rural regions (Figure 4). The urban‐rural heterogeneity was greater in the high SDI states (10–15 µgm^−3^) compared to the middle and low SDI subregions. Consistent with the trends among the overall population, absolute disparity within all sub‐populations was higher during NFHS‐4 and declined thereafter; it was also larger in urban areas than in rural regions, especially in high (25–30 µgm^−3^) and middle (15–20 µgm^−3^) SDI states. Over time, we observed a larger reduction in absolute disparity in urban areas across most subgroups, possibly due to the effective implementation of urban‐centric mitigation measures during the national clean air program era (post 2017) (Ganguly et al., 2020; Katoch et al., 2023). In the middle and low SDI states, absolute disparity consistently reduced from the richest to the poorest subgroups in both regions, although the change was minimal in high SDI states.

Across the caste subgroups, SC and ST individuals experienced higher levels of ambient air pollution (absolute disparity of 95 µgm^−3^) in the urban areas of high SDI states compared to OBC and general individuals. However, we found similar absolute disparities among these subgroups in the urban regions of middle and low SDI states. In contrast, general individuals faced a broader variation (60 µgm^−3^) in ambient PM_2.5_ in rural areas, followed by OBC and SC and ST subgroups (50–55 µgm^−3^). Moreover, urban‐rural heterogeneity was minimal among male and female individuals across the three SDI states.

The subgroups that are exposed to relatively higher levels of air pollution in India may experience more severe acute to chronic health effects compared to their demographic counterparts. Evolving studies from the US and China have reported that these vulnerable subgroups, burdened by *higher‐than‐normal* ambient PM_2.5_ levels than other groups, face an increased risk (0.5%–1%) of developing various cardiovascular and respiratory diseases with each unit increase in PM_2.5_ (Letellier et al., 2022; Liu et al., 2021; Rosofsky et al., 2018; Yang et al., 2022). In the Indian context, further research is necessary to understand whether the differing distributions of ambient PM_2.5_ across subgroups, as seen in this study, may result in varying health effects for different non‐communicable diseases, ultimately contributing to disparities in air pollution‐related health burdens among the population.

## 4 Discussion and Conclusions

Environmental disparity refers to the condition in which various environmental risk factors, such as air pollution, disproportionately burden vulnerable subgroups compared to other segments of society (Boyce et al., 2016; Clark et al., 2014; Jbaily et al., 2022). Our research provides comprehensive national and sub‐national insights into disparities in ambient PM_2.5_ exposure among different socio‐demographic population subgroups in India, employing a novel application of the *Z*_score_ to quantify inequality between sub‐populations and its variation across NFHS time points. Most recent studies on environmental inequality have discussed the underlying components contributing to changing disparities over time (Bell & Ebisu, 2012; Clark et al., 2014; Tessum et al., 2022). The magnitude of disparity in ambient PM_2.5_ is strongly influenced by the degree of spatial variation in air pollution estimates and demographic distributions (Clark et al., 2014, 2017; Rafaj et al., 2018). We obtained a strong correlation between the coefficient of variation in ambient air pollution estimates and higher absolute as well as relative disparities across larger geographic states in India. Although the demographic distribution did not shift drastically, the change in ambient PM_2.5_‐attributable disparity among subgroups was strongly influenced by the variations in air pollution estimates over the time points.

In India, multiple environmental policies have been implemented to combat air pollution (Swaminathan et al., 2019); however, none of these have considered the disproportionate distribution of population‐weighted PM_2.5_ exposure among the subgroups. Identifying the sub‐populations that are exposed to higher ambient PM_2.5_ levels compared to their demographic counterparts and pinpointing the hotspots with considerable disparities are key innovations of this study, which would be extremely helpful to decision‐makers and stakeholders in achieving equity in the distribution of air pollution among population subgroups. Current air pollution mitigation mandates are urban‐centric in India (Ganguly et al., 2020; Katoch et al., 2023), where we estimate that the disparity attributable to ambient PM_2.5_ has increased across most subgroups (Figure 2), although overall PM_2.5_ levels have declined in India (Dey et al., 2020; Katoch et al., 2023). This paradox is largely attributable to spatial heterogeneity in pollution sources and uneven benefits of mitigation measures. Since, current air pollution policies are often city‐wide and sector‐specific (Balakrishnan et al., 2019; Katoch et al., 2023), without targeting intra‐urban inequalities. As a result, the distributional effects of air quality improvements remain skewed; disproportionately benefiting better‐serviced areas (e.g., green, relatively low‐traffic, and regulated construction areas), while leaving high‐exposure clusters behind (Bramble et al., 2023). These growing spatial and socio‐environmental segregations contribute to the observed increase in disparities among urbanites, despite national‐level improvements in air quality. Another important factor to consider is the substantial shift in sub‐population distributions resulting from migration and residential relocation during and after the COVID‐19 lockdown (Ilham et al., 2024). These changes disproportionately affected urban residents, altering both their exposure to air pollution and access to socioeconomic resources, thereby exacerbating disparities among its sub‐populations, especially across wealth‐ and caste‐based subgroups. This reverse trend of diminishing overall air pollution alongside increasing disparities among urban residents indicates the need to consider such disparities when implementing targeted air pollution mitigation measures. Furthermore, periodic updates of sector‐specific emission estimates are necessary to monitor changes in air quality at local and regional scales, along with the associated inequalities. Please note that our PWCs and the corresponding *Z*_score_ estimates for sub‐populations across urban and rural areas were derived at the population level. These values reflect how the spatial distribution of each demographic subgroup interacts with the ambient PM_2.5_ concentration patterns over larger geographic entities (states or subregions) and their changes over time points. Therefore, investigating individual‐ or household‐level factors such as urban infrastructure, housing conditions, or time‐activity patterns falls beyond the scope of this analysis to find the root‐cause of such observed disparity across the subgroups, as our approach does not incorporate micro‐level behavioral or structural determinants.

Our *Z*_score_ estimates reveal a contrasting pattern in air pollution‐attributable disparities across wealth subgroups. For instance, we observed higher PWCs among the top and bottom quantiles of wealth‐index in urban areas of high and middle SDI states, while the middle class and deprived subgroups experienced greater burdens in rural regions (Figure 3). Generally, a larger fraction of wealthier sub‐populations tends to reside densely in multi‐story, congested urban apartments and near major roads (Kayes et al., 2019; Yang et al., 2020). In contrast, marginalized subgroups, especially the poorest ones in urban areas, face higher PM_2.5_ levels due to poor sanitation, significant poverty, and other socioeconomic factors (Steib et al., 2023). The poorest urbanites are disproportionately exposed to higher PM_2.5_ exposure due to residential proximity to major emission sources such as heavily‐trafficked roads, industrial and informal waste burning areas, often driven by systemic socio‐spatial inequalities (Bramble et al., 2023; Jbaily et al., 2022). These neighborhoods frequently lack urban greenery and are more prone to pollution accumulation due to inadequate planning and infrastructure (Apte et al., 2017). Furthermore, limited access to cleaner cooking‐fuels, healthcare, and environmental protections exacerbates the health burden among these marginalized subgroups (deSouza et al., 2023). Table 1 indicates a higher‐than‐average proportion (11.4%) of the poorest sub‐population residing in highly polluted urban areas (>66th percentile) across India. This indicates that the combined burden of elevated pollution levels and sub‐population density has disproportionately increased the PWC among the poorest in urban regions, relative to other wealthier subgroups. In rural areas, subgroups with middle and lower wealth indices live in open environments and are more engaged in farming and daily wage commercial activities. However, they often reside close to emission sources from industries and thermal power plants, leading to increased exposure to polluted air from solid fuel use for domestic purposes (Agrawal et al., 2021). Our results align with existing literature, indicating that these subgroups are burdened by higher air pollution than their wealthier counterparts, particularly in the rural regions of middle and low SDI states. The general and OBC subgroups are predominantly urban, with their demographic distributions more widespread across India. In contrast, the SC + ST subgroup is primarily located in the outskirts and rural regions, especially the tribal subgroup (ST), which is heavily concentrated in forested states across central and eastern India (Figure S7D in Supporting Information S1). Due to their high population density in urban areas combined with higher pollution levels, the estimated PWCs are greater among the general and OBC sub‐populations compared to SC + ST.

In developed countries, ambient PM_2.5_ exposure has been consistently linked to unequal health burdens across demographic subgroups, stratified by race, wealth, and ethnicity. For instance, Di, Dai, et al. (2017) highlighted that such disparities translate into disproportionate health impacts among marginalized subgroups. While urban and rural exposure levels may be comparable, rural populations often face worse health outcomes due to lower income, education, healthcare access, and unhealthy lifestyles (Chafe et al., 2014). Studies have shown that minority subgroups, such as Mexican‐Americans, exhibit heightened susceptibility to traffic‐related air pollution and associated metabolic disorders (Zhang et al., 2020). Evidence from China similarly points to varied obesity and cardio‐metabolic risks across ethnicities due to differences in lifestyle and environmental exposure (Liu et al., 2021). Although many time‐series studies link short‐term air pollution exposure to acute health outcomes, research suggests stronger associations with long‐term exposure, particularly for mortality (Kloog et al., 2012). Importantly, most studies have focused on exposure disparities, leaving a gap in understanding how baseline health disparities and structural inequities amplify pollution‐related health burdens (Kerr et al., 2024). Recent US‐based analyses have begun quantifying differential mortality attributable to PM_2.5_ across sub‐populations, incorporating demographic and epidemiologic determinants (Geldsetzer et al., 2024; Kerr et al., 2024). In our subsequent work, we aimed to quantify the disparity in health burdens attributable to chronic exposure of ambient PM_2.5_, arising from the underlying exposure disparity and its associated differing health effects. Briefly, we employed a hierarchical logistic mixed‐effects model to estimate the associations between ambient PM_2.5_ concentrations and the odds of diabetes, high blood pressure, and heart disease reported in the NFHS (pooling the data sets of 4th and 5th rounds). These associations were stratified by diverse socioeconomic and demographic subgroups across gender, wealth‐index, and caste; while adjusted for relevant individual‐ and household‐level socioeconomic and demographic covariates. The resulting subgroup‐specific risk estimates were then integrated with corresponding demographic and epidemiologic attributes to quantify the disproportionate burden of PM_2.5_‐related health outcomes across these sub‐populations.

In the US, the residential distribution is more demarcated across the ethnic and racial subgroups. Studies have reported considerably larger disparities in ambient PM_2.5_ among demographic sub‐populations (Bullock et al., 2018; Clark et al., 2017, 2022; Rosofsky et al., 2018; Salazar et al., 2019). We observed such a pattern among high‐SDI states, characterized by greater urban‐rural heterogeneity and disparities within population subgroups (Figure 2). In middle and low‐SDI states, we estimated lower disparities across subgroups, primarily due to mixed neighborhoods where ambient PM_2.5_ is more uniformly distributed (Gaikar, 2020; Ram & Yadav, 2024; Sahasranaman & Bettencourt, 2019). We estimated higher PWCs among wealthier subgroups in urban areas, a finding that contradicts results from developed countries. Recent studies in China have indicated a positive correlation between rising PM_2.5_ levels and increased socioeconomic status among urban residents (Liu, Wang, et al., 2023; Liu, Liu, et al., 2023; Wang, Apte, et al., 2022; Wang, Wang, et al., 2022). Indian cities are currently experiencing accelerated growth, similar to Chinese urban settings, which may lead subpopulations to face higher point‐source attributed ambient PM_2.5_ concentrations. We found an increase in relative disparity (ratio >1 for both NFHSs) across most states as our spatial analysis shifted from the national level to the sub‐national level (Figure S5 in Supporting Information S1), particularly among middle and low‐SDI states. Our sensitivity analyses, using varying radii (2 and 5 km for both urban and rural clusters, respectively), indicated that relative disparities among sub‐populations diminished modestly when a coarser spatial resolution (5 km) was applied as compared to the assessments using 2 km geospatial buffers (Tables S1–S5 in Supporting Information S1). These results confirm that the broader heterogeneity in demographic composition and ambient PM_2.5_ distribution is more accurately captured at the finer NFHS cluster resolution, while the observed trends remain directionally consistent across buffer settings. This consistency demonstrates the robustness of our exposure assignment and supports the validity of our chosen buffer radii.

### 4.1 Policy Implications and Recommendations

The identification of subgroup‐specific disparities in ambient PM_2.5_ exposure calls for an urgent recalibration of India's air quality management strategies to align with equity‐focused environmental governance. Existing frameworks like the National Clean Air Programme (NCAP) have emphasized city‐level emission reduction targets but lack provisions to address intra‐urban or subgroup‐level disparities (Ganguly et al., 2020). We recommend integrating exposure equity indicators such as subgroup‐specific PWCs or the state‐level *Z*_score_ into performance assessments and funding allocation within NCAP and state action plans. Targeted interventions should prioritize pollution hotspots that disproportionately burden the OBC and deprived subgroups, including investment in localized monitoring, clean transportation infrastructure, and urban greening in marginalized neighborhoods (Apte et al., 2017). Furthermore, environmental regulations must account for the co‐location of vulnerable subgroups near industrial clusters, solid‐fuel hotspots, and informal waste burning sites, especially in the middle and low SDI states. Strengthening institutional coordination between health, urban development, and environmental ministries is also essential to embed health equity within air quality policy, thereby maximizing public health co‐benefits and supporting India's progress toward accomplishing several SDG targets.

International experiences reinforce the value of equity‐centered air quality policies and regulatory mandates. In the US, studies have led to the development of Environmental Justice Screening Tools (EJST) and regulatory frameworks that explicitly monitor and mitigate air pollution disparities among racial and income groups (Bramble et al., 2023; Jbaily et al., 2022). Similarly, China has begun incorporating socioeconomic vulnerability metrics into urban air quality planning, particularly after identifying higher exposure among lower‐income subgroups in rapidly expanding cities (Tu et al., 2022). Drawing from such examples, India could establish a national air pollution disparity surveillance system that routinely tracks exposure across demographic subgroups, supporting evidence‐based interventions and adaptive policy feedback.

Currently, health is not an integral part of the NCAP framework (Katoch et al., 2023). The success of the clean‐air action plan implementation should be evaluated in light of the anticipated health benefits in the future. Therefore, the forthcoming environmental policies to curb air pollution must address the disparities in ambient PM_2.5_ distribution and the related health effects across the states, especially as India aims to achieve environmental and health equity and the Sustainable Development Goals in the near future. Emissions from various primary sources contribute to ambient PM_2.5_; however, their impacts vary locally to sub‐regionally (Chatterjee et al., 2023; Chowdhury et al., 2019; McDuffie et al., 2021; Purohit et al., 2019). Future studies should consider the contributions from these diverse sources to conduct detailed source apportionment assessments, aiming to develop an integrated air pollution exposure database. This database could enable more accurate and comprehensive disparity estimates across subgroups. The lessons learned in India could help other developing countries implement effective air pollution mitigation strategies, thereby reducing substantial health burdens associated with air pollution for vulnerable subgroups.

We want to discuss a few points before concluding. *First*, we assigned the same ambient PM_2.5_ concentration to different subgroups residing within the buffer of each NHFS cluster. Such scale limitation may underestimate the disparity as studies have reported increasing disparity in ambient air pollution with increased spatial scale of air pollution exposure estimates (Chambliss et al., 2021; Parvez & Wagstrom, 2020). Due to current limitations in having fine‐scale or household‐level ambient PM_2.5_ concentration and census‐derived demographic information, the estimated PWCs for sub‐populations were inherently more aggregated, which might have attenuated the observed differences when analyzed at the state level. To address this constraint and more effectively capture relative disparities between urban and rural regions, we adopted a relaxed significance threshold (*p* < 0.1) and interpreted the mean *Z*_score_ estimates with 90% confidence. This can be addressed in future by using sensors at a hyperlocal scale. *Secondly*, we combined the seven settlement classes from the GHSL into broader urban and rural categories for our absolute disparity assessment. While we acknowledge that some peri‐urban grids might be misclassified, this could introduce moderate bias into disparity estimates. However, the high diagonal coefficients observed between NFHS and GHSL classifications (Table S6 in Supporting Information S1) indicate the robustness of our approach and suggest that such misclassification is limited in scope. Furthermore, our sensitivity analysis estimating absolute disparities across the states using NFHS clusters yielded highly comparable results for both urban and rural regions (Table S9 in Supporting Information S1). These strong agreements suggest that both data sets reliably capture the spatio‐temporal heterogeneity of urban‐rural distributions even at finer subregional‐scale across India, thereby minimizing potential classification‐bias at the cluster or subregional level. *Third*, we restricted our disparity assessment to ambient air pollution only. In India, a large fraction of the population continued to be exposed to high levels of household PM_2.5_ (Balakrishnan et al., 2019). Disparity in household exposure will be addressed separately. And *finally*, the demographic characteristics captured in the last census were used as the baseline for the sampling framework in recent NFHSs. We used the cluster‐specific sample weight in our analysis to make the assessment best representative of the changing demography of the population. The future NFHS sampling strategy based on the demographic distributions being captured in the ongoing census of India will further reduce uncertainty in disparity analysis.

In conclusion, while ambient PM_2.5_ exposure declined across India between the last two NFHS rounds, exposure disparity increased in urban areas and remained largely unchanged in rural regions. The rise in urban disparity was primarily driven by growing spatial and socio‐environmental segregation, uneven access to pollution mitigation benefits, lifestyle shifts, and large‐scale migration and demographic relocation during and after the COVID‐19 lockdown. In contrast, these socioeconomic and demographic factors remained relatively stable and evenly distributed among rural populations. This urban‐rural disparity was more pronounced in economically affluent states. Additionally, exposure disparities were higher among both the wealthiest and poorest subgroups, as well as among individuals from other backward classes.

## Conflict of Interest

The authors declare no conflicts of interest relevant to this study.

## Supporting information

Supporting Information S1

## Data Availability

The raw datasets used for disparity assessments and its associated analyses in this study are available at (Debajit & Sagnik, 2025).
