# Supplementary material for: Contrasting Patterns in Ambient PM2.5 Exposure Disparity Across Population Subgroups in Urban and Rural India
Source: Geohealth. 2026 Jan 24;10(1):e2025GH001387. doi: 10.1029/2025GH001387 (PMC12831208; doi:10.1029/2025GH001387)
Supplement: Supplementary file 1 — Supporting Information S1 [file GH2-10-e2025GH001387-s001.pdf]

Supplementary Information

Contrasting patterns in urban-rural heterogeneity in ambient $PM_{2.5}$ exposure disparity across population subgroups in India

Debajit Sarkar[1], #Alok Kumar[1], #Fahad Imam[1], Santu Ghosh[2], Julian D. Marshall[3], Joshua Apte[4], Luke D. Knibs[5], Pallavi Pant[6], Yang Liu[7], *Sagnik Dey[1,8,9]

[1]Centre for Atmospheric Sciences, Indian Institute of Technology Delhi, New Delhi, India
[2]St. John's Medical College, Bangalore, India
[3]Department of Civil and Environmental Engineering, University of Washington, Seattle, USA
[4]Department of Civil and Engineering, University of California Berkeley, USA
[5]School of Public Health, University of Sydney, Australia
[6]Health Effects Institute, Boston, MA, USA
[7]Rollins School of Public Health, Emory University, GA, USA
[8]Adjunct Faculty, Korea University, Seoul, South Korea
[9]School of Public Policy, Indian Institute of Technology Delhi, India

***Correspondence Author:** sagnik@cas.iitd.ac.in
#Equal contributions

This supplementary section contains 20 Figures and 42 Tables.

High SDI states (H): Delhi, Goa, Himachal Pradesh, Kerala, Maharashtra, Mizoram, Nagaland, Punjab, Sikkim, and Uttarakhand.
Middle SDI states (M): Andhra Pradesh, Gujarat, Haryana, Karnataka, Manipur, Meghalaya, Tamil Nadu, Telengana, Tripura, and West Bengal.
Low SDI states (L): Arunachal Pradesh, Assam, Bihar, Chhattisgarh, Jammu & Kashmir, Jharkhand, Madhya Pradesh, Orissa, Rajasthan, and Uttar Pradesh.

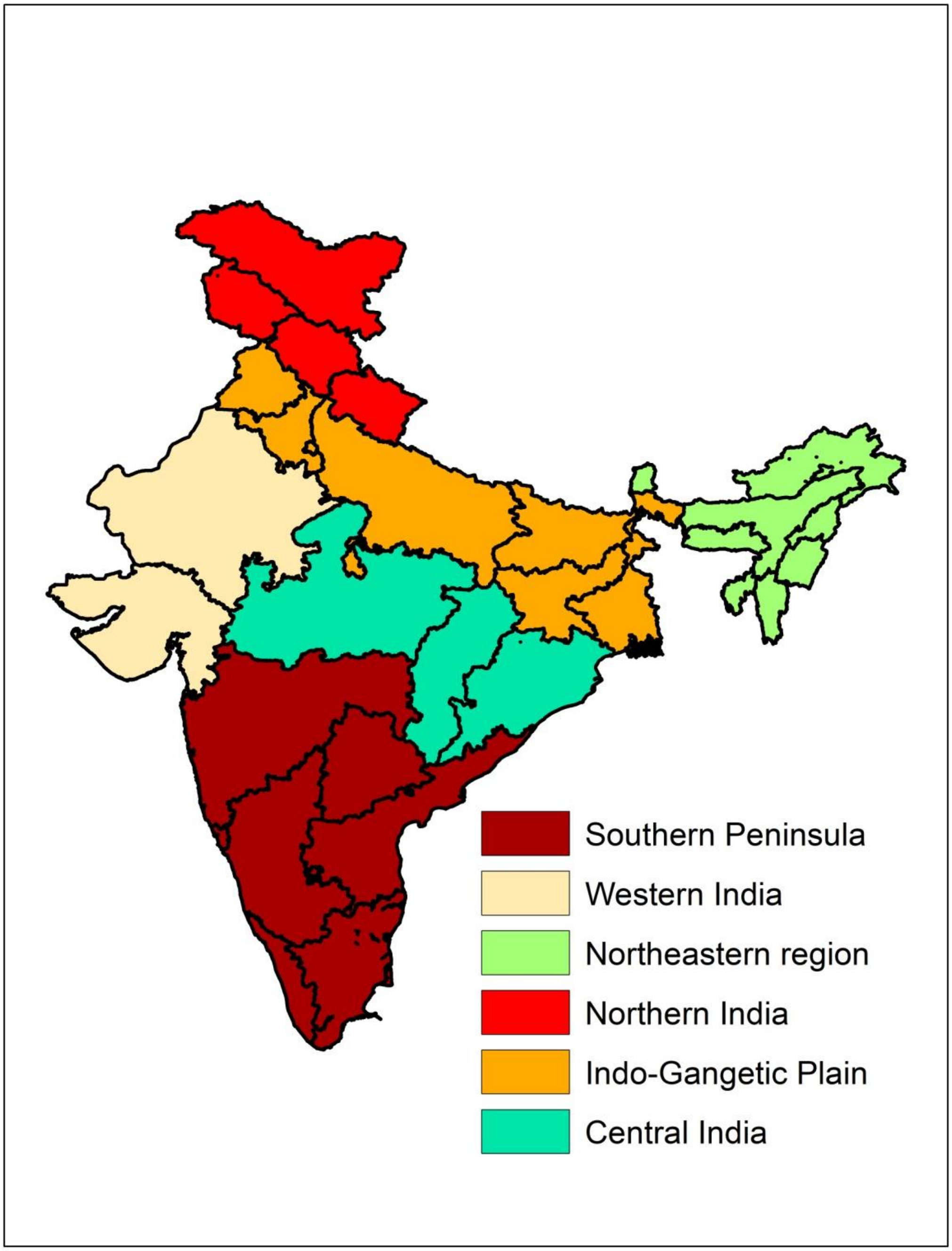

Figure S1: Geographic regions of India, demarcated by different *color-codes*.

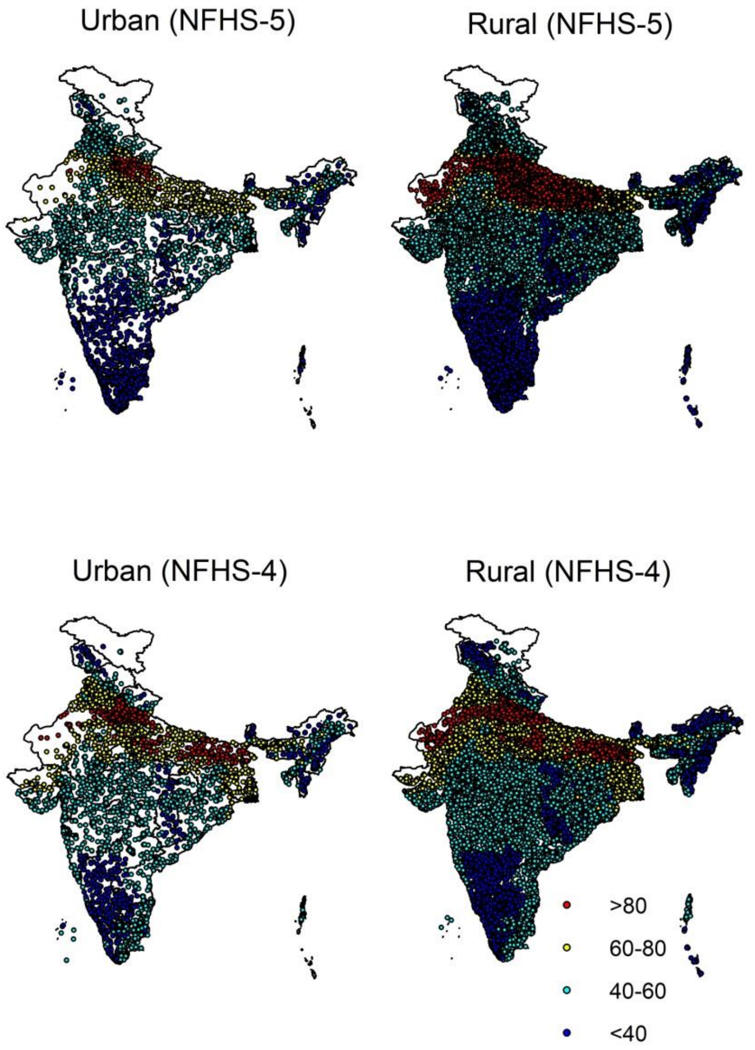

Figure S2. Ambient mean $PM_{2.5}$ concentration in the NFHS clusters. *Color codes* indicate the concentration ranges in µgm^{-3}. For the urban and rural clusters, a 2 and 5 km buffers were taken, respectively. We used the satellite-derived $PM_{2.5}$ at 1-km×1-km resolution (*Katoch et al., 2023*).

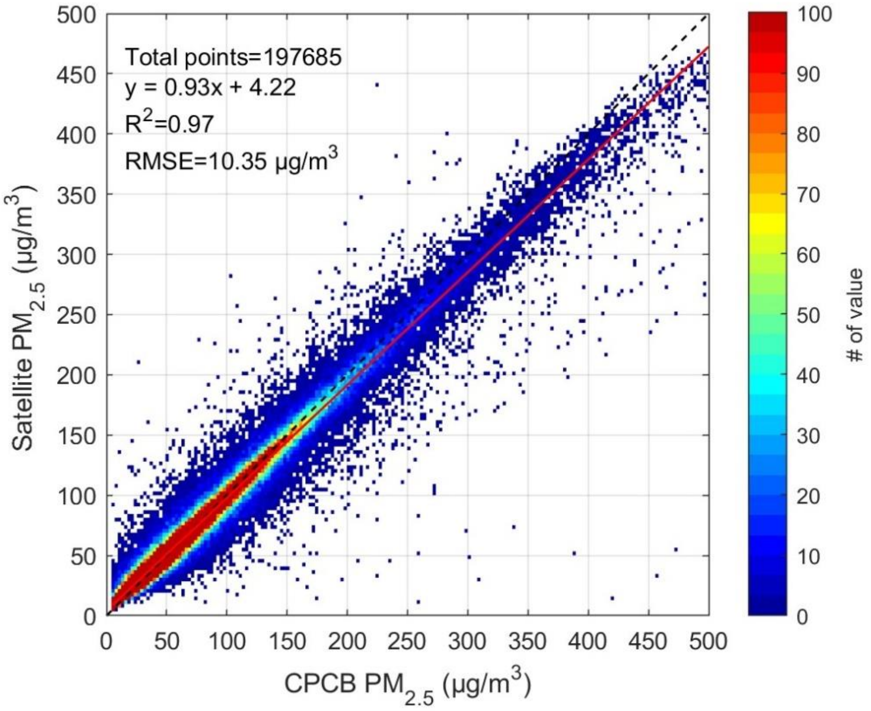

Figure S3. Regression statistics between daily satellite-derived $PM_{2.5}$ estimates with the ground-based observations across India from 2019 to 2021. For further details, please refer to our previous work (*Katoch et al.*, *2023*).

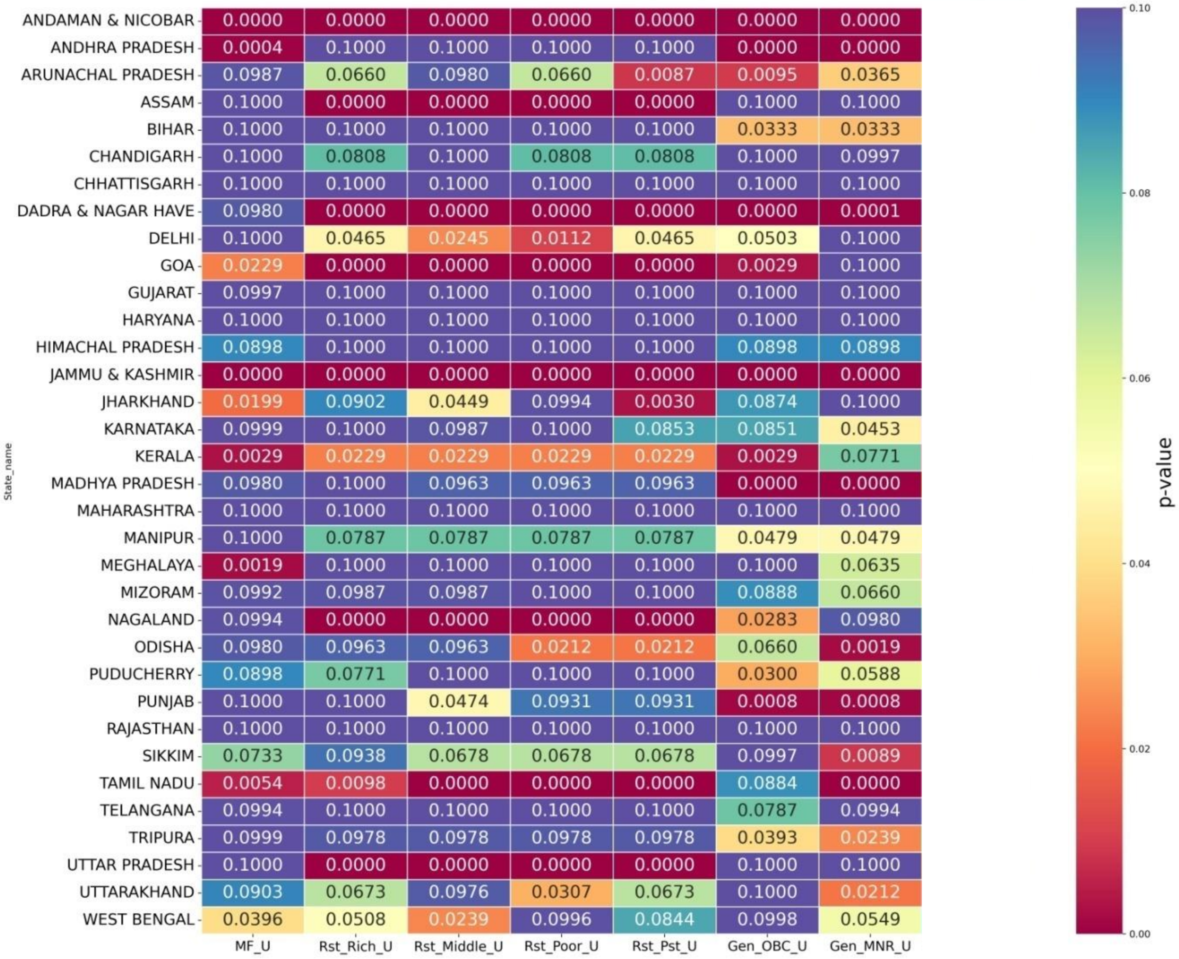

Figure S4.A. Estimated *p*-values of Z_{score} across the sub-populations in the urban regions of NFHS-5 (2019-21). The *color-codes* (*blue* to *red*) denote decreasing *p*-values and indicate greater significance level of associated to the mean Z_{score} estimate. The abbreviations '*MF*', '*Rst_Rich*', '*Rst_Middle*', '*Rst_Poor*', '*Rst_Pst*', '*Gen_OBC*', and '*Gen_MNR*' denote differences in estimated PWCs among Male-Female, Richest-Middle class, Richest-Poor, Richest-Poorest, General-OBC, and General-(SC+ST), respectively. The states are arranged in alphabetic order.

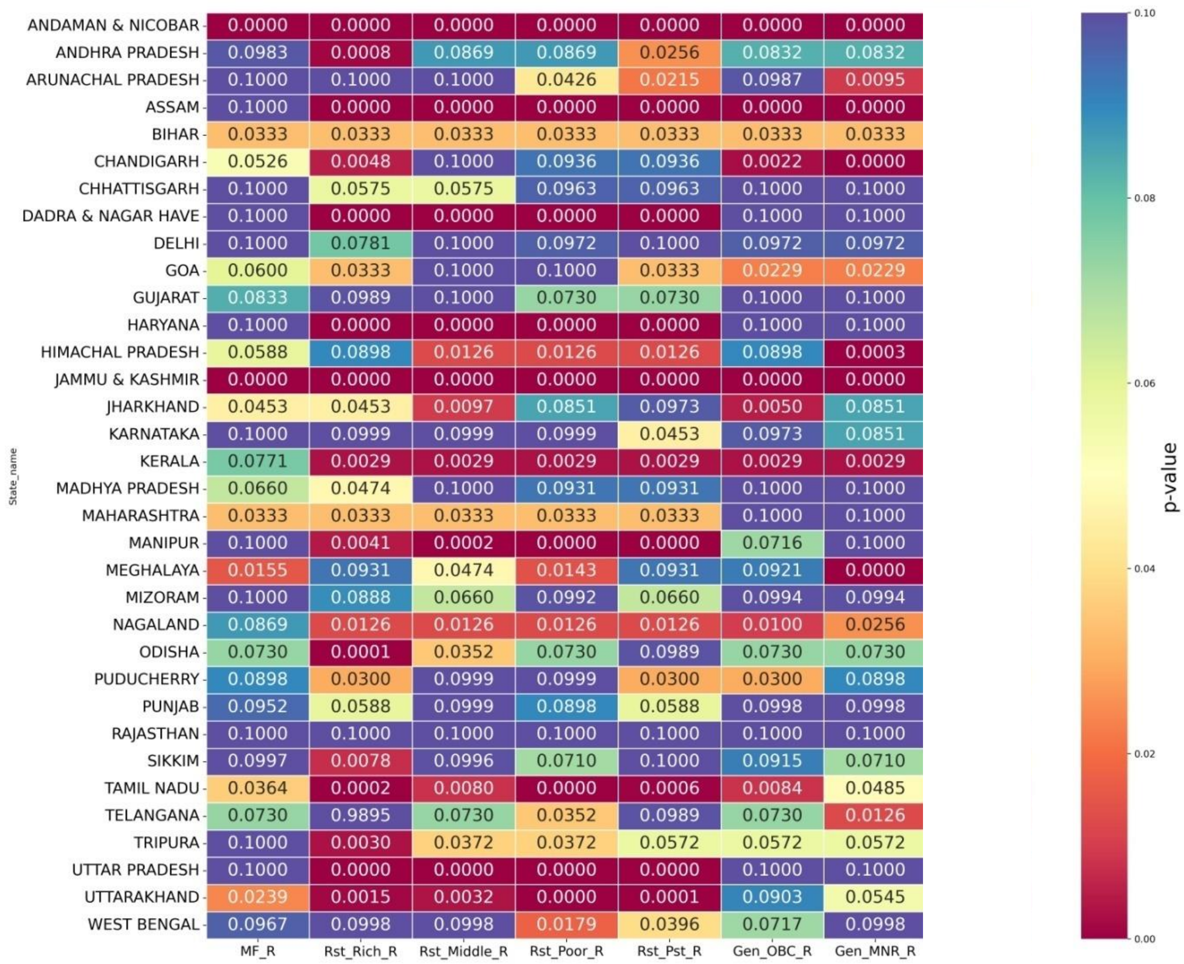

Figure S4.B: Estimated *p*-values of Z_{score} across the sub-populations in the rural regions of NFHS-5 (2019-21). The *color-codes* (*blue* to *red*) denote decreasing *p*-values and indicate greater significance level of associated to the mean Z_{score} estimate. The abbreviations '*MF*', '*Rst_Rich*', '*Rst_Middle*', '*Rst_Poor*', '*Rst_Pst*', '*Gen_OBC*', and '*Gen_MNR*' denote differences in estimated PWCs among Male-Female, Richest-Middle class, Richest-Poor, Richest-Poorest, General-OBC, and General-(SC+ST), respectively. The states are arranged in alphabetic order.

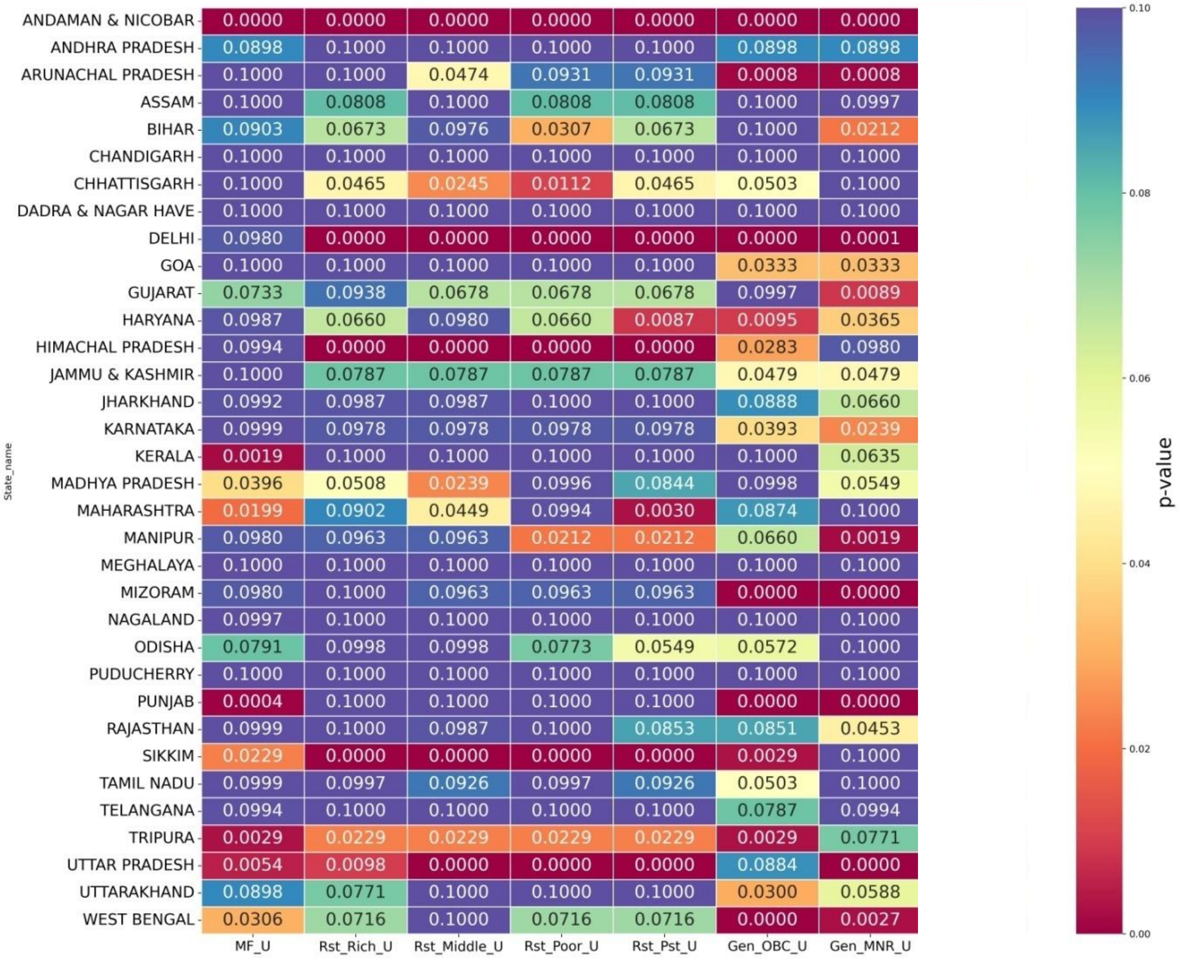

Figure S4.C: Estimated *p*-values of Z_{score} across the sub-populations in the urban regions of NFHS-4 (2015-16). The *color-codes* (*blue* to *red*) denote decreasing *p*-values and indicate greater significance level of associated to the mean Z_{score} estimate. The abbreviations '*MF*', '*Rst_Rich*', '*Rst_Middle*', '*Rst_Poor*', '*Rst_Pst*', '*Gen_OBC*', and '*Gen_MNR*' denote differences in estimated PWCs among Male-Female, Richest-Middle class, Richest-Poor, Richest-Poorest, General-OBC, and General-(SC+ST), respectively. The states are arranged in alphabetic order.

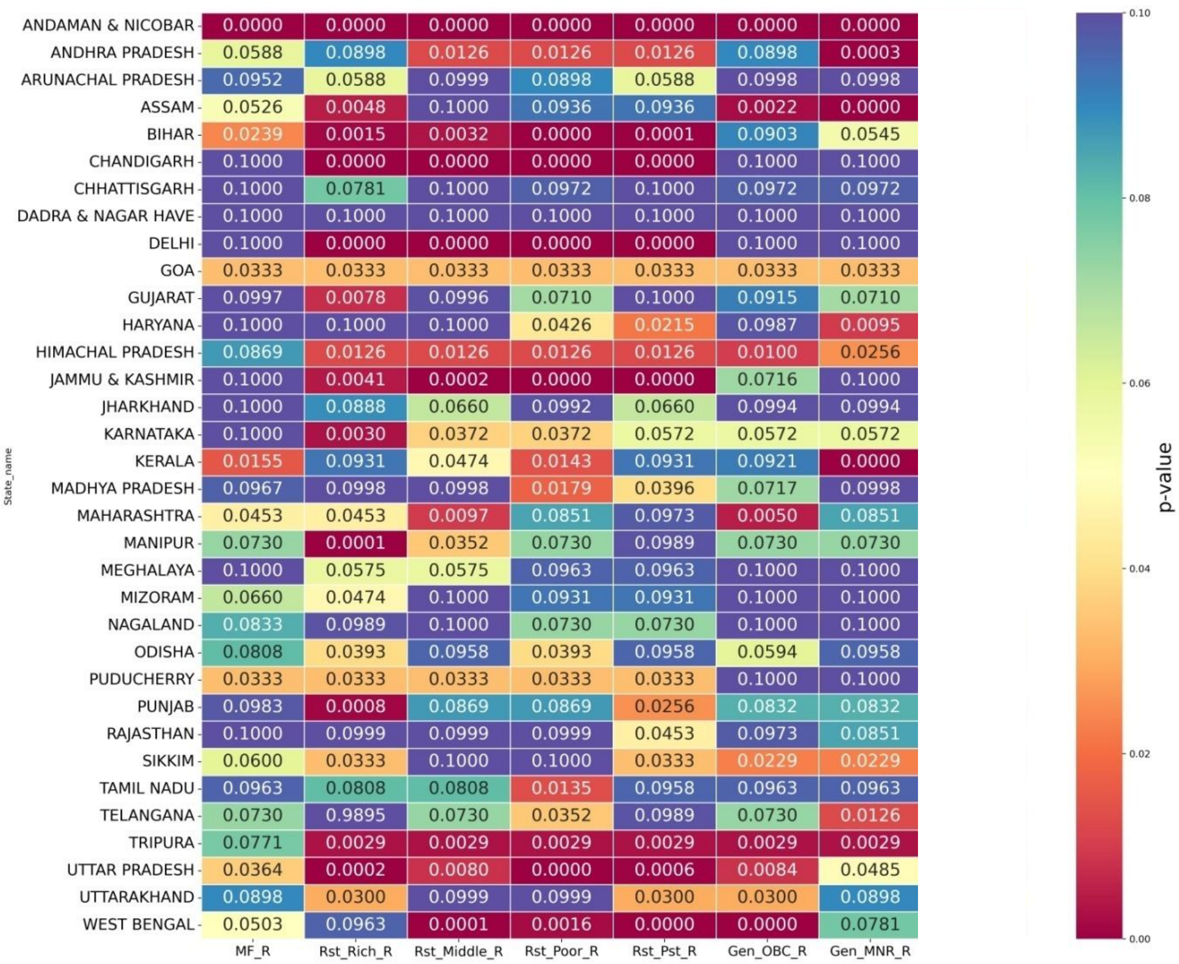

Figure S4.D: Estimated *p*-values of Z_{score} across the sub-populations in the rural regions of NFHS-4 (2015-16). The *color-codes* (*blue* to *red*) denote decreasing *p*-values and indicate greater significance level of associated to the mean Z_{score} estimate. The abbreviations '*MF*', '*Rst_Rich*', '*Rst_Middle*', '*Rst_Poor*', '*Rst_Pst*', '*Gen_OBC*', and '*Gen_MNR*' denote differences in estimated PWCs among Male-Female, Richest-Middle class, Richest-Poor, Richest-Poorest, General-OBC, and General-(SC+ST), respectively. The states are arranged in alphabetic order.

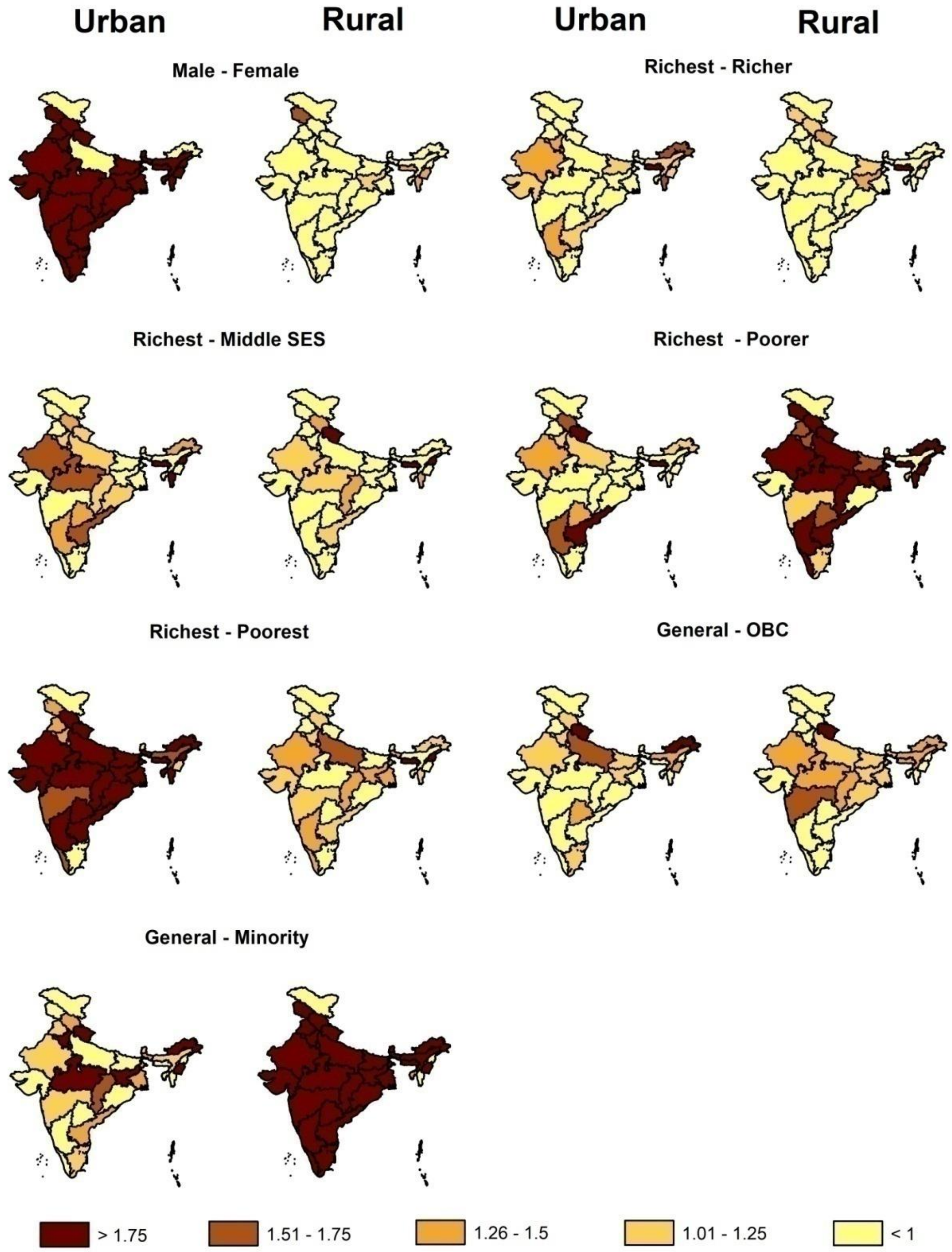

Figure S5.A: Estimated ratio (for NFHS-5) between the relative disparities (ratio of absolute values of Z_{score}) across the population subgroups at the state and their respective national levels. The *color-codes* indicating if the ratio is more than 1 (ratio>1), then the inequality increases if the scale of analysis transforms from national to subregional level. The Middle class and SC+ST subgroups are denoted here by "*Middle SES*" and *"Minority"*, respectively.

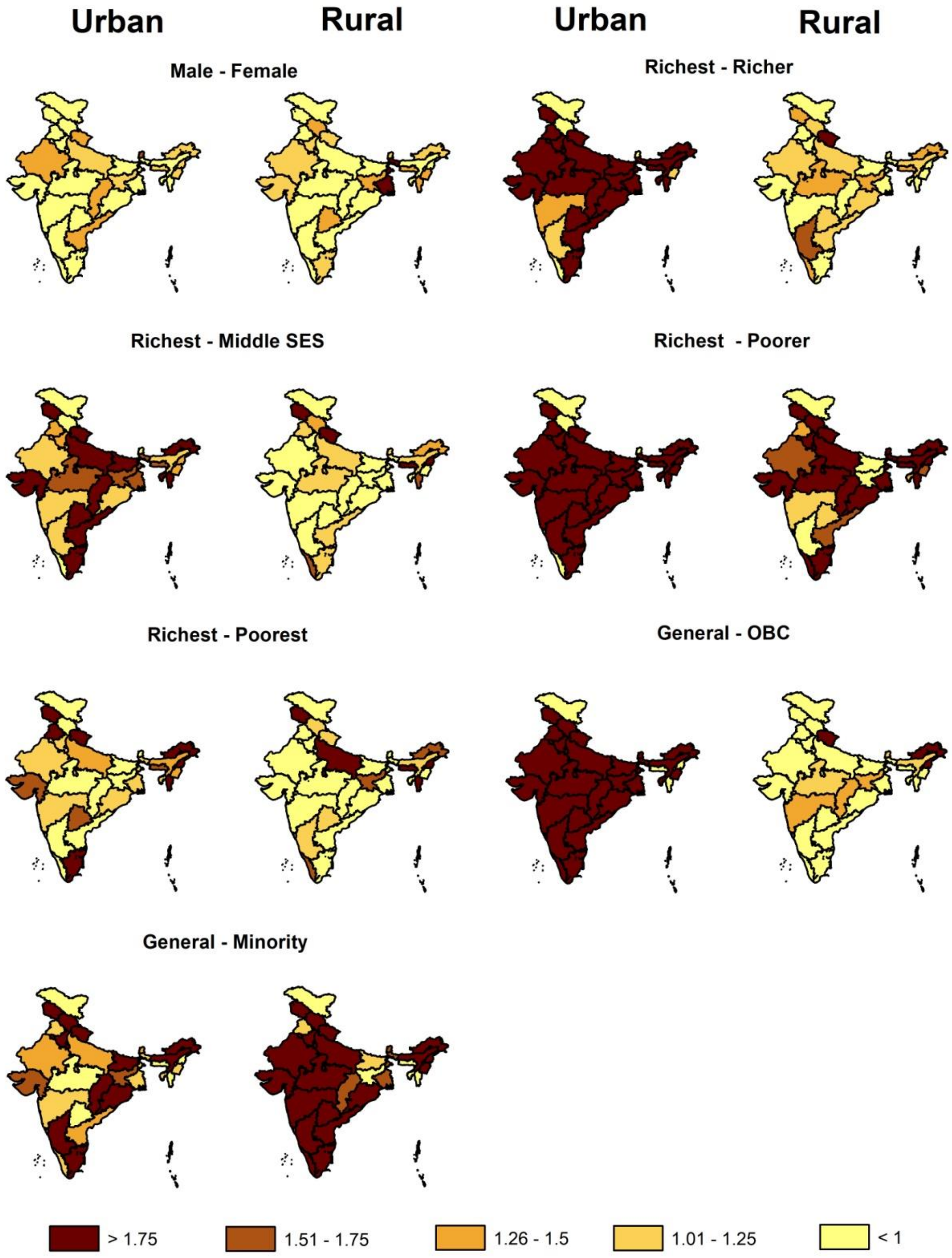

Figure S5.B: Estimated ratio (for NFHS-4) between the relative disparities (ratio of absolute values of Z_{score}) across the population subgroups at the state and their respective national levels. The *color-codes* indicating if the ratio is more than 1 (ratio>1), then the inequality increases if the scale of analysis transforms from national to subregional level. The Middle class and SC+ST subgroups are denoted here by "*Middle SES*" and *"Minority"*, respectively.

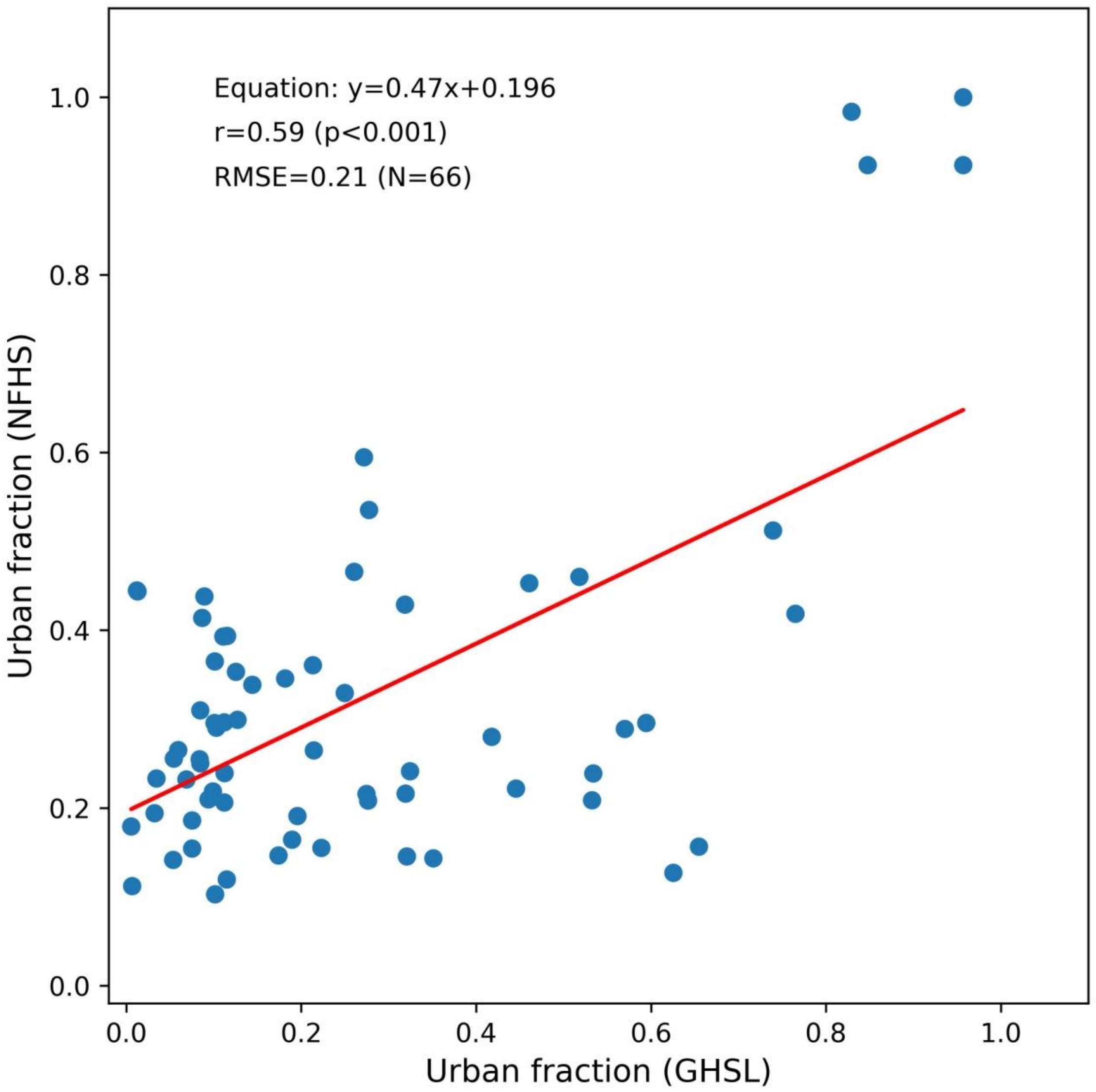

Figure S6: Estimated correlation statistics between state-level urban fractions derived using the GHSL and population-weighted NFHS datasets.

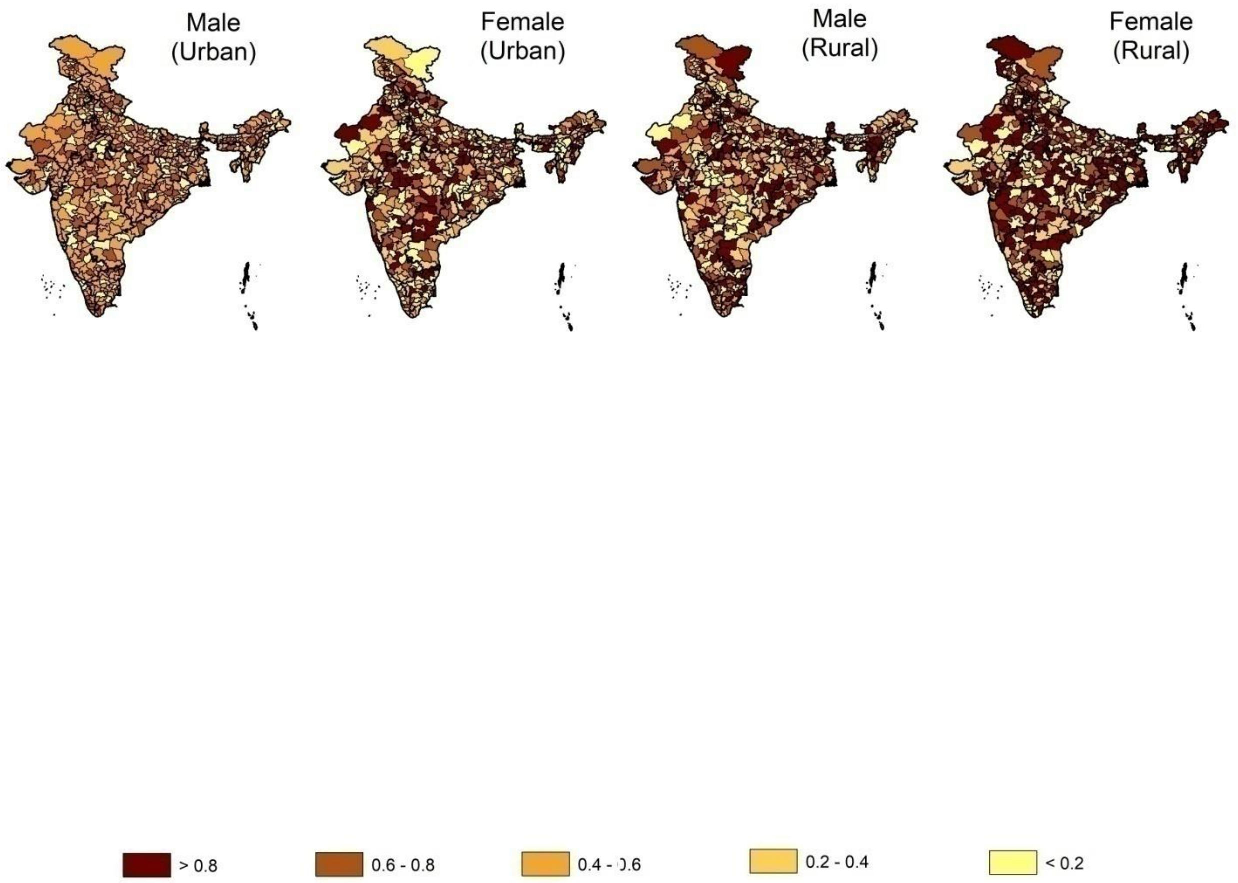

Figure S7.A: The sub-population fraction across the gender subgroups in urban and rural regions of NFHS-5. The *color-codes* denote the ranges of sub-population fraction with *yellow* to *brown* indicate increasing estimates. For every state, the cluster level demographic distribution has been analyzed.

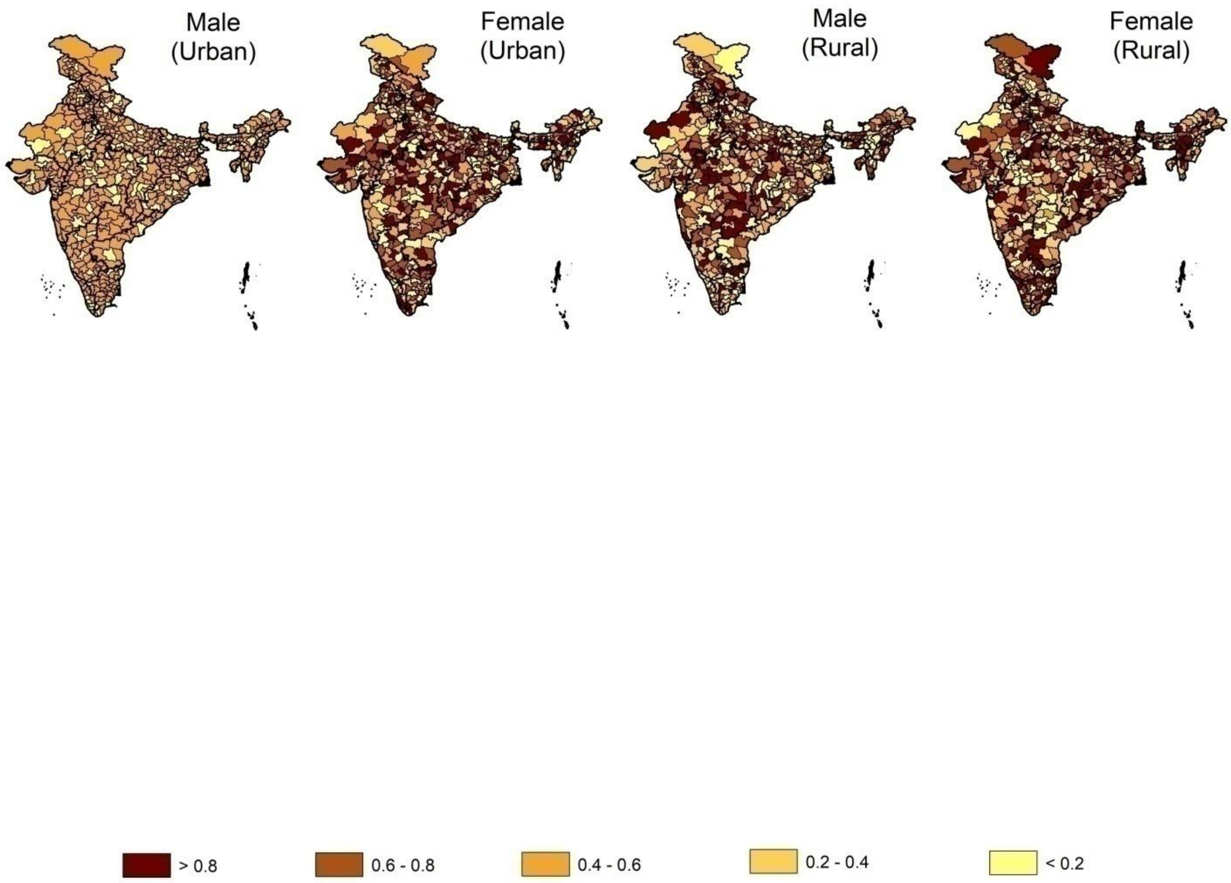

Figure S7.B: The sub-population fraction across the gender subgroups in urban and rural regions of NFHS-4. The *color-codes* denote the ranges of sub-population fraction with *yellow* to *brown* indicate increasing estimates. For every state, the cluster level demographic distribution has been analyzed.

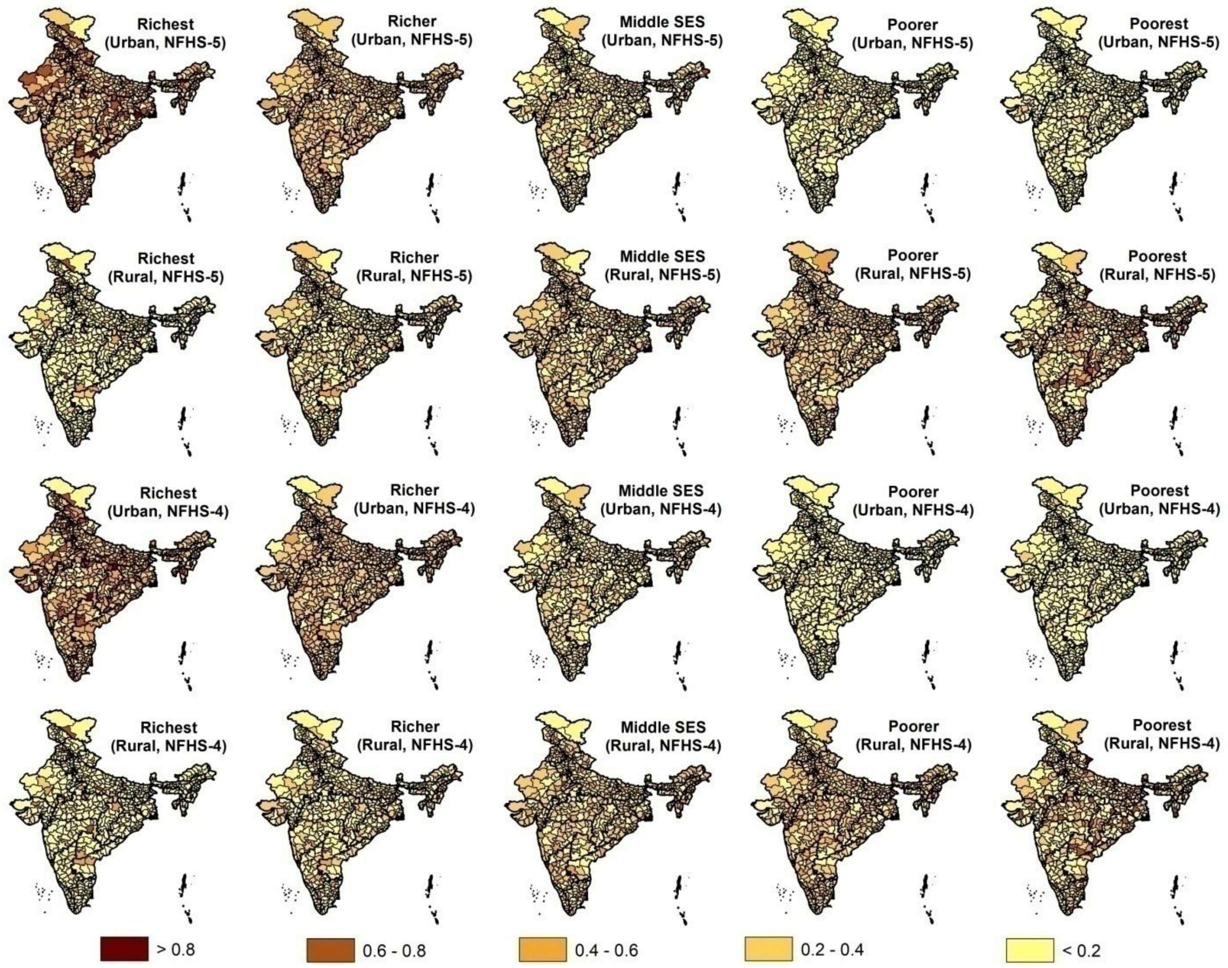

Figure S7.C: The sub-population fraction across the wealth subgroup categories in the urban and rural regions of NFHS rounds. The *color-codes* denote the ranges of sub-population fraction with *yellow* to *brown* indicate increasing estimates. For every state, the cluster level demographic distribution has been analyzed. The Middle class subgroup in denoted here by "*Middle SES*".

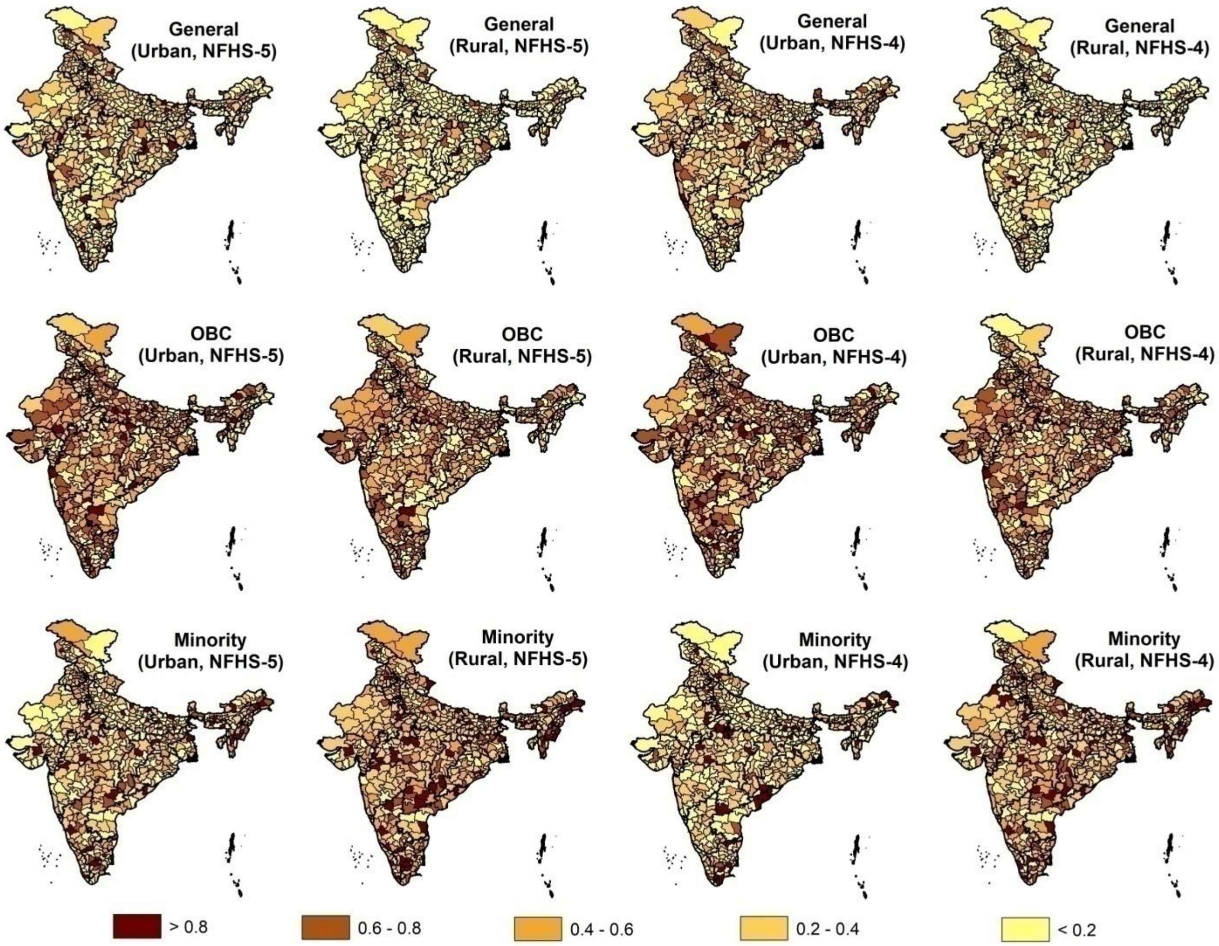

Figure S7.D: The sub-population fraction across the caste subgroup categories in the urban and rural regions of NFHS rounds. The *color-codes* denote the ranges of sub-population fraction with *yellow* to *brown* indicate increasing estimates. For every state, the cluster level demographic distribution has been analyzed. The SC+ST are denoted here by *"Minority"*.

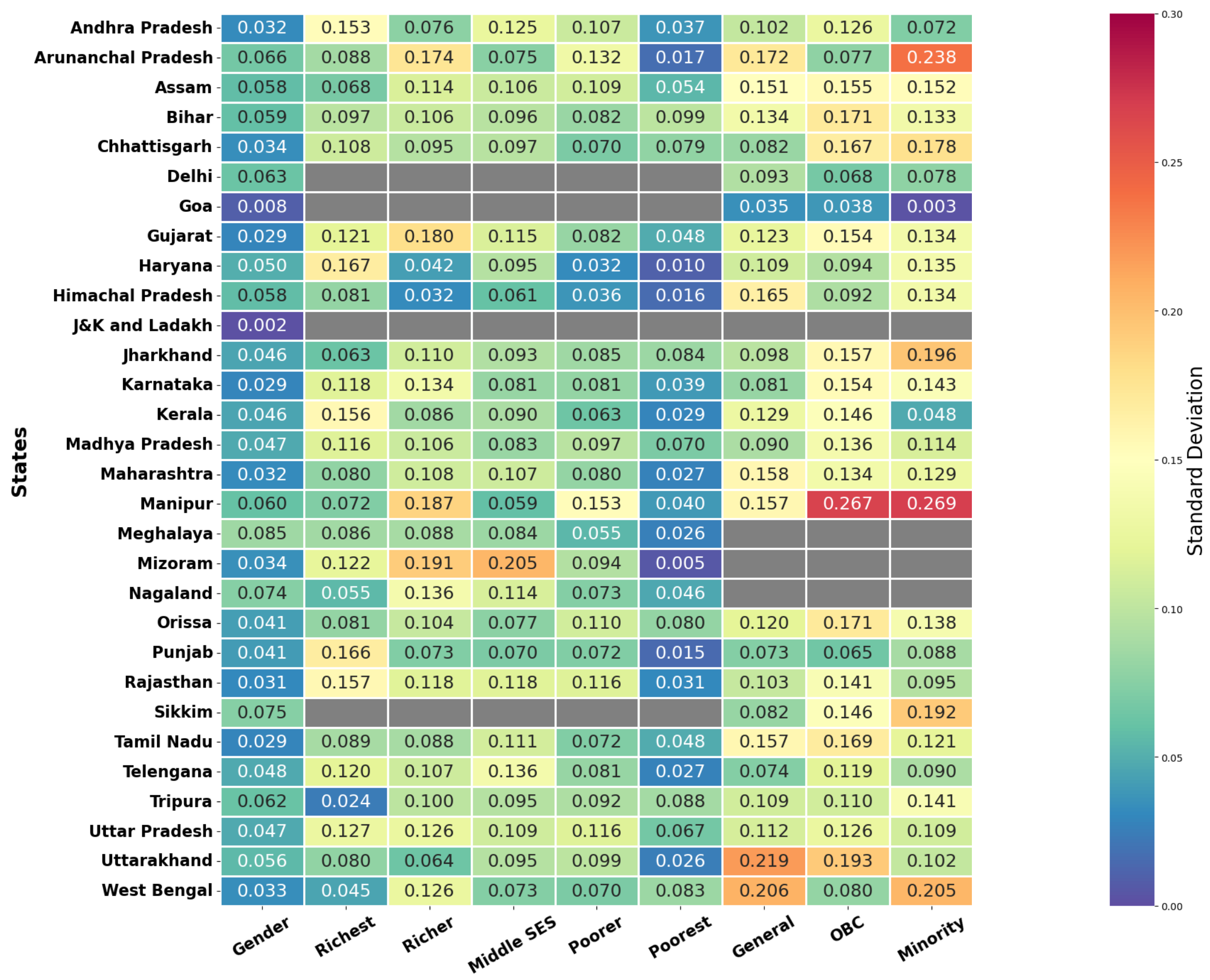

Figure S8.A: The estimated standard deviation of the sub-population distribution in urban regions of NFHS-5. The *color-codes* denote the ranges with *blue* to *red* indicate increasing estimates. For every state, the cluster level demographic distribution has been analyzed. The Middle class and SC+ST subgroups are denoted here by "*Middle SES*" and *"Minority"*, respectively.

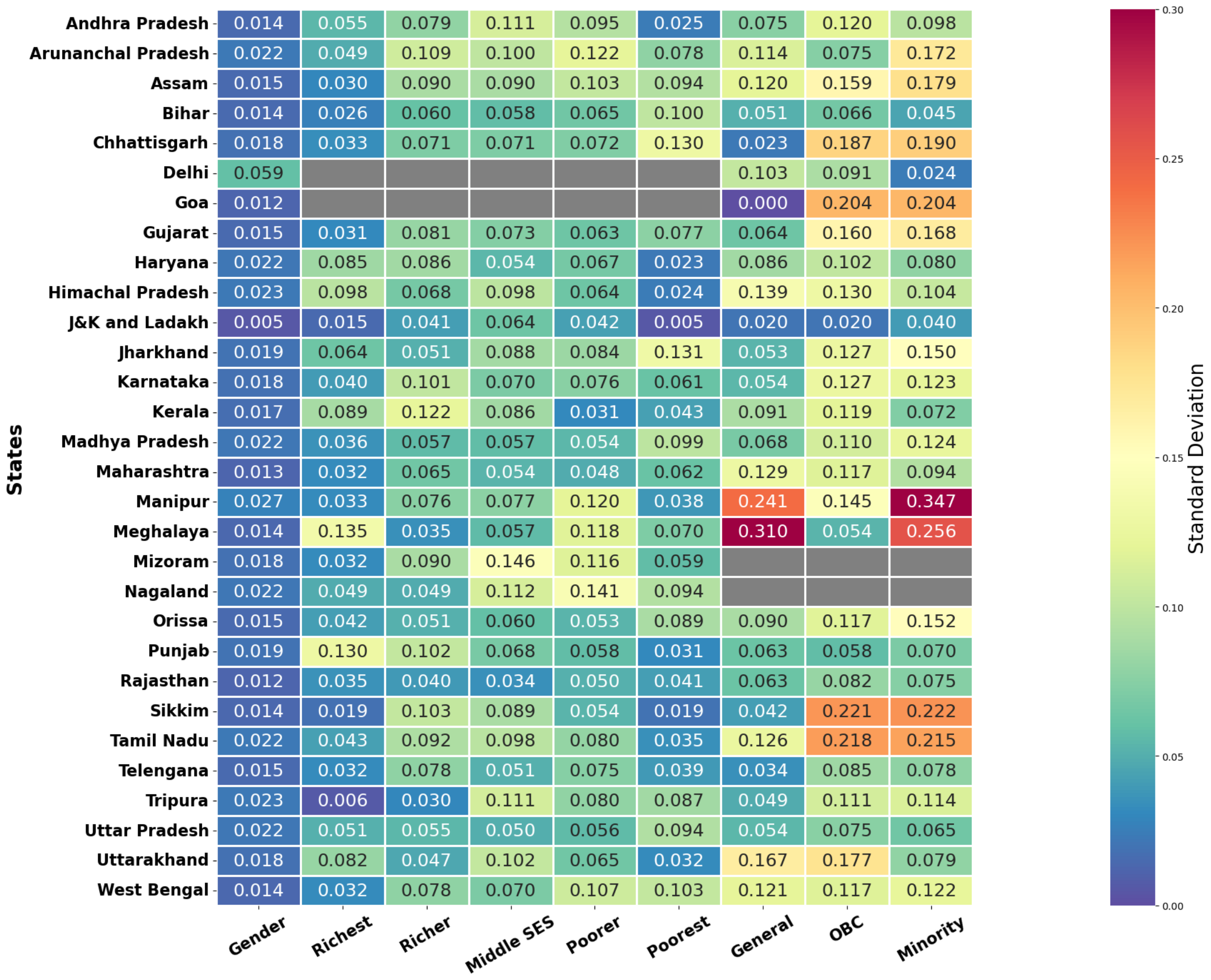

Figure S8.B: The estimated standard deviation of the sub-population distribution in rural regions of NFHS-5. The *color-codes* denote the ranges with *blue* to *red* indicate increasing estimates. For every state, the cluster level demographic distribution has been analyzed. The Middle class and SC+ST subgroups are denoted here by "*Middle SES*" and *"Minority"*, respectively.

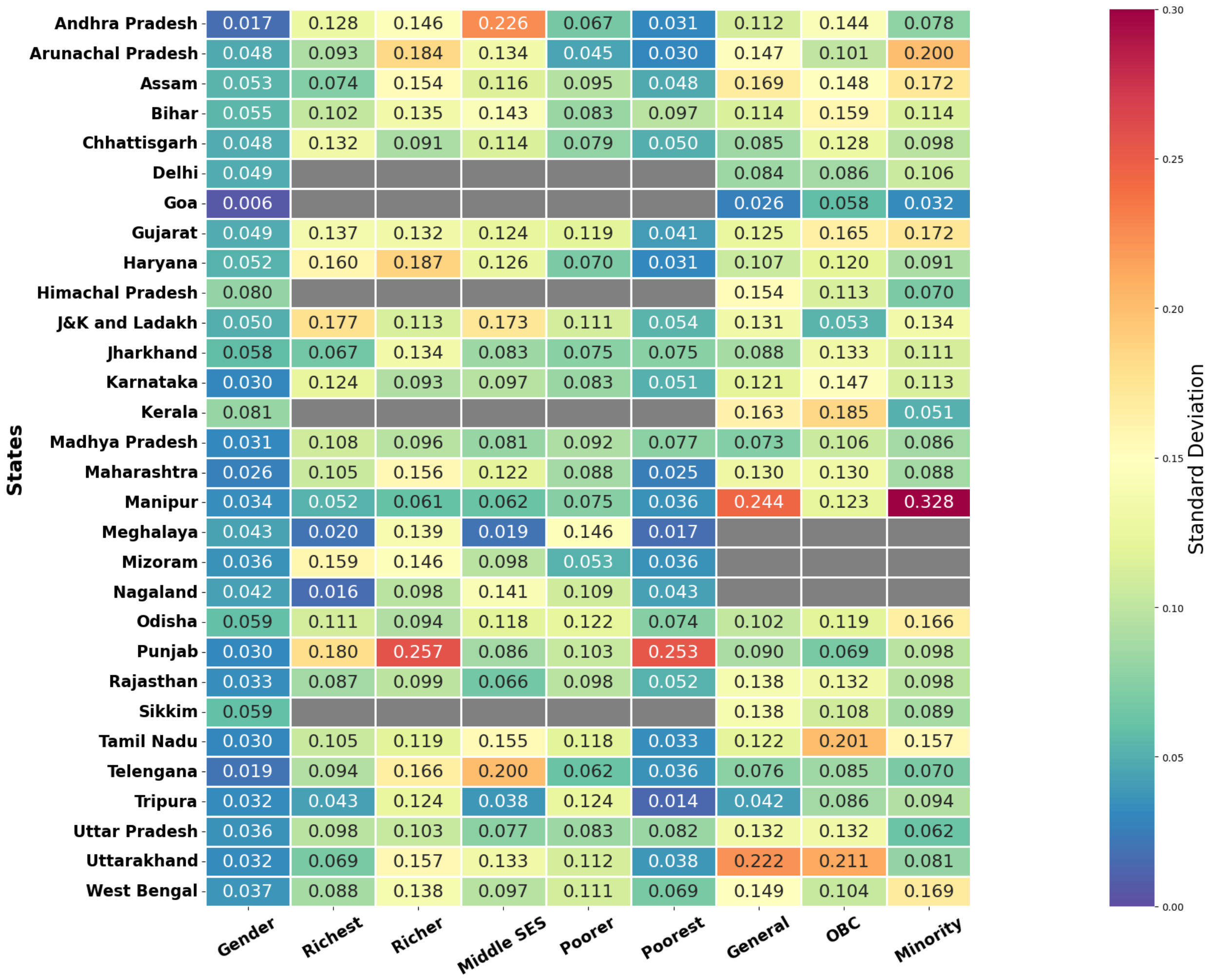

Figure S8.C: The estimated standard deviation of the sub-population distribution in urban regions of NFHS-4. The *color-codes* denote the ranges with *blue* to *red* indicate increasing estimates. For every state, the cluster level demographic distribution has been analyzed. The Middle class and SC+ST subgroups are denoted here by "*Middle SES*" and *"Minority"*, respectively.

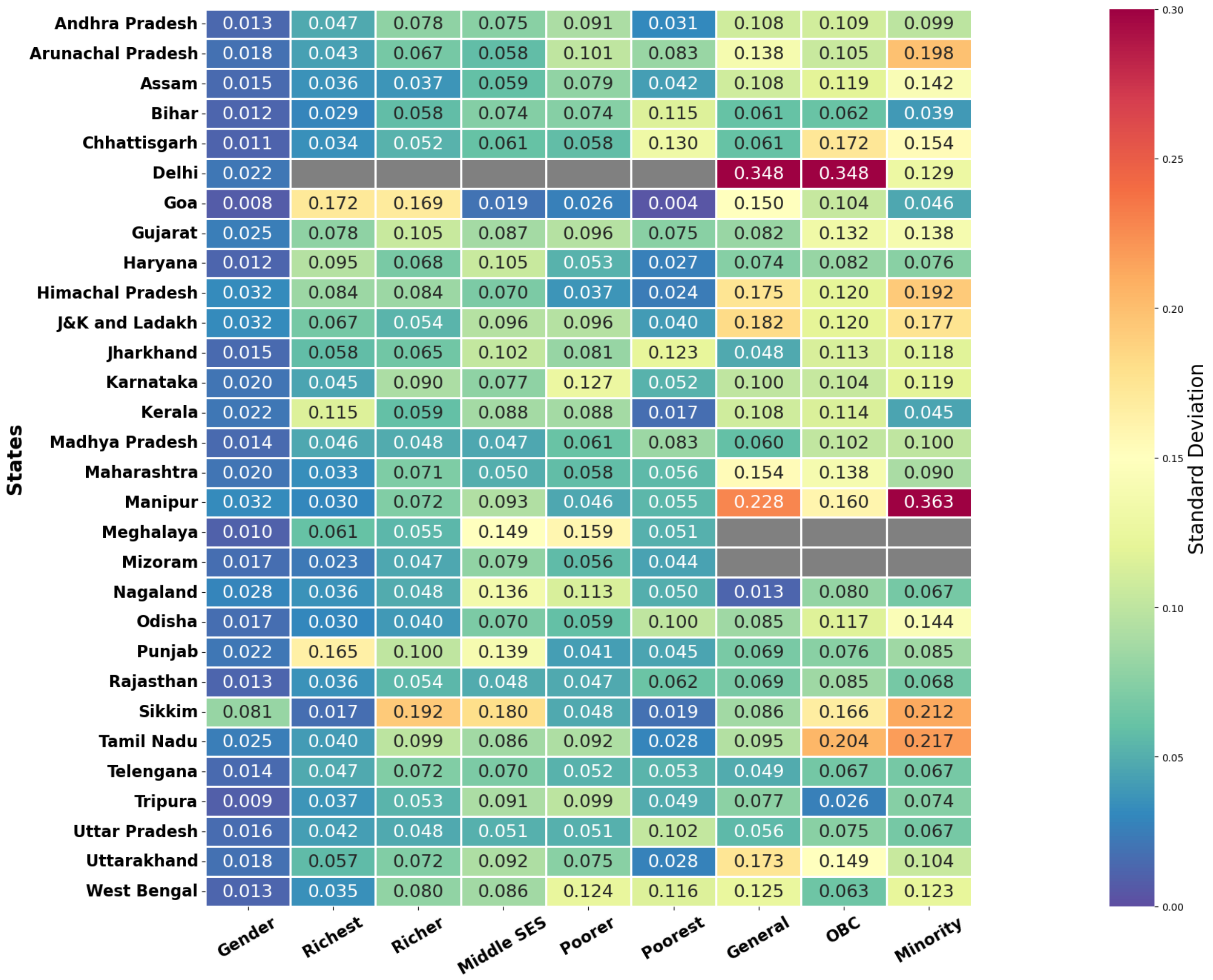

Figure S8.D: The estimated standard deviation of the sub-population distribution in rural regions of NFHS-4. The *color-codes* denote the ranges with *blue* to *red* indicate increasing estimates. For every state, the cluster level demographic distribution has been analyzed. The Middle class and SC+ST subgroups are denoted here by "*Middle SES*" and *"Minority"*, respectively.

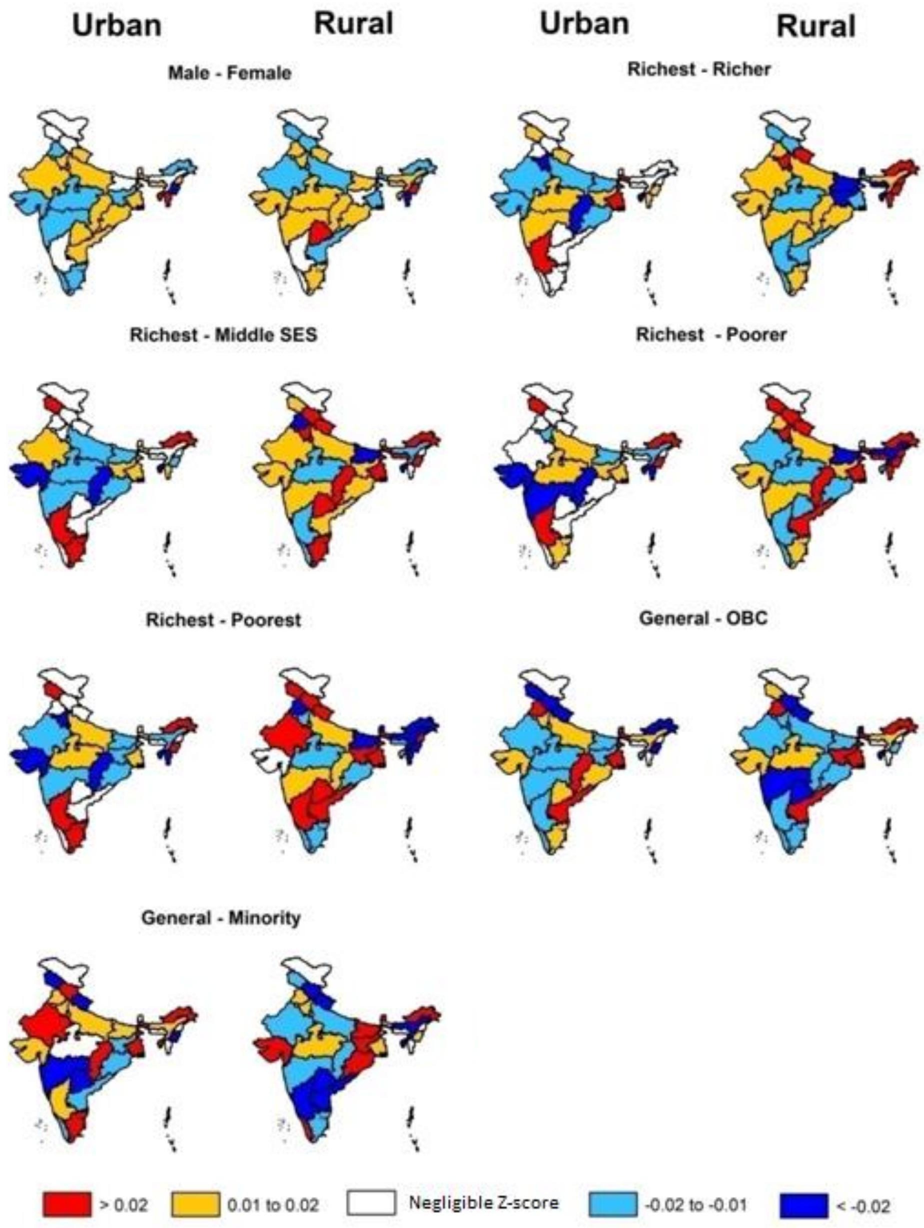

Figure S9: Estimated state-level relative disparity (denoted by Z_{score}) across the subgroups stratified by gender, wealth, and caste in the urban and rural regions across the states in NFHS-4. States highlighted with *colored*-background represent regions where the mean Z_{score} estimates (standardized mean differences in PWCs between sub-populations) indicate statistically significant disparity (p<0.1), rejecting the *null hypothesis* of equal or comparable PWCs at the 90% confidence level. For better interpretation, we considered a mean Z_{score} difference within ±0.01 standard deviations between the estimated PWCs of two subgroups as negligible, and accordingly, these states were highlighted with a *white*-background. The Middle class and SC+ST subgroups are denoted here by "*Middle SES*" and *"Minority"*, respectively.

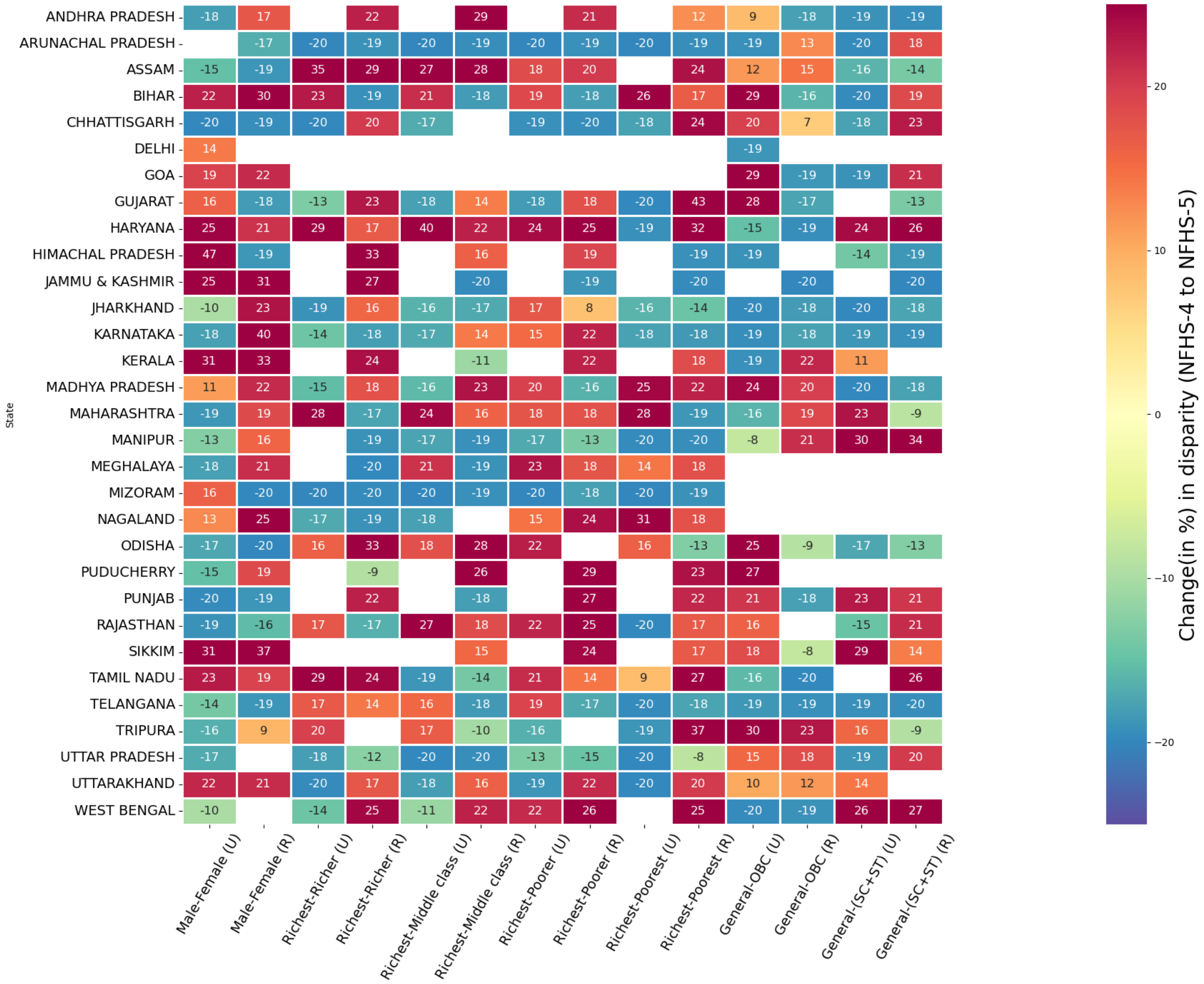

Figure S10: Changes (in-%, absolute values of Z_{score}) in relative disparity in ambient $PM_{2.5}$ concentration among the subgroups from NFHS-4 (2015-16) to NFHS-5 (2019-21) for urban (U) and rural (R) regions across the states. The states denoted by *shades of red* color indicate that relative disparity increased between the two time points, whereas the *shades of blue* indicate reduction in relative disparity. The states in *blank-boxes* depict insignificant change in relative disparity over the time points.

Table S1.A: Estimated mean of subgroup-weighted $PM_{2.5}$ exposure (PWC, in μgm^{-3}) (Coefficient of Variation, CV) and the Z_{score} (95% confidence intervals, CIs) among the population subgroups in urban and rural regions of India. To estimate relative disparity across the sub-populations, we considered male, richest, and general as the reference-subgroups (Ref.). For instance, a positive Z_{score} denotes that the estimated PWCs among male, richest, and general are higher as compared to their demographic counterparts; and the negative values denote the opposite. For this sensitivity assessment, we considered 2 km buffer for both urban and rural clusters.

2 km buffer for urban and rural clusters	NFHS-5 (2019-21)				NFHS-4 (2015-16)			
	Urban		Rural		Urban		Rural	
	Mean (CV)	Z_{score} (95% CI)	Mean (CV)	Z_{score} (95% CI)	Mean (CV)	Z_{score} (95% CI)	Mean (CV)	Z_{score} (95% CI)
**Male**	50.8 (0.347)	Ref.	53.8 (0.282)	Ref.	55.7 (0.351)	Ref.	59.2 (0.303)	Ref.
**Female**	50.6 (0.34)	0.0033 (0.0032-0.0034)	54.8 (0.28)	-0.0215 (-0.0224 – -0.0206)	56.5 (0.361)	-0.01 (-0.0104 – -0.0096)	60.7 (0.301)	-0.0229 (-0.0238 – -0.022)
**Richest**	51.1 (0.385)	Ref.	54.3 (0.298)	Ref.	54.3 (0.39)	Ref.	58.3 (0.315)	Ref.
**Richer**	51.6 (0.337)	-0.0509 (-0.0532 – -0.0486)	50.6 (0.311)	0.0725 (0.0697 – 0.0753)	52.6 (0.392)	0.0195 (0.0187 – 0.0203)	55.7 (0.33)	0.0385 (0.037 – 0.04)
**Middle class**	54.6 (0.306)	-0.0526 (-0.0547 – -0.0505)	51.6 (0.304)	0.0532 (0.0509 – 0.0555)	52.9 (0.384)	0.0163 (0.0157 – 0.0169)	56.7 (0.326)	0.0236 (0.0227 – 0.0245)
**Poorer**	56.7 (0.281)	-0.0875 (-0.0904 – -0.0846)	55.7 (0.272)	-0.0285 (-0.0295 – -0.0275)	55 (0.351)	-0.0085 (-0.0089 – -0.0081)	60.8 (0.296)	-0.0378 (-0.0392 – -0.0364)
**Poorest**	58.5 (0.255)	-0.1215 (-0.1259 – -0.1171)	57.9 (0.243)	-0.0783 (-0.0811 – -0.0755)	60.5 (0.308)	-0.078 (-0.0081 – -0.0075)	65.8 (0.266)	-0.1163 (-0.1211 – -0.1115)
**General**	54.2 (0.328)	Ref.	55.8 (0.273)	Ref.	58.9 (0.335)	Ref.	61.1 (0.292)	Ref.
**OBC**	49 (0.362)	0.0823 (0.0786-0.086)	55.7 (0.295)	0.002 (0.0019 - 0.0021)	55.5 (0.367)	0.0423 (0.0405 – 0.0441)	62.3 (0.293)	-0.0184 (-0.019 – -0.0178)
**SC+ST**	50.6 (0.331)	0.0602 (0.0578-0.0626)	53.1 (0.265)	0.0629 (0.0604 – 0.0654)	55.6 (0.356)	0.0421 (0.0406 – 0.0436)	58.1 (0.297)	0.0487 (0.0463 – 0.0511)

Table S1.B: Estimated mean of subgroup-weighted $PM_{2.5}$ exposure (PWC, in μgm^{-3}) (Coefficient of Variation, CV) and the Z_{score} (95% confidence intervals, CIs) among the population subgroups in urban and rural regions of India. To estimate relative disparity across the sub-populations, we considered male, richest, and general as the reference-subgroups (Ref.). For instance, a positive Z_{score} denotes that the estimated PWCs among male, richest, and general are higher as compared to their demographic counterparts; and the negative values denote the opposite. For this sensitivity assessment, we considered 5 km buffer for both urban and rural clusters.

**5 km buffer for urban and rural clusters**	**NFHS-5 (2019-21)**				**NFHS-4 (2015-16)**			
	**Urban**		**Rural**		**Urban**		**Rural**	
	**Mean (CV)**	**Z_{score} (95% CI)**	**Mean (CV)**	**Z_{score} (95% CI)**	**Mean (CV)**	**Z_{score} (95% CI)**	**Mean (CV)**	**Z_{score} (95% CI)**
**Male**	45.4 (0.371)	Ref.	52.4 (0.298)	Ref.	49 (0.378)	Ref.	57.9 (0.307)	Ref.
**Female**	44.8 (0.364)	0.0109 (0.105 – 0.113)	53.4 (0.286)	-0.0216 (-0.0226 – -0.0206)	48.8 (0.394)	0.0028 (0.0027 – 0.0029)	59.2 (0.305)	-0.0203 (-0.0211 – -0.0195)
**Richest**	43.9 (0.4)	Ref.	52.3 (0.307)	Ref.	47.7 (0.4)	Ref.	56.3 (0.319)	Ref.
**Richer**	44.3 (0.369)	-0.0069 (-0.0071 – -0.0067)	49.1 (0.32)	0.0634 (0.0611 – 0.0657)	47.1 (0.41)	0.0081 (0.0078 – 0.0084)	54.2 (0.335)	0.0322 (0.0306 – 0.0338)
**Middle class**	45.7 (0.348)	-0.032 (-0.0332 – -0.0308)	50.1 (0.312)	0.0438 (0.042 – 0.0456)	52.7 (0.365)	-0.0681 (-0.071 – -0.0652)	55.1 (0.33)	0.0183 (0.0177 – 0.0189)
**Poorer**	47.3 (0.326)	-0.0623 (-0.0644 – -0.0602)	53.9 (0.281)	-0.0328 (-0.0343 – -0.0313)	54.1 (0.341)	-0.0908 (-0.095 – -0.0866)	59.2 (0.3)	-0.0454 (-0.0471 – -0.0437)
**Poorest**	48.1 (0.309)	-0.0793 (-0.0834 – -0.0758)	56 (0.251)	-0.0812 (-0.0844 – -0.078)	56 (0.323)	-0.1199 (-0.1252 – -0.1146)	64.2 (0.272)	-0.126 (-0.131 – -0.12)
**General**	46.9 (0.358)	Ref.	54.3 (0.279)	Ref.	54.8 (0.33)	Ref.	59.6 (0.296)	Ref.
**OBC**	44.5 (0.386)	0.0417 (0.0398 – 0.0436)	54.3 (0.302)	---	53.8 (0.361)	-0.1199 (-0.1237 – -0.1161)	60.8 (0.298)	-0.0188 (-0.0196 – -0.18)
**SC+ST**	46.2 (0.347)	0.013 (0.0125 – 0.0135)	51.9 (0.27)	0.0564 (0.0541 – 0.0587)	54.7 (0.345)	0.0003 (0.0001 – 0.0002)	57 (0.299)	0.0432 (0.0417 – 0.0447)

Table S2.A: Estimated mean PWC (in μgm^{-3}) (Coefficient of Variation, CV) of ambient $PM_{2.5}$ across the population subgroups in urban and rural regions of the states with high socio-demographic index (SDI) of NFHS-5 (2019-21). We followed the conventional NFHS framework (2 km buffer for urban and 5 km buffer for rural clusters) and performed two sensitivity assessments as well. In the 1st sensitivity assessment, we considered 2 km buffer for both urban and rural clusters; and in the 2nd sensitivity assessment, we considered 5 km buffer for both urban and rural clusters, respectively.

	NFHS framework		Sensitivity assessment 1		Sensitivity assessment 2	
	2 km urban-buffer and 5 km rural-buffer		2 km buffer for urban and rural clusters		5 km buffer for urban and rural clusters	
	Urban	Rural	Urban	Rural	Urban	Rural
Male	43 (0.417)	40.5 (0.231)	41.9 (0.42)	40.9 (0.229)	36.1 (0.428)	39.5 (0.238)
Female	42.3 (0.432)	40.4 (0.237)	41.8 (0.436)	40.4 (0.238)	36.3 (0.437)	39.5 (0.238)
Richest	31.4 (0.639)	42.8 (0.271)	36 (0.629)	43.4 (0.27)	26.4 (0.668)	41.6 (0.275)
Richer	31.6 (0.506)	39.4 (0.247)	34.2 (0.51)	39.6 (0.247)	28.4 (0.5)	38.4 (0.251)
Middle class	31.4 (0.475)	38.6 (0.224)	33 (0.495)	38.8 (0.223)	28.5 (0.449)	37.8 (0.225)
Poorer	34.5 (0.431)	39.5 (0.2)	35.8 (0.448)	39.8 (0.2)	31.9 (0.426)	38.9 (0.201)
Poorest	33.4 (0.326)	40.6 (0.178)	34.7 (0.324)	40.7 (0.179)	30.7 (0.337)	40.2 (0.178)
General	48 (0.432)	42.3 (0.214)	47.4 (0.437)	42.3 (0.214)	39.8 (0.45)	41.4 (0.213)
OBC	37.7 (0.372)	38.4 (0.258)	37.3 (0.371)	38.3 (0.259)	33.2 (0.38)	37.4 (0.264)
SC+ST	44.2 (0.402)	41.4 (0.221)	43.2 (0.407)	41.4 (0.222)	38.5 (0.411)	40.3 (0.224)

Table S2.B: Estimated mean PWC (in μgm^{-3}) (Coefficient of Variation, CV) of ambient $PM_{2.5}$ across the population subgroups in urban and rural regions of the states with high socio-demographic index (SDI) of NFHS-4 (2015-16). We followed the conventional NFHS framework (2 km buffer for urban and 5 km buffer for rural clusters) and performed two sensitivity assessments as well. In the 1st sensitivity assessment, we considered 2 km buffer for both urban and rural clusters; and in the 2nd sensitivity assessment, we considered 5 km buffer for both urban and rural clusters, respectively.

	NFHS framework		Sensitivity assessment 1		Sensitivity assessment 2	
	2 km urban-buffer and 5 km rural-buffer		2 km buffer for urban and rural clusters		5 km buffer for urban and rural clusters	
	Urban	Rural	Urban	Rural	Urban	Rural
Male	45.5 (0.361)	45.2 (0.258)	46.7 (0.354)	45.4 (0.258)	39.9 (0.373)	44.5 (0.257)
Female	47.7 (0.395)	45.6 (0.259)	48.8 (0.395)	45.7 (0.259)	40.4 (0.425)	44.7 (0.258)
Richest	40.1 (0.509)	47.9 (0.264)	44.1 (0.5)	48.1 (0.263)	32.5 (0.556)	46.4 (0.27)
Richer	38 (0.439)	44.5 (0.268)	41.7 (0.441)	44.8 (0.267)	32.7 (0.471)	43.5 (0.268)
Middle class	39.5 (0.434)	44 (0.258)	42.5 (0.44)	44.3 (0.258)	33.3 (0.473)	43.1 (0.254)
Poorer	39.4 (0.398)	44.9 (0.238)	41.9 (0.42)	45.1 (0.238)	34.9 (0.425)	44.4 (0.232)
Poorest	41 (0.3)	45.6 (0.199)	41.9 (0.317)	45.6 (0.2)	38.7 (0.299)	45.1 (0.192)
General	49.3 (0.394)	46.4 (0.236)	51 (0.39)	46.5 (0.235)	42.3 (0.401)	45.5 (0.238)
OBC	44.4 (0.368)	44.1 (0.25)	45.4 (0.366)	44.2 (0.25)	38.6 (0.389)	43.3 (0.248)
SC+ST	50.4 (0.378)	46.8 (0.268)	50.9 (0.381)	46.9 (0.268)	41.5 (0.431)	45.9 (0.267)

Table S2.C: Estimated mean PWC (in μgm^{-3}) (Coefficient of Variation, CV) of ambient $PM_{2.5}$ across the population subgroups in urban and rural regions of the states with middle socio-demographic index (SDI) of NFHS-5 (2019-21). We followed the conventional NFHS framework (2 km buffer for urban and 5 km buffer for rural clusters) and performed two sensitivity assessments as well. In the 1st sensitivity assessment, we considered 2 km buffer for both urban and rural clusters; and in the 2nd sensitivity assessment, we considered 5 km buffer for both urban and rural clusters, respectively.

	NFHS framework		Sensitivity assessment 1		Sensitivity assessment 2	
	2 km urban-buffer and 5 km rural-buffer		2 km buffer for urban and rural clusters		5 km buffer for urban and rural clusters	
	Urban	Rural	Urban	Rural	Urban	Rural
**Male**	47.2 (0.304)	46.6 (0.268)	45.7 (0.319)	46.4 (0.269)	41.2 (0.332)	45.6 (0.274)
**Female**	46.3 (0.304)	46.6 (0.266)	45.3 (0.316)	46.5 (0.267)	40.8 (0.338)	45.7 (0.271)
**Richest**	35.7 (0.427)	51.1 (0.31)	37.8 (0.413)	51.4 (0.309)	31.4 (0.475)	49.7 (0.32)
**Richer**	36.4 (0.361)	44.2 (0.302)	38.1 (0.35)	44.3 (0.301)	32.5 (0.389)	43.4 (0.308)
**Middle class**	39.4 (0.335)	42.9 (0.27)	40.6 (0.325)	43.1 (0.269)	35 (0.363)	42.2 (0.274)
**Poorer**	43.8 (0.291)	46.4 (0.235)	44.3 (0.292)	46.5 (0.234)	39.2 (0.319)	45.5 (0.24)
**Poorest**	46.5 (0.251)	51.2 (0.205)	47.4 (0.249)	51.3 (0.204)	41.7 (0.276)	50.2 (0.208)
**General**	51.5 (0.286)	50.7 (0.265)	50.2 (0.298)	50.5 (0.267)	43.8 (0.331)	49.4 (0.273)
**OBC**	41.7 (0.318)	42.8 (0.291)	40.5 (0.332)	42.7 (0.291)	37 (0.345)	42.2 (0.294)
**SC+ST**	46.9 (0.301)	47.1 (0.25)	46.1 (0.311)	47 (0.251)	42.6 (0.322)	46 (0.254)

Table S2.D: Estimated mean PWC (in μgm^{-3}) (Coefficient of Variation, CV) of ambient $PM_{2.5}$ across the population subgroups in urban and rural regions of the states with middle socio-demographic index (SDI) of NFHS-4 (2015-16). We followed the conventional NFHS framework (2 km buffer for urban and 5 km buffer for rural clusters) and performed two sensitivity assessments as well. In the 1st sensitivity assessment, we considered 2 km buffer for both urban and rural clusters; and in the 2nd sensitivity assessment, we considered 5 km buffer for both urban and rural clusters, respectively.

	NFHS framework		Sensitivity assessment 1		Sensitivity assessment 2	
	2 km urban-buffer and 5 km rural-buffer		2 km buffer for urban and rural clusters		5 km buffer for urban and rural clusters	
	Urban	Rural	Urban	Rural	Urban	Rural
**Male**	52.4 (0.327)	54.3 (0.308)	47.9 (0.351)	53.6 (0.311)	47.9 (0.351)	53.6 (0.311)
**Female**	52.2 (0.355)	53.9 (0.319)	46.8 (0.391)	53.4 (0.322)	46.8 (0.391)	53.4 (0.322)
**Richest**	43.4 (0.437)	60.6 (0.329)	38.2 (0.491)	59.4 (0.332)	38.2 (0.491)	59.4 (0.332)
**Richer**	43.8 (0.405)	52.3 (0.341)	37.7 (0.469)	51.5 (0.346)	37.7 (0.469)	51.5 (0.346)
**Middle class**	47.1 (0.359)	50.5 (0.314)	42.5 (0.399)	49.9 (0.318)	42.5 (0.399)	49.9 (0.318)
**Poorer**	53 (0.309)	53.3 (0.287)	48.7 (0.335)	53.1 (0.288)	48.7 (0.335)	53.1 (0.288)
**Poorest**	57.4 (0.288)	58.1 (0.268)	55.8 (0.295)	57.8 (0.268)	55.8 (0.297)	57.8 (0.268)
**General**	57.3 (0.313)	57.1 (0.293)	57.2 (0.318)	57.6 (0.29)	49.9 (0.346)	57 (0.29)
**OBC**	49.2 (0.374)	51.6 (0.354)	49.9 (0.374)	51.8 (0.353)	45.9 (0.397)	51.1 (0.356)
**SC+ST**	51.9 (0.347)	54.1 (0.305)	52.4 (0.344)	54.2 (0.305)	47.5 (0.375)	53.7 (0.306)

Table S2.E: Estimated mean PWC (in μgm[-3]) (Coefficient of Variation, CV) of ambient $PM_{2.5}$ across the population subgroups in urban and rural regions of the states with low socio-demographic index (SDI) of NFHS-5 (2019-21). We followed the conventional NFHS framework (2 km buffer for urban and 5 km buffer for rural clusters) and performed two sensitivity assessments as well. In the 1st sensitivity assessment, we considered 2 km buffer for both urban and rural clusters; and in the 2nd sensitivity assessment, we considered 5 km buffer for both urban and rural clusters, respectively.

	NFHS framework		Sensitivity assessment 1		Sensitivity assessment 2	
	2 km urban-buffer and 5 km rural-buffer		2 km buffer for urban and rural clusters		5 km buffer for urban and rural clusters	
	Urban	Rural	Urban	Rural	Urban	Rural
**Male**	64.8 (0.228)	64.1 (0.214)	64.7 (0.233)	64 (0.215)	57.2 (0.251)	62 (0.222)
**Female**	65.7 (0.227)	64.7 (0.209)	65.2 (0.233)	64.6 (0.21)	59.1 (0.247)	62.7 (0.217)
**Richest**	53.8 (0.292)	66.9 (0.222)	59 (0.277)	67.3 (0.22)	47.5 (0.321)	64.8 (0.228)
**Richer**	55.5 (0.265)	65.1 (0.212)	60 (0.252)	65.4 (0.211)	50.5 (0.286)	63.1 (0.219)
**Middle class**	56.2 (0.248)	64.8 (0.208)	60 (0.238)	65.1 (0.207)	52.2 (0.263)	62.8 (0.215)
**Poorer**	57.8 (0.238)	64 (0.206)	60.1 (0.232)	64.4 (0.204)	53.5 (0.254)	62 (0.214)
**Poorest**	59.2 (0.226)	61.8 (0.213)	60.3 (0.226)	62.2 (0.197)	56.5 (0.236)	59.9 (0.22)
**General**	66.6 (0.224)	67 (0.197)	65.7 (0.23)	67 (0.197)	57.7 (0.255)	64.8 (0.204)
**OBC**	66.5 (0.223)	66.3 (0.2)	66.2 (0.229)	66.2 (0.2)	60.3 (0.238)	64.2 (0.207)
**SC+ST**	63.2 (0.236)	61.6 (0.222)	62.9 (0.242)	61.5 (0.223)	57.9 (0.028)	60.1 (0.228)

Table S2.F: Estimated mean PWC (in μgm[-3]) (Coefficient of Variation, CV) of ambient $PM_{2.5}$ across the population subgroups in urban and rural regions of the states with low socio-demographic index (SDI) of NFHS-4 (2015-16). We followed the conventional NFHS framework (2 km buffer for urban and 5 km buffer for rural clusters) and performed two sensitivity assessments as well. In the 1st sensitivity assessment, we considered 2 km buffer for both urban and rural clusters; and in the 2nd sensitivity assessment, we considered 5 km buffer for both urban and rural clusters, respectively.

	NFHS framework		Sensitivity assessment 1		Sensitivity assessment 2	
	2 km urban-buffer and 5 km rural-buffer		2 km buffer for urban and rural clusters		5 km buffer for urban and rural clusters	
	Urban	Rural	Urban	Rural	Urban	Rural
**Male**	68.7 (0.267)	68.5 (0.241)	70.1 (0.266)	68.6 (0.241)	61.8 (0.288)	66.7 (0.247)
**Female**	68.4 (0.266)	69.3 (0.237)	69.8 (0.266)	69.4 (0.236)	60.9 (0.289)	67.5 (0.242)
**Richest**	52.8 (0.357)	69.5 (0.257)	58.3 (0.337)	69.9 (0.257)	44.8 (0.401)	67.2 (0.259)
**Richer**	56.8 (0.319)	69.3 (0.236)	60.9 (0.305)	69.6 (0.235)	49.4 (0.355)	67.3 (0.241)
**Middle class**	58.9 (0.302)	68.8 (0.231)	62.5 (0.291)	69.1 (0.231)	53.1 (0.327)	66.7 (0.238)
**Poorer**	61.2 (0.279)	67.9 (0.232)	64 (0.271)	68.2 (0.232)	55.4 (0.304)	65.9 (0.24)
**Poorest**	64.2 (0.271)	68.5 (0.245)	66.4 (0.265)	68.7 (0.244)	59.6 (0.291)	66.8 (0.251)
**General**	69.1 (0.263)	72.3 (0.211)	59.6 (0.291)	70.1 (0.217)	59.6 (0.291)	70.1 (0.217)
**OBC**	69.7 (0.261)	71.3 (0.224)	62.5 (0.283)	69.4 (0.229)	62.5 (0.283)	69.4 (0.229)
**SC+ST**	65 (0.272)	64.6 (0.253)	59.4 (0.289)	63.1 (0.257)	59.4 (0.289)	63.1 (0.257)

Table S3.A: Estimated Z_{score} (95% confidence intervals, CIs) of ambient $PM_{2.5}$ exposure across the population subgroups in urban and rural regions of the states with high socio-demographic index (SDI) of NFHS-5 (2019-21). We followed the conventional NFHS framework (2 km buffer for urban and 5 km buffer for rural clusters) and performed two sensitivity assessments as well. In the 1st sensitivity assessment, we considered 2 km buffer for both urban and rural clusters; and in the 2nd sensitivity assessment, we considered 5 km buffer for both urban and rural clusters, respectively.

	Urban region			Rural region		
	NFHS framework	Sensitivity 1	Sensitivity 2	NFHS framework	Sensitivity 1	Sensitivity 2
	Z_{score} (95% CI)	Z_{score} (95% CI)	Z_{score} (95% CI)	Z_{score} (95% CI)	Z_{score} (95% CI)	Z_{score} (95% CI)
**Male – Female**	0.0059 (0.0039 – 0.0079)	-0.0001 (-0.0002 - -0.0001)	-0.0006 (-0.0007 - -0.0005)	-0.0145 (-0.0195 - -0.0095)	0.004 (0.0038 – 0.0042)	0.0002 (0.0001 – 0.0003)
**Richest – Richer**	-0.0312 (-0.0412 – -0.0212)	0.0063 (0.0061 – 0.0065)	-0.0088 (-0.0091 - -0.0085)	0.0441 (0.0351 – 0.0531)	0.0249 (0.0237 – 0.0261)	0.0214 (0.0207 – 0.0221)
**Richest – Middle class**	-0.0133 (-0.0163 - -0.0103)	0.0107 (0.0104 – 0.011)	-0.0096 (-0.0099 - -0.0093)	0.0982 (0.0662 – 0.1302)	0.0316 (0.0303 – 0.0329)	0.0266 (0.0255 – 0.0277)
**Richest – Poorer**	-0.0148 (-0.0188 - -0.0108)	0.0007 (0.0006 – 0.0008)	-0.0247 (-0.0256 - -0.0238)	0.1384 (0.0834 – 0.1934)	0.0254 (0.0244 – 0.0264)	0.0195 (0.0187 – 0.0203)
**Richest – Poorest**	-0.075 (-0.096 - -0.054)	0.0051 (0.0048 – 0.0054)	-0.021 (-0.0218 - -0.0202)	0.1339 (0.0869 – 0.1809)	0.0196 (0.0188 – 0.0204)	0.0104 (0.0099 – 0.0109)
**General – OBC**	0.0079 (0.0049 – 0.0109)	0.0405 (0.0388 – 0.0422)	0.0301 (0.029 – 0.0312)	-0.0569 (-0.0709 - -0.0429)	0.0298 (0.0288 – 0.0308)	0.0302 (0.0293 – 0.0311)
**General – (SC+ST)**	0.011 (0.008 – 0.014)	0.0155 (0.015 – 0.016)	0.0054 (0.052 – 0.056)	0.0277 (0.0167 – 0.0387)	0.007 (0.0067 – 0.0073)	0.0087 (0.0084 – 0.009)

Table S3.B: Estimated Z_{score} (95% confidence intervals, CIs) of ambient $PM_{2.5}$ exposure across the population subgroups in urban and rural regions of the states with high socio-demographic index (SDI) of NFHS-4 (2015-16). We followed the conventional NFHS framework (2 km buffer for urban and 5 km buffer for rural clusters) and performed two sensitivity assessments as well. In the 1st sensitivity assessment, we considered 2 km buffer for both urban and rural clusters; and in the 2nd sensitivity assessment, we considered 5 km buffer for both urban and rural clusters, respectively.

	Urban region			Rural region		
	NFHS framework	Sensitivity 1	Sensitivity 2	NFHS framework	Sensitivity 1	Sensitivity 2
	Z_{score} (95% CI)	Z_{score} (95% CI)	Z_{score} (95% CI)	Z_{score} (95% CI)	Z_{score} (95% CI)	Z_{score} (95% CI)
**Male – Female**	0.0064 (0.0062 – 0.0066)	-0.0082 (0.0078 – 0.0086)	-0.0021 (-0.0022 - -0.002)	-0.0152 (-0.0159 - -0.0145)	-0.002 (-0.0021 - -0.0019)	-0.0012 (-0.0013 - -0.0011)
**Richest – Richer**	-0.0282 (-0.0293 - -0.0271)	0.0084 (0.0081 – 0.0087)	-0.0008 (-0.0009 - -0.0007)	0.0402 (0.039 – 0.0414)	0.019 (0.0182 – 0.0198)	0.0169 (0.0162 – 0.0176)
**Richest – Middle class**	-0.0141 (-0.0147 - -0.0135)	0.0055 (0.0053 – 0.0057)	-0.0033 (-0.0034 - -0.0032)	0.0998 (0.095 – 0.1046)	0.0223 (0.0215 – 0.0231)	0.0198 (0.0191 – 0.0205)
**Richest – Poorer**	-0.0154 (-0.0161 - -0.0147)	0.0078 (0.0075 – 0.0081)	-0.0103 (-0.0107 - -0.0099)	0.1332 (0.1281 – 0.1383)	0.0181 (0.0173 – 0.0189)	0.0123 (0.0118 – 0.0128)
**Richest – Poorest**	-0.0654 (-0.0674 - -0.0634)	0.0086 (0.0083 – 0.0089)	-0.0289 (-0.0299 - -0.0279)	0.14 (0.1337 – 0.1463)	0.0161 (0.0156 – 0.0166)	0.0085 (0.0082 – 0.0088)
**General – OBC**	0.0076 (0.0076 – 0.0078)	0.0216 (0.0207 – 0.0225)	0.0163 (0.0157 – 0.0169)	-0.0597 (-0.0617 - -0.0577)	0.0148 (0.0143 – 0.0153)	0.0144 (0.0137 – 0.0151)
**General – (SC+ST)**	0.0121 (0.0117 – 0.0125)	0.0004 (0.0003 – 0.0005)	0.0032 (0.03 – 0.034)	0.0291 (0.0281 – 0.0301)	-0.0024 (-0.0025 – -0.0023)	0.0134 (0.0129 – 0.0139)

Table S3.C: Estimated Z_{score} (95% confidence intervals, CIs) of ambient $PM_{2.5}$ exposure across the population subgroups in urban and rural regions of the states with middle socio-demographic index (SDI) of NFHS-5 (2019-21). We followed the conventional NFHS framework (2 km buffer for urban and 5 km buffer for rural clusters) and performed two sensitivity assessments as well. In the 1st sensitivity assessment, we considered 2 km buffer for both urban and rural clusters; and in the 2nd sensitivity assessment, we considered 5 km buffer for both urban and rural clusters, respectively.

	Urban region			Rural region		
	NFHS framework	Sensitivity 1	Sensitivity 2	NFHS framework	Sensitivity 1	Sensitivity 2
	Z_{score} (95% CI)	Z_{score} (95% CI)	Z_{score} (95% CI)	Z_{score} (95% CI)	Z_{score} (95% CI)	Z_{score} (95% CI)
**Male – Female**	-0.0184<br>(-0.0234 - -0.0134)	0.0022<br>(0.021 - 0.0023)	0.0022<br>(0.0021 – 0.0023)	-0.0132<br>(-0.0182 - -0.0082)	-0.001<br>(-0.002 – -0.001)	-0.0006<br>(-0.0007 - -0.0005)
**Richest – Richer**	0.0425<br>(0.0255 – 0.0595)	-0.0015<br>(-0.0016 - -0.0014)	-0.0056<br>(-0.0058 - -0.0054)	0.0135<br>(0.0095 – 0.0175)	0.0342<br>(0.033 – 0.0354)	0.0303<br>(0.0294 – 0.0312)
**Richest – Middle class**	0.071<br>(0.054 – 0.088)	-0.0137<br>(-0.0144 - -0.013)	-0.0184<br>(-0.0191 - -0.0177)	-0.0341<br>(-0.0421 - -0.0261)	0.0422<br>(0.0405 – 0.0439)	0.0381<br>(0.037 – 0.0392)
**Richest – Poorer**	0.0261<br>(0.0201 – 0.0321)	-0.0321<br>(-0.0333 - -0.0309)	-0.0401<br>(-0.0419 - -0.0383)	-0.0567<br>(-0.0787 - -0.0347)	0.0254<br>(0.0242 – 0.0266)	0.0218<br>(0.0208 – 0.0228)
**Richest – Poorest**	0.0725<br>(0.0555 – 0.0895)	-0.0491<br>(-0.051 - -0.0472)	-0.0546<br>(-0.0565 - -0.0527)	-0.0441<br>(-0.0591 - -0.0291)	0.0005<br>(0.0004 – 0.0006)	-0.0026<br>(-0.0027 - -0.0025)
**General – OBC**	0.0536<br>(0.0346 – 0.0726)	0.0482<br>(0.0467 – 0.0497)	0.0352<br>(0.034 – 0.0364)	-0.0316<br>(-0.0406 - -0.0226)	0.0425<br>(0.0408 – 0.0442)	0.0393<br>(0.0378 – 0.0408)
**General – (SC+ST)**	0.0054<br>(0.0034 – 0.0074)	0.0198<br>(0.019 – 0.0206)	0.006<br>(0.0058 – 0.0062)	0.0168<br>(0.0118 – 0.0218)	0.0196<br>(0.0186 – 0.0206)	0.019<br>(0.0183 – 0.0197)

Table S3.D: Estimated Z_{score} (95% confidence intervals, CIs) of ambient $PM_{2.5}$ exposure across the population subgroups in urban and rural regions of the states with middle socio-demographic index (SDI) of NFHS-4 (2015-16). We followed the conventional NFHS framework (2 km buffer for urban and 5 km buffer for rural clusters) and performed two sensitivity assessments as well. In the 1st sensitivity assessment, we considered 2 km buffer for both urban and rural clusters; and in the 2nd sensitivity assessment, we considered 5 km buffer for both urban and rural clusters, respectively.

	Urban region			Rural region		
	NFHS framework	Sensitivity 1	Sensitivity 2	NFHS framework	Sensitivity 1	Sensitivity 2
	Z_{score} (95% CI)	Z_{score} (95% CI)	Z_{score} (95% CI)	Z_{score} (95% CI)	Z_{score} (95% CI)	Z_{score} (95% CI)
**Male – Female**	-0.0189<br>(-0.0197 - -0.0181)	0.0043<br>(0.0042 – 0.0044)	0.0043<br>(0.0041 – 0.0045)	-0.0134<br>(-0.0141 - -0.0127)	0.0008<br>(0.0007 – 0.0009)	0.0008<br>(0.0007 – 0.0009)
**Richest – Richer**	0.034<br>(0.0328 – 0.0352)	0.0019<br>(0.0018 – 0.002)	0.0019<br>(0.0018 – 0.002)	0.0149<br>(0.0143 – 0.0155)	0.0297<br>(0.0283 – 0.0311)	0.0297<br>(0.0285 – 0.0309)
**Richest – Middle class**	0.0565<br>(0.054 – 0.059)	-0.017<br>(-0.0176 - -0.017)	-0.017<br>(-0.0177 - -0.0163)	-0.0374<br>(-0.0386 - -0.0362)	0.0375<br>(0.0361 – 0.0389)	0.0375<br>(0.036 – 0.039)
**Richest – Poorer**	0.0278<br>(0.0267 – 0.0289)	-0.0422<br>(-0.0438 - -0.0406)	-0.0422<br>(-0.0442 – 0.0402)	-0.0618<br>(-0.0648 - -0.0588)	0.0253<br>(0.0245 – 0.0261)	0.0253<br>(0.024 – 0.0266)
**Richest – Poorest**	0.0756<br>(0.0725 – 0.0787)	-0.0703<br>(-0.0726 - -0.068)	-0.0703<br>(-0.0726 - -0.068)	-0.0481<br>(-0.0501 - -0.0461)	0.0064<br>(0.0061 – 0.0067)	0.0064<br>(0.0061 – 0.0067)
**General – OBC**	0.0387<br>(0.0372 – 0.0402)	0.028<br>(0.0271 – 0.0289)	0.0159<br>(0.0154 – 0.0164)	-0.0347<br>(-0.0357 - -0.0337)	0.0234<br>(0.0225 – 0.0243)	0.024<br>(0.0228 – 0.0252)
**General – (SC+ST)**	0.0059<br>(0.0056 – 0.0062)	0.0187<br>(0.0179 – 0.0195)	0.0097<br>(0.0093 – 0.0101)	0.0186<br>(0.0178 – 0.0194)	0.0145<br>(0.014 – 0.015)	0.0142<br>(0.0136 – 0.0148)

Table S3.E: Estimated Z_{score} (95% confidence intervals, CIs) of ambient $PM_{2.5}$ exposure across the population subgroups in urban and rural regions of the states with low socio-demographic index (SDI) of NFHS-5 (2019-21). We followed the conventional NFHS framework (2 km buffer for urban and 5 km buffer for rural clusters) and performed two sensitivity assessments as well. In the 1st sensitivity assessment, we considered 2 km buffer for both urban and rural clusters; and in the 2nd sensitivity assessment, we considered 5 km buffer for both urban and rural clusters, respectively.

	Urban region			Rural region		
	NFHS framework	Sensitivity 1	Sensitivity 2	NFHS framework	Sensitivity 1	Sensitivity 2
	Z_{score} (95% CI)	Z_{score} (95% CI)	Z_{score} (95% CI)	Z_{score} (95% CI)	Z_{score} (95% CI)	Z_{score} (95% CI)
**Male – Female**	0.0141 (0.0091 – 0.0191)	-0.0023 (-0.0024 - -0.0022)	-0.0091 (-0.0094 - -0.0088)	0.012 (0.007 – 0.017)	-0.0032 (-0.0032 - -0.0031)	-0.0037 (-0.0038 - -0.0036)
**Richest – Richer**	0.007 (0.005 – 0.009)	-0.0045 (-0.0046 - -0.0044)	-0.0143 (-0.015 - -0.0136)	-0.021 (-0.029 - -0.013)	0.0094 (0.009 – 0.0098)	0.0084 (0.008 – 0.0088)
**Richest – Middle class**	0.0317 (0.0247 – 0.0387)	-0.0046 (-0.0048 - -0.0044)	-0.0229 (-0.0237 - -0.0221)	0.0124 (0.0084 – 0.0164)	0.011 (0.0106 – 0.0114)	0.01 (0.008 – 0.012)
**Richest – Poorer**	-0.0136 (-0.0186 - -0.0086)	-0.0051 (-0.0053 - -0.0049)	-0.0294 (-0.0307 - -0.0281)	-0.0063 (-0.0073 - -0.0053)	0.0147 (0.0142 – 0.0152)	0.0141 (0.0136 – 0.0146)
**Richest – Poorest**	0.047 (0.0037 – 0.0057)	-0.0061 (0.0063 - -0.0059)	-0.0444 (-0.0457 - -0.0431)	0.0185 (0.0135 – 0.0235)	0.0258 (0.0258 – 0.0271)	0.0247 (0.0239 – 0.0255)
**General – OBC**	-0.0229 (-0.0309 - -0.0149)	-0.0023 (0.0024 - -0.0022)	-0.0126 (-0.0131 - -0.0121)	-0.0291 (-0.0371 - -0.0211)	0.0043 (0.0041 – 0.0045)	0.0032 (0.003 – 0.0034)
**General – (SC+ST)**	0.0166 (0.0116 – 0.0216)	0.013 (0.0124 – 0.0136)	-0.0009 (-0.001 - -0.0008)	0.0413 (0.0273 – 0.0553)	0.0289 (0.0279 – 0.0299)	0.0247 (0.0237 – 0.0257)

Table S3.F: Estimated Z_{score} (95% confidence intervals, CIs) of ambient $PM_{2.5}$ exposure across the population subgroups in urban and rural regions of the states with low socio-demographic index (SDI) of NFHS-4 (2015-16). We followed the conventional NFHS framework (2 km buffer for urban and 5 km buffer for rural clusters) and performed two sensitivity assessments as well. In the 1st sensitivity assessment, we considered 2 km buffer for both urban and rural clusters; and in the 2nd sensitivity assessment, we considered 5 km buffer for both urban and rural clusters, respectively.

	Urban region			Rural region		
	NFHS framework	Sensitivity 1	Sensitivity 2	NFHS framework	Sensitivity 1	Sensitivity 2
	Z_{score} (95% CI)	Z_{score} (95% CI)	Z_{score} (95% CI)	Z_{score} (95% CI)	Z_{score} (95% CI)	Z_{score} (95% CI)
**Male – Female**	0.0153 (0.0147 – 0.0159)	0.0013 (0.0012 – 0.0014)	0.0036 (0.0035 – 0.0037)	0.0129 (0.0124 – 0.0134)	-0.0038 (-0.004 – 0.0036)	-0.0031 (-0.0032 - -0.003)
**Richest – Richer**	0.0065 (0.0063 – 0.0067)	-0.0096 (-0.0099 – 0.0093)	-0.0183 (-0.0189 – -0.0177)	-0.0159 (-0.0165 - -0.0153)	0.0012 (0.0011 – 0.0013)	-0.0004 (-0.0005 - -0.0003)
**Richest – Middle class**	0.03 (0.0285 – 0.0315)	-0.0157 (-0.0163 – 0.0151)	-0.0332 (-0.0345 – 0.0319)	0.0135 (0.013 – 0.014)	0.0033 (0.0032 – 0.0034)	0.0021 (0.002 – 0.0022)
**Richest – Poorer**	-0.0149 (-0.0156 - -0.0142)	-0.0218 (-0.0228 – 0.0208)	-0.0431 (-0.0446 - -0.0416)	-0.007 (-0.0073 - -0.0067)	0.0071 (0.0068 – 0.0074)	0.0055 (0.0053 – 0.0057)
**Richest – Poorest**	0.0051 (0.0049 – 0.0053)	-0.0307 (-0.032 - -0.0294)	-0.0593 (-0.0615 - -0.0571)	0.0204 (0.0197 – 0.0211)	0.0049 (0.0047 – 0.0051)	0.0017 (0.0016 – 0.0018)
**General – OBC**	-0.0245 (-0.0256 - -0.0234)	-0.0117 (-0.0121 - -0.0113)	-0.0117 (-0.0121 - -0.0113)	-0.0284 (-0.0296 - -0.0272)	0.0032 (0.0031 – 0.0033)	0.0032 (0.003 – 0.0034)
**General – (SC+ST)**	0.0174 (0.0165 – 0.0183)	0.0008 (0.0007 – 0.0009)	0.0008 (0.0007 – 0.0009)	0.0451 (0.0435 – 0.0467)	0.0314 (0.0304 – 0.0324)	0.0314 (0.03 – 0.0328)

Table S4.A: Estimated mean subgroup-weighted ambient $PM_{2.5}$ exposure (PWC, in μgm^{-3}) across the population subgroups in urban regions of the states in NFHS-5 (2019-21). We followed the conventional NFHS framework of assigning 2 km buffer for urban and 5 km buffer for rural clusters, respectively.

State	Male	Female	Richest	Richer	Middle class	Poorer	Poorest	General	OBC	SC+ST
Andhra Pradesh	37.8	37.7	35.1	35.3	36.3	34.8	35.1	37.7	37.9	37
Arunachal Pradesh	45.8	44.9	26.4	26.7	26.5	28.5	32.8	50.9	50.7	43.4
Assam	58.1	58.3	42.7	47	46.4	42.6	42.9	58.4	57.8	57.4
Bihar	69.8	70	62.5	65.9	66	65.6	67.3	69.7	70.3	69.3
Chhattisgarh	44.9	44.5	36.4	37.2	36.8	40.1	36.3	45.6	45	43.6
Delhi	99.4	100.3	24.3	25.8	24.8	32.5	32.8	100.1	99.6	100.1
Goa	35.8	35.8	28.5	30.8	31.6	20.7	26.3	36	35.9	36.1
Gujarat	51.7	51.3	35.7	37.8	40.3	42.8	43	51.5	51.1	51.4
Haryana	79.6	75	50.6	54.9	61.2	63.1	58.1	75.8	77.2	73.9
Himachal Pradesh	46	45.9	41.9	38.5	39.2	41.9	18.7	46.1	45.8	45.2
Jammu & Kashmir	43.9	44.3	29	22.9	27.5	30.7	36.1	44.3	42.9	46.6
Jharkhand	52.9	53.3	42.3	44.3	43.9	44.1	45.9	54.3	53.8	51.9
Karnataka	30	30	24.5	24.3	26.2	26.5	27.6	30.2	29.9	29.9
Kerala	30.1	30.3	27.9	28.7	28.9	29.1	30.2	30.3	30.3	30.3
Madhya Pradesh	48	47.9	39.1	40.2	42.3	45.4	43.2	48.7	48.3	46.4
Maharashtra	43.2	43.3	35.2	34.4	32.6	37.2	34.9	43.6	43.2	42.7
Manipur	36.4	33.5	13.2	16.6	15.3	20.1	23.4	33.3	33.1	34.4
Meghalaya	34.8	32.3	15.9	18.2	20.6	29.9	32.3	24.6	24.6	33.9
Mizoram	33.5	32.3	15.5	19.2	21.5	23.7	17.8	0	37.1	32.5
Nagaland	42.7	40.1	16.2	17.1	14.7	18.6	20.1	34.7	52.8	39.3
Orissa	47.9	47.8	43.1	41.7	42.5	44	47	48	47.9	47.6
Punjab	52.4	52.6	42.1	42.2	43.2	43	30.4	52.6	52.4	52.6
Rajasthan	59.8	58.6	50.8	53	53.3	56.3	60.2	58.5	58.8	58.9
Sikkim	34.7	35.1	11.5	15	19.6	19.8	8	36	35.1	34.7
Tamil Nadu	33.7	33.6	24.7	29	30.2	30.9	32.4	34.6	33.6	33.5
Telengana	41.2	41.1	29.2	31.3	30.3	33.8	33	40.8	41.1	41.4
Tripura	49.7	49.9	28.7	32.4	37.3	42	43.7	49.9	49.9	49.9
Uttar Pradesh	78.9	79.6	61.3	63.2	66.1	71.9	70.6	80.3	79.2	79.3
Uttarakhand	60.8	61.8	50.5	53.2	60.5	63.7	50.8	60.6	65.2	59.7
West Bengal	56.4	57.1	47.2	47.7	49.1	52.5	53.7	56.9	56.3	57.7

Table S4.B: Estimated mean subgroup-weighted ambient $PM_{2.5}$ exposure (PWC, in μgm^{-3}) across the population subgroups in rural regions of the states in NFHS-5 (2019-21). We followed the conventional NFHS framework of assigning 2 km buffer for urban and 5 km buffer for rural clusters, respectively.

State	Male	Female	Richest	Richer	Middle class	Poorer	Poorest	General	OBC	SC+ST
Andhra Pradesh	37.5	37.7	37.1	37.4	37.7	38	37.2	37.8	37.8	37.2
Arunachal Pradesh	43.5	42.8	39.4	36.6	37.2	37.6	40.7	50.1	48.4	41.9
Assam	57.6	57.5	57.5	56.6	55.7	54.4	53.3	58.5	57.4	56.5
Bihar	68.3	68.5	68.8	67.7	68.2	67.8	67.4	68.6	68.6	68.1
Chhattisgarh	44.2	44.1	44.4	44.2	43.7	43.8	41.7	44.9	45.3	43
Delhi	92.1	97	82.1	98.2	95.2	57.4	0	98.6	94.6	95.6
Goa	35.5	35.8	33.7	34.7	34.5	36	23.4	35.8	35.9	35.4
Gujarat	50.7	50.7	51	51.2	50.9	50.3	49.6	51	51.3	49.6
Haryana	70	69.8	68.5	69	68.6	68.9	68.7	70.3	70.2	68.8
Himachal Pradesh	45.3	45.4	44.8	45	45	44.9	45.1	45.4	46.3	44.9
Jammu & Kashmir	42.7	42.7	43.2	39.7	38.5	38.6	39.1	42.8	41.3	44
Jharkhand	52	51.9	50.2	50.5	50.2	50.6	51.4	53.8	52.6	50.9
Karnataka	30.3	30.3	29.3	29.5	29.6	30.8	31.6	30	30.2	30.4
Kerala	30	30.1	29.9	29.8	29.7	28.8	27.3	30.1	30.3	29.5
Madhya Pradesh	47.7	47.9	48.4	48.3	48.3	47.7	46.2	50.1	48.3	46.8
Maharashtra	41.7	41.6	41.3	41.5	41.3	41.5	42.1	40.1	42.4	42.3
Manipur	33.2	32.8	21.2	26	26.2	28.5	30.7	33.4	33.6	32
Meghalaya	40.1	40.5	30.3	37	39.8	37.8	37.9	46.3	56.2	40.2
Mizoram	37.7	37.1	33.6	33.2	34	35.8	40.6	0	43.8	37.3
Nagaland	36.6	37.3	36.2	32.9	33.7	32.8	31.6	32.3	50.2	37.1
Orissa	47	47.2	46.9	47.6	47.5	47.2	46.6	48.5	47.9	46.3
Punjab	52.6	52.7	50.9	51.9	51.5	51.9	51.3	52.9	52.5	52.6
Rajasthan	60.9	60.5	60	61	61.4	60.9	58.2	61.7	61.3	59.1
Sikkim	36.8	36.5	32.1	33.2	33.1	33.3	33.6	36.4	36.6	36.5
Tamil Nadu	33.5	33.7	33.4	33.3	33.2	33.4	32.9	33.9	33.7	33.6
Telengana	40.1	40.1	39.6	40	39.8	39.6	39.9	40.2	40.1	40.2
Tripura	50.4	50.3	50	40.7	41.9	43.7	45.8	50.1	50.1	50.4
Uttar Pradesh	76.9	76.7	77.4	76.4	76	75.3	74.5	77.4	76.8	76.2
Uttarakhand	59.3	57.9	56.9	56.4	52.5	51.9	53.1	52.3	65	59.5
West Bengal	56	56.2	57.1	56.2	55.7	56.1	56.2	55.5	56.4	56.6

Table S4.C: Estimated mean subgroup-weighted ambient $PM_{2.5}$ exposure (PWC, in μgm^{-3}) across the population subgroups in urban regions of the states in NFHS-4 (2015-16). We followed the conventional NFHS framework of assigning 2 km buffer for urban and 5 km buffer for rural clusters, respectively.

State	Male	Female	Richest	Richer	Middle class	Poorer	Poorest	General	OBC	SC+ST
Andhra Pradesh	45.9	45.7	44.6	43.3	42.1	45.3	43.2	46.3	45.5	45.6
Arunachal Pradesh	42.6	41.1	25.1	25.1	25.1	27.3	30.3	47.4	47.9	40.1
Assam	58.9	58.9	46.6	51.5	53.7	57.4	57	58.2	57.9	58.5
Bihar	78.7	81.5	60.4	73.3	76.1	77.1	80.9	78.6	81.2	84
Chhattisgarh	46.2	45.8	35.8	38.3	40.7	39.7	39.4	45.9	46.1	45.2
Delhi	109.5	107.9	54.6	55.1	63.2	47.8	17.9	105.3	107.5	111.9
Goa	40.3	39.9	36.6	33.9	34	30.1	42	38.7	41	41.9
Gujarat	55.7	55.2	43.9	46.4	51	51.9	44.1	55.3	56.2	53.4
Haryana	82.8	84.1	66.3	75.4	72.8	78.9	69.2	82.2	85.1	84.1
Himachal Pradesh	48.4	46.9	45.8	46.4	46	45.8	49.7	46.9	53.2	46.2
Jammu & Kashmir	40.9	41.2	34.4	26.8	31.1	34.8	37.7	48.3	51.1	47
Jharkhand	59.9	58.6	41.1	43.4	42.1	49.2	46.6	58.7	59.3	57.7
Karnataka	33.3	33	24.5	29.2	31.1	32.8	32	33.8	33.3	32.6
Kerala	36.2	36.3	34.6	34.7	35.4	35.4	36.7	36.5	36.3	36.3
Madhya Pradesh	49.8	50	37.4	39.2	43.6	45.9	46.1	50.7	50.2	49.1
Maharashtra	43.7	44.2	38.2	37.5	37.1	38.8	42.3	43.4	45	44.9
Manipur	32.6	28.3	8.1	10.1	13.6	15.2	18.8	28.9	27.5	30.1
Meghalaya	35	35.1	17	17.9	22.5	31.8	35.8	33.8	55.6	35.4
Mizoram	30.7	29.5	15.6	16.2	22.4	28.2	25.7	27	28.9	29.6
Nagaland	31	32.7	13	14.9	18.6	21.7	22.3	42.2	38.6	31.9
Orissa	33.6	34.7	20.5	14.9	7.5	9.2	15.9	41.8	39.6	13.2
Punjab	61.7	61.5	53.1	55.7	57.1	57.6	60.5	61.1	61.6	62
Rajasthan	64.7	64.6	53.6	57.8	61.4	63.3	60.2	64	65.4	64
Sikkim	29.2	27.7	11.8	11.5	12	13.2	3	28.8	28.1	27.2
Tamil Nadu	40.2	40.6	32	35	36.3	38.1	38.6	42.4	40.7	39.9
Telengana	39.9	41.3	35.8	34	36.9	42.7	43.9	41.2	41.9	38.4
Tripura	55.3	55.1	23.3	29.7	33.3	37.8	48.1	55.3	55	55
Uttar Pradesh	84	82.9	64	66.6	69.2	72.5	72.8	83.3	82.7	83.8
Uttarakhand	61.5	61.1	43.5	46.5	55.1	59.8	60.3	56.7	69.4	59.1
West Bengal	65.5	65.3	58.8	59	60.2	62.9	65.4	65.2	62.8	65.7

Table S4.D: Estimated mean subgroup-weighted ambient $PM_{2.5}$ exposure (PWC, in μgm^{-3}) across the population subgroups in rural regions of the states in NFHS-4 (2015-16). We followed the conventional NFHS framework of assigning 2 km buffer for urban and 5 km buffer for rural clusters, respectively.

State	Male	Female	Richest	Richer	Middle class	Poorer	Poorest	General	OBC	SC+ST
Andhra Pradesh	45.9	46.1	45.9	46.2	46.3	45.8	45.5	45.6	46.4	46.1
Arunachal Pradesh	40.5	40.1	36.7	35	35.8	37.5	38	47	45.8	38.3
Assam	58.9	59.4	56.2	57.6	57.3	57.7	57.9	59.8	57.5	58.4
Bihar	83	82.9	81.9	82.4	82.5	82.5	82.1	83.2	83.1	82.3
Chhattisgarh	45.7	45.2	45.8	45.6	45.8	44.9	43.5	43.8	46.4	44.3
Delhi	103.6	100.4	95.9	100.4	118.4	94.2	0	103.5	96.7	102.3
Goa	42.3	42.3	41.5	41.8	42.1	42.4	42.1	42.5	42.4	42
Gujarat	57.8	57.6	58.1	57.7	57.7	56.9	55.9	58.8	58.8	55.4
Haryana	81	79.8	78.9	78.7	78	79.2	80.5	80.2	80.3	79.4
Himachal Pradesh	47.4	46.6	46.8	45.9	45.8	44.9	44	46.4	48.4	46.7
Jammu & Kashmir	40.8	40.9	43.7	39.7	37.7	36.7	36.5	43.6	41.3	44.2
Jharkhand	58.4	57.7	54.7	55.1	55.1	56.4	57.4	57.4	58.6	56.7
Karnataka	33.1	33.3	34.1	33	33.1	33.3	33	33.1	33.7	32.9
Kerala	36.2	36.4	36.7	36.3	35.8	34.3	33.6	36.7	36.4	35.5
Madhya Pradesh	50.5	50.2	50.3	50.8	51	50.8	49	52.7	51.2	48.7
Maharashtra	45.7	45.5	45.4	45.3	45.3	45.6	46.1	44.1	46.7	46
Manipur	30.3	30.1	19	19.1	21.9	23.5	28.5	31.7	30.5	29.6
Meghalaya	43.6	43.3	44.2	40.2	39.8	43.9	44.1	48.7	40	41.8
Mizoram	36.2	36.8	33.2	34.3	34.3	37.8	44.4	35.6	45.6	36.2
Nagaland	35.6	36.2	32.2	32.6	32.7	31.9	34	46.9	49.8	35.7
Orissa	41.5	41.9	36.5	37.4	38.5	38.8	37.6	43.8	41.7	41.8
Punjab	61.7	61.8	60.8	60.9	61.4	62.1	60.5	62	61.2	61.9
Rajasthan	66.8	66.4	66.8	66.5	66.9	66.8	63.7	67.8	67.7	64.2
Sikkim	28.3	28.9	23.8	24.9	24.4	23.5	25.7	28.7	29.4	28.5
Tamil Nadu	41.4	42.1	41.6	41.1	40.9	42.2	42	40.7	41.9	42.1
Telengana	45.1	44.5	44.8	45.1	44.8	44	44.1	43.8	44.5	45
Tripura	55.1	55	43	50.8	53.6	53.2	53.1	55.2	55	54.9
Uttar Pradesh	80.5	80.3	83.4	81.5	80.6	79.4	78.4	80.8	80.4	79.8
Uttarakhand	60.2	57.5	59	57.4	55.1	55.8	56.7	49.5	67.2	62.8
West Bengal	66	65.4	64.9	65.3	64.6	65.3	66	64.2	65.5	65.9

Table S4.E: Estimated mean subgroup-weighted ambient $PM_{2.5}$ exposure (PWC, in μgm^{-3}) across the population subgroups in urban regions of the states in NFHS-5 (2019-21). We performed the 1st sensitivity analysis of assigning 2 km buffers for both urban and rural clusters, respectively.

State	Male	Female	Richest	Richer	Middle class	Poorer	Poorest	General	OBC	SC+ST
Andhra Pradesh	38	37	36.5	35.8	37	34.9	36.6	37.4	37	37.2
Arunachal Pradesh	45.8	43.7	29.4	30.1	29.1	31.2	35.8	48.8	50.1	42.7
Assam	56.6	57.5	45.4	49.9	49.6	44.4	44.1	58	57.3	55.6
Bihar	70.8	70.7	64.4	68.4	69.6	69.6	69.2	70.7	70.9	70
Chhattisgarh	44.8	44.2	37.7	39.1	38.8	41.3	36.6	45.4	44.6	43.2
Delhi	90.2	94.9	40.4	36.3	35.6	42.8	36.9	95.7	91.7	91.9
Goa	35.8	35.7	33.5	35.1	35.3	29.8	37.4	35.8	35.7	36
Gujarat	45.4	48.1	35.6	37.6	38	39	43.3	46.7	47.5	48.9
Haryana	77.8	74.2	56.8	61.2	63.7	68.4	61.9	73.9	76.8	73.9
Himachal Pradesh	41.9	44.6	41.7	38.6	37.6	41.8	18.8	44.9	43.6	43.1
Jammu & Kashmir	43.6	43.9	30.7	25.9	29.2	31.7	37	44	42.5	46.6
Jharkhand	53.1	52.4	47.1	47.1	47.3	47.7	49	53.1	52.7	52
Karnataka	29.3	29	26	25.2	27.5	27.9	29.3	28.3	29.2	28.9
Kerala	30.1	30.3	29.5	29.9	29.5	29.2	30	30.3	30.3	30.2
Madhya Pradesh	47.4	46.9	40.2	41.9	43.7	45.6	42.3	47.7	47.6	45
Maharashtra	42	42.8	37.3	35.7	32.7	37.7	35.4	43.2	42.5	42
Manipur	36.2	33.2	14.2	19.1	19.6	24.2	26.5	33.1	32.2	34.2
Meghalaya	34.1	31.5	17.8	20.1	21	30.8	32.8	23.2	23.2	33.1
Mizoram	33.3	30.5	18.1	22.7	23.8	24.7	17.4	0	35.5	30.8
Nagaland	43.4	40	25	22.2	17.3	21.4	24.3	33.6	53.3	39.7
Orissa	48.1	46.8	45.3	43.4	44.2	45.4	47.1	45.7	47.8	47.3
Punjab	52.3	52.1	45.3	47.3	48	47.4	46.7	51.9	52	52.3
Rajasthan	59.5	57.8	52.8	55.9	56.3	57.5	60.5	57.7	58.1	58.1
Sikkim	34.3	32.5	25.1	24.2	24.3	19.8	7.9	30	33.2	32.1
Tamil Nadu	32.8	33.4	27.2	30.8	31.1	31.1	32.4	34.8	33.5	32.8
Telengana	40.2	40.5	32	33.5	34.2	35.7	37.4	38.8	40.5	41
Tripura	43.1	46.3	44.2	38.8	39.8	44.3	42.4	47.1	47	46.8
Uttar Pradesh	78.7	79.2	70.1	70.9	72.1	74.1	71.6	78.8	79	79.6
Uttarakhand	60.9	62	51.8	55.7	62.4	64.8	55.3	60.7	65.3	60.1
West Bengal	56.8	56.8	51.3	50.9	50.4	52.9	54.3	57.3	56.6	57.2

Table S4.F: Estimated mean subgroup-weighted ambient $PM_{2.5}$ exposure (PWC, in μgm^{-3}) across the population subgroups in rural regions of the states in NFHS-5 (2019-21). We performed the 1st sensitivity analysis of assigning 2 km buffers for both urban and rural clusters, respectively.

State	Male	Female	Richest	Richer	Middle class	Poorer	Poorest	General	OBC	SC+ST
Andhra Pradesh	37.4	37.7	37.1	37.4	37.7	38.1	37.2	37.7	37.8	37.2
Arunachal Pradesh	43.5	42.6	39.5	38.3	38.8	39.5	42.1	50.3	48	41.7
Assam	57.5	57.4	57.8	57	56.6	55.5	54.6	58.5	57.3	56.4
Bihar	68.3	68.5	69.5	68.5	68.8	68.4	68	68.6	68.6	68.1
Chhattisgarh	44.3	44	44.5	44.5	43.7	44	41.7	44.9	45.2	42.9
Delhi	93.1	96.7	93.1	98.4	99.6	92	0	98.3	94.2	96
Goa	35.4	35.7	35.7	35.7	35.8	36	36.2	35.8	36	35.3
Gujarat	50.6	50.7	51	51.3	50.9	50.4	49.6	51	51.3	49.6
Haryana	69.5	69.5	69.1	69.1	68.9	69.1	69.4	70	69.8	68.6
Himachal Pradesh	45.3	45.4	45.1	45.2	45.1	45	45.2	45.4	46.3	44.9
Jammu & Kashmir	42.4	42.5	44	41.5	40.6	40.1	40.7	42.5	41.2	43.8
Jharkhand	52	51.7	50.1	50.3	50.4	51.2	51.7	53.8	52.2	50.8
Karnataka	30.2	30.2	29.7	29.6	29.7	30.8	31.6	30	30.1	30.4
Kerala	30	30.1	30.1	30.1	29.9	29.2	27.3	30.1	30.3	29.5
Madhya Pradesh	47.7	47.8	48.4	48.5	48.3	47.7	46.1	50	48.2	46.7
Maharashtra	41.7	41.7	41.4	41.6	41.4	41.6	42.3	40.2	42.4	42.3
Manipur	33.1	32.7	24	28.5	29.1	31.3	32.3	33.2	33.7	31.8
Meghalaya	40.1	40.6	30.1	38.1	41.4	38.9	39.1	46.3	56.3	40.2
Mizoram	37.3	36.3	33	32.8	33.6	35	40	0	43	36.5
Nagaland	36.1	37.1	41.1	38.9	36.5	33.3	32.6	32.1	51.1	36.8
Orissa	47	47.2	47.1	47.8	47.6	47.3	46.7	48.5	47.9	46.4
Punjab	52.6	52.6	51.5	52.2	51.9	52	52.2	52.9	52.1	52.5
Rajasthan	60.8	60.5	60	61	61.4	60.9	58.2	61.7	61.3	59.1
Sikkim	36.9	36.4	34.8	34.4	35.4	34.8	35.2	36.8	36.5	36.3
Tamil Nadu	33.4	33.7	33.6	33.5	33.5	33.7	33	33.9	33.7	33.6
Telengana	40	40.1	39.8	40.3	39.9	39.7	39.9	40.2	40.1	40.2
Tripura	49.6	50	49.8	43.8	45.1	45.7	47.5	49.8	50	50.1
Uttar Pradesh	76.5	76.5	78.1	76.9	76.4	75.8	75.1	77.3	76.5	76
Uttarakhand	58.7	57.6	60	57.4	53.2	52.8	53.5	51.7	64.9	59.3
West Bengal	55.6	55.9	55.4	55.6	55.5	56	56.1	55	56.2	56.4

Table S4.G: Estimated mean subgroup-weighted ambient $PM_{2.5}$ exposure (PWC, in μgm^{-3}) across the population subgroups in urban regions of the states in NFHS-4 (2015-16). We performed the 1st sensitivity analysis of assigning 2 km buffers for both urban and rural clusters, respectively.

State	Male	Female	Richest	Richer	Middle class	Poorer	Poorest	General	OBC	SC+ST
Andhra Pradesh	47.1	46.3	45.5	44.6	43.8	45.4	43.1	47	46.3	46.1
Arunachal Pradesh	43.4	40.9	27	27.1	27.4	28.7	30.7	46.8	47.8	40.2
Assam	59.3	58.8	50.7	54.5	55.4	58.3	58.2	58.1	58.3	57.7
Bihar	82.3	84.8	73.4	78	80.5	81.6	82	84.4	84.5	84.2
Chhattisgarh	46.1	45.6	38.9	40.1	41.6	40.8	40.1	45.9	45.7	45.2
Delhi	111.2	110.8	67.5	74.8	79.3	72	40	111	110.3	111.9
Goa	42.4	42.4	38.9	38.9	37.1	33	41.8	42.4	42.7	42.5
Gujarat	55.5	55.9	52	52.3	54	52.8	45.3	54.9	57	55.5
Haryana	84.3	85.1	69.7	75.9	75.7	79.7	67.3	83.5	86.5	84.2
Himachal Pradesh	49.2	47.4	46.6	46.8	45.9	46.1	50.8	47.4	54.4	46.7
Jammu & Kashmir	41.5	41.8	38.4	30.1	34.6	36.3	39.5	48.8	51	47.9
Jharkhand	60	60	45	48.9	48.3	54.1	52.9	60	60.8	58.4
Karnataka	33.1	33.2	28.7	29.3	31.6	33.1	32.3	33.8	33.3	32.9
Kerala	36.7	37.1	36.1	35.5	35.9	35.5	36.8	37.4	36.9	37.2
Madhya Pradesh	49.9	49.9	39	40.8	44.4	46.5	46.6	49.7	50.4	49.2
Maharashtra	45.2	45.4	39.8	40.1	40.1	41.5	42.2	45.1	45.9	45.4
Manipur	32.3	29.9	10.4	12.6	16.8	18.8	21.2	29.7	29.7	31.7
Meghalaya	34.9	34.8	17.3	18.7	21.7	31.1	36	34.2	55.7	35.1
Mizoram	30.8	29.3	15.9	16	22	27.9	25	26.9	28.9	29.5
Nagaland	34.2	35	15.6	19	22.6	25.6	26.1	48.6	40.8	34
Orissa	33.2	34.9	27.3	23.1	17.9	16	15.7	42.8	39.7	13.2
Punjab	62.6	62	54.6	57.2	58.4	57.9	60.9	61.8	62.4	62.5
Rajasthan	66.2	65.2	56.3	59.9	63.5	65.9	65.5	64.2	66.3	64.7
Sikkim	27.7	26.9	12.7	12.7	12.3	12.9	3	27.2	26.5	26.9
Tamil Nadu	41.4	41.5	37.6	37.6	37.1	38.5	39.4	43.1	41.9	40.3
Telengana	43.3	43.3	40.3	39.1	38.7	43.4	43.9	43.2	43.7	42
Tripura	55.3	55	31.4	38.1	41.7	43.2	47.9	55.2	54.9	54.9
Uttar Pradesh	86.1	85.3	72.7	72.8	74.4	76.2	76.3	86.8	84.8	84.5
Uttarakhand	62	61.9	47.3	49.8	59	66.3	65.3	57.4	70.4	59.7
West Bengal	65.5	64.5	61	60.2	62	62.9	63.9	63.9	62.9	64.8

Table S4.H: Estimated mean subgroup-weighted ambient $PM_{2.5}$ exposure (PWC, in μgm^{-3}) across the population subgroups in rural regions of the states in NFHS-4 (2015-16). We performed the 1st sensitivity analysis of assigning 2 km buffers for both urban and rural clusters, respectively.

State	Male	Female	Richest	Richer	Middle class	Poorer	Poorest	General	OBC	SC+ST
Andhra Pradesh	45.9	46.1	45.9	46.2	46.3	45.8	45.5	45.6	46.4	46.1
Arunachal Pradesh	41.2	40.5	39.2	36.5	36.7	38.3	39.1	48.7	46.1	38.6
Assam	59.1	59.4	56.8	57.7	57.4	58	58	60	57.6	58.4
Bihar	83.1	83.1	83	83.2	83.3	82.9	82.5	83.2	83.2	82.6
Chhattisgarh	45.7	45.2	45.8	45.6	45.9	44.9	43.5	43.8	46.4	44.3
Delhi	103.7	100.1	95.8	99.9	117.2	93.9	0	103.4	96.3	102.2
Goa	42.3	42.3	41.6	41.7	42.1	42.3	42.2	42.6	42.4	41.9
Gujarat	57.7	57.5	58	57.6	57.6	56.8	55.8	58.8	58.8	55.4
Haryana	81	80.5	80.4	79.6	78.7	79.6	80.5	80.6	80.9	80
Himachal Pradesh	47.4	46.6	46.9	46.2	46.2	45	43.9	46.4	48.4	46.7
Jammu & Kashmir	41	41	43.9	40.3	38.2	37.4	37.5	43.8	41.5	44.3
Jharkhand	58.9	58.1	55.3	56.4	56.5	57.2	58.1	57.5	59.3	57
Karnataka	33.4	33.4	34.2	33.1	33	33.4	33	33.1	33.7	33.1
Kerala	36.4	36.6	36.9	36.5	35.8	34.4	33.7	36.8	36.7	35.7
Madhya Pradesh	50.6	50.2	50.6	51	51	50.8	49	52.7	51.3	48.7
Maharashtra	45.7	45.5	45.5	45.3	45.3	45.6	46.1	44.1	46.7	46
Manipur	30.4	30.8	22.9	24.5	25.5	27.7	30.5	32.3	31.5	29.6
Meghalaya	43.8	43.7	46.4	42.8	40.8	45	45.2	49.4	39.9	42.3
Mizoram	36.4	37	33.2	34.3	34.4	38.1	44.7	39.5	46.9	36.3
Nagaland	35.7	36.6	36.7	35	33.7	32.5	34.5	46.8	50	36
Orissa	41.4	41.8	33.7	35.8	38.1	40.3	37.5	43.3	41.6	41.9
Punjab	61.8	61.8	60.8	60.9	61.4	62.1	60.6	62	61.2	61.9
Rajasthan	66.9	66.4	67.1	66.6	67.1	66.9	63.8	67.9	67.7	64.2
Sikkim	28.3	29	26.5	27.2	27.2	25.8	27.5	28.9	29.4	28.7
Tamil Nadu	41.9	42.3	42.3	41.7	41.4	42.5	42.2	40.7	42.2	42.4
Telengana	45.1	44.6	44.8	45.2	44.8	44	44.1	43.8	44.5	45
Tripura	55.1	55	43	52.3	54	54.3	53.8	55.2	55	55
Uttar Pradesh	80.6	80.5	83.9	81.9	81.1	79.7	78.7	81.2	80.6	79.9
Uttarakhand	60.3	57.7	58.5	58	55.6	56	56.7	49.6	67.3	62.9
West Bengal	66	66	64.9	65.4	65.1	66	66.3	65.2	66.3	65.9

Table S4.I: Estimated mean subgroup-weighted ambient $PM_{2.5}$ exposure (PWC, in μgm^{-3}) across the population subgroups in urban regions of the states in NFHS-5 (2019-21). We performed the 2nd sensitivity analysis of assigning 5 km buffers for both urban and rural clusters, respectively.

State	Male	Female	Richest	Richer	Middle class	Poorer	Poorest	General	OBC	SC+ST
Andhra Pradesh	34.8	35.3	31.6	33.5	34.7	34.4	35	34.7	35.1	36.6
Arunachal Pradesh	34.4	34.4	23.7	21.1	19.9	20.2	19.8	35.8	35	34.7
Assam	50.5	50.2	39.2	41	40.3	36.3	34.3	52.7	52.7	48.4
Bihar	60.8	63.1	52.5	59.9	60.9	59.4	64.4	56.4	64.6	65.8
Chhattisgarh	43.7	40.6	32.2	34.2	34.9	38.6	34.7	41.1	41	41
Delhi	46.4	52.4	12.6	14.5	12.6	20.7	27	50.4	55.7	57.5
Goa	26.5	26.6	22.1	21.6	21.6	19.8	15.3	26.1	27.7	26.3
Gujarat	43.4	45	32.3	33.8	33.7	34.9	40.1	43.9	44.4	46.2
Haryana	61.3	61.3	41.3	43.8	49.7	53.3	50	61.2	60.1	62.2
Himachal Pradesh	29	38.4	32.1	34.5	33.7	42	18.3	35.6	40.5	40.7
Jammu & Kashmir	31.8	36.2	23.1	20.3	24.4	28.2	31.8	33.6	41.6	43.6
Jharkhand	42.8	45.9	35	37.4	38.8	38.4	39.2	45.7	47.5	42.4
Karnataka	26.8	27.3	21.9	22.1	24.5	26.8	27.8	25.9	27.4	27.6
Kerala	27.3	27.3	25	26	27.3	27.2	20.9	25.8	27.6	28
Madhya Pradesh	44.8	45.1	36.4	38.6	41.2	44.4	42	45.4	45.9	43.4
Maharashtra	38.8	39.4	31.8	32.9	31.2	35.5	34.7	38.9	39.6	41
Manipur	35.8	25	11.2	13.7	11.6	14.9	19.1	26.8	28.1	26.3
Meghalaya	33.9	27.7	14.6	16.9	18	26.6	29.9	28.2	28.3	28.9
Mizoram	28.7	27	11.9	17.8	21.2	23.8	17.6	0	36.4	27.2
Nagaland	37.9	32.7	16	15.5	11.9	14.2	17.7	14.1	53.6	32.1
Orissa	45.9	43.6	37.8	39.3	40	42.5	45.1	42.1	44.9	44.6
Punjab	44.9	44.9	36.6	37.1	36.6	39.6	28.8	45.6	43.1	44.9
Rajasthan	57.5	55.7	48.8	51.8	52	54.4	60.9	54.3	56.8	56.5
Sikkim	1.8	15.4	4.7	5.5	7.4	10.9	8	24	13.1	12.1
Tamil Nadu	30.1	30	21.7	26.3	28.2	29.1	29.9	32.1	29.8	30.6
Telengana	38.1	35.8	25.7	28.3	28.2	30.2	31	34.3	36.3	36.9
Tripura	29.4	26.8	13.5	21.6	18	21.8	27.8	29.1	30.3	26.6
Uttar Pradesh	66.8	70	52.3	56.1	60.9	66.4	68.8	68.3	69.5	71.6
Uttarakhand	54.7	54.9	46.7	48.4	54.1	58.6	9.4	56.3	59.4	45.7
West Bengal	50.8	50.4	41.6	42	43.7	47.2	47.5	48.8	51	53.1

Table S4.J: Estimated mean subgroup-weighted ambient $PM_{2.5}$ exposure (PWC, in μgm^{-3}) across the population subgroups in rural regions of the states in NFHS-5 (2019-21). We performed the 2nd sensitivity analysis of assigning 5 km buffers for both urban and rural clusters, respectively.

State	Male	Female	Richest	Richer	Middle class	Poorer	Poorest	General	OBC	SC+ST
Andhra Pradesh	37.5	37.7	37.2	37.5	37.7	38	37.2	37.8	37.8	37.2
Arunachal Pradesh	40.8	39.1	31.8	33.8	35.1	35	37.4	48.3	45.2	38.1
Assam	51	52.9	52.6	52.6	51.2	50	48.9	53.7	53.4	53.2
Bihar	64.1	64.5	62.2	62.4	63.7	64	64	63.7	64.9	64.1
Chhattisgarh	43.6	43.3	43.7	43.6	42.9	43.3	40.9	44	44.5	42.3
Delhi	94	78.7	75.1	66.7	33.5	32.9	0	83	85.3	69.7
Goa	32.2	31.2	29.4	29.7	30.6	35.2	23.4	30.5	27.6	30
Gujarat	50.5	50.1	50.4	51	50.5	49.8	48.7	50.8	51	48.9
Haryana	67.2	67.3	66.1	67	65.8	66.5	65.9	68.4	67.5	65.9
Himachal Pradesh	43	42.5	41.5	41.8	42.9	42.8	44.4	43.5	41.7	41.7
Jammu & Kashmir	38.3	38.1	40	35.2	33.7	33.9	36.4	37.8	33.7	42.7
Jharkhand	50.6	49.9	47.7	48.8	47.8	48.3	50.1	51.4	50.1	49.6
Karnataka	30.1	30	29	29.2	29.4	30.6	31.4	29.5	30	30.2
Kerala	28.2	28.4	28.4	28.1	27.9	28	26.7	28.6	28.4	27.9
Madhya Pradesh	47.1	47.7	47.6	47.8	48	47.4	45.9	50	47.9	46.6
Maharashtra	41.5	41.6	41.4	41.5	41.3	41.5	42.1	40.2	42.3	42.2
Manipur	25.5	26.7	18.5	21.4	20.7	22.6	26	24.7	22.9	29.9
Meghalaya	37.8	37.8	28.3	33.2	37.2	35.5	35.6	38.8	41.9	37.6
Mizoram	34.5	33.7	30.2	31.5	31.5	32.4	33.8	0	31.7	34
Nagaland	32.2	32.6	24.5	24.4	29.6	29.4	28.9	32.3	25.8	32.4
Orissa	45.6	46.4	45.5	46.6	46.8	46.3	45.9	47.8	47.2	45.2
Punjab	51.3	51.3	49.7	50.3	50.1	51	49.3	51.8	51.5	50.9
Rajasthan	60.8	60.5	60	61	61.3	60.8	58.1	61.5	61.3	59.1
Sikkim	25.2	27	27.5	25	23.8	24.6	27	30.5	25.4	27.6
Tamil Nadu	32.1	32.8	32.2	32.3	32.5	32.7	32.5	31.2	32.8	32.6
Telengana	39.4	39.4	38.9	39.5	38.9	38.9	39.3	39.2	39.4	39.4
Tripura	45.7	43.8	40.1	37.2	36.6	37.6	41.1	41.8	44.2	45.3
Uttar Pradesh	74.8	74.7	74.6	74	73.8	73.6	73.1	75.4	74.5	74.7
Uttarakhand	57.1	55.1	57.1	55.6	50.1	48.7	50.1	49.3	62.8	56.4
West Bengal	54.5	55.2	56.2	55	54.7	55.2	55.3	54.3	55.6	55.4

Table S4.K: Estimated mean subgroup-weighted ambient $PM_{2.5}$ exposure (PWC, in μgm^{-3}) across the population subgroups in urban regions of the states in NFHS-4 (2015-16). We performed the 2nd sensitivity analysis of assigning 5 km buffers for both urban and rural clusters, respectively.

State	Male	Female	Richest	Richer	Middle class	Poorer	Poorest	General	OBC	SC+ST
Andhra Pradesh	45.2	45.4	43	41.8	41.6	44	43.3	46.1	45.1	45.3
Arunachal Pradesh	34.1	35.6	18.8	21	23.6	24.9	25.4	40	42	34.3
Assam	54.4	54.9	41.9	47.3	50.8	53.4	53.6	52.4	54.8	54.9
Bihar	71.7	69.7	49.9	61.3	66.2	66.8	72.8	67.4	69.3	74.6
Chhattisgarh	43.1	42.7	32.8	35.5	37.9	38.2	37.7	42.4	42.7	42.9
Delhi	65.1	61.8	29.4	34.3	35.2	28.5	0	62.9	73.3	58
Goa	29.1	29.2	28	22	22.7	24.8	42.2	24.8	30	32.1
Gujarat	53.1	50.7	40.3	43.6	48.8	51.3	43.9	49.8	53	50.2
Haryana	73.8	76.5	58.8	67.4	67	72.3	66.7	70.6	80.5	76
Himachal Pradesh	40.6	42.6	39.9	41	43.7	45.7	49.3	40	43.3	45.2
Jammu & Kashmir	32.6	32.7	29.6	19.9	21.8	28.8	35.7	42.6	47.2	45.3
Jharkhand	47.4	47.8	32.4	33.1	35.3	38.9	37.2	49.4	47.9	45.5
Karnataka	31.5	31.5	22.9	27.5	30.4	32.3	31.8	32.5	31.5	31.3
Kerala	31.7	32	30.4	30.3	29.8	29	21.6	34.1	31.1	32.9
Madhya Pradesh	48.3	47.6	34.6	37.1	40.8	43.7	45.2	47.6	47.9	47.7
Maharashtra	40.8	40	33.5	33.9	32.6	35.2	40.2	38.9	42.5	40.3
Manipur	18.1	19.4	5.4	6.2	8.9	9.5	12.8	15.3	16.4	24.4
Meghalaya	24.3	29.1	14.6	14.1	19.3	25.6	30.5	33.8	55.7	28.6
Mizoram	28.2	28.8	14.6	15.5	20.9	27.3	25.6	9.3	32.7	28.7
Nagaland	25.8	25.3	7.7	10.7	13.8	15.1	14.5	23.8	19	25.4
Orissa	21	19.4	13.6	7.2	3.7	4.7	14.9	9.7	23.1	5.2
Punjab	58.5	57.1	50.1	51.8	52.2	50.1	51.9	57.2	57.4	57.4
Rajasthan	61.3	62	49.9	54.2	59.5	60.2	58.2	60.8	62.9	61.4
Sikkim	22.9	22.1	8.2	8.2	9.6	10.2	3.1	22.4	20.2	23.6
Tamil Nadu	35.8	35.6	26	30.8	32.9	34.6	37.7	41	35.7	35.2
Telengana	35	35.1	27.9	25.9	32.7	37.2	43.1	31.9	37.3	31.6
Tripura	38.7	43.5	8.2	21.8	25.6	35	47.8	41.8	41	40.8
Uttar Pradesh	72.4	71.3	51.2	56.3	61.4	66.3	69.3	67.6	73	74.1
Uttarakhand	53.2	49	34.7	37.9	43.4	52.1	59	47.8	49.2	52
West Bengal	57.2	55.1	46.4	45.4	51.7	56.6	63.3	54.3	52.7	58.9

Table S4.L: Estimated mean subgroup-weighted ambient $PM_{2.5}$ exposure (PWC, in μgm^{-3}) across the population subgroups in rural regions of the states in NFHS-4 (2015-16). We performed the 2nd sensitivity analysis of assigning 5 km buffers for both urban and rural clusters, respectively.

State	Male	Female	Richest	Richer	Middle class	Poorer	Poorest	General	OBC	SC+ST
Andhra Pradesh	45.9	46.1	45.9	46.2	46.3	45.8	45.5	45.6	46.3	46.1
Arunachal Pradesh	38.3	37.4	34.2	31.8	33.3	35.3	36	43.4	40.1	36
Assam	55	55.7	54.2	52.6	53.5	53.8	53.9	54.5	54.4	55.6
Bihar	80.6	80.2	78.7	79.5	79.1	79.4	79.6	79.8	80.5	79.6
Chhattisgarh	45.3	45	45.7	45.4	45.6	44.7	43.3	42.5	46.2	44.2
Delhi	104.9	102	97.3	102.2	120.3	95.9	0	104.9	98.3	104.4
Goa	35.5	35.1	33.3	34.8	37.6	38.9	32.7	34.7	35	38.8
Gujarat	57.6	57.1	58	57.1	57	56.6	55.8	58.3	58.4	55
Haryana	77.3	77.7	76.9	77	75.4	75.3	71.9	77.8	77.5	77.8
Himachal Pradesh	45.1	44.7	44	43.7	44	44.1	43	44.5	47.3	44.3
Jammu & Kashmir	37.5	37.4	39.1	35.3	33.9	34.6	35.2	41.8	38.3	42.5
Jharkhand	55.9	55.2	50.8	50.8	51.5	53.2	55.5	54	56.1	54.5
Karnataka	33.2	33.3	34	32.8	32.9	33.3	33	33.1	33.6	33
Kerala	35.2	34.7	35.3	34.9	33.2	32.9	33.3	35.3	35.1	33.1
Madhya Pradesh	50.3	50	50.2	50.5	50.7	50.6	48.8	52.3	50.9	48.6
Maharashtra	45.6	45.4	45.3	45.2	45.3	45.5	46	44	46.7	45.9
Manipur	23	23.1	13.8	13.5	15.8	18.6	25.2	20.2	16.3	27.2
Meghalaya	42.5	41	41.4	38.7	37.9	41.8	40.6	48.1	39.2	40.1
Mizoram	34.9	35.1	32.7	32.8	32.6	35.1	42.6	36.3	39.4	34.8
Nagaland	32.9	32.2	27.8	27.8	29.3	29.1	30.6	31.9	32.8	32.2
Orissa	29.7	35	22.1	27.9	30.9	34.8	33.8	2.6	30.4	39.3
Punjab	61	60.3	59.3	59.4	59.9	60.9	59	60.5	59.7	60.6
Rajasthan	66.8	66.3	67	66.4	66.9	66.7	63.3	67.8	67.6	63.9
Sikkim	19	19.7	14.7	16.6	16.7	15.4	15.5	18.5	18.5	20.9
Tamil Nadu	40.9	41.5	40.6	40.4	40.4	41.7	41.5	39.1	41.3	41.6
Telengana	45	44.3	44.7	44.8	44.5	43.8	43.2	43.8	44.3	44.7
Tripura	52.6	53.6	42.1	48.8	51.7	51.3	52.1	52.9	53.2	53.3
Uttar Pradesh	78.3	77.8	78.7	78.5	77.8	77.1	76.6	78.9	77.8	77.4
Uttarakhand	55.8	52.8	52.9	51.6	49.6	51.3	52.2	47.1	59.8	57.3
West Bengal	65.4	65.4	64.6	63.9	64.3	65.6	65.9	64.9	65.2	65.7

Table S5.A: Estimated Z_{score} (95% confidence intervals, CIs) across the population subgroups in urban regions of the states in NFHS-5 (2019-21). We performed the 1st sensitivity analysis of assigning 2 km buffers for both urban and rural clusters, respectively.

State	Male-Female	Richest - Richer	Richest – Middle class	Richest - Poorer	Richest - Poorest	General - OBC	General – (SC+ST)
Andhra Pradesh	0.0012	0.0079	-0.0056	0.018	-0.0011	0.0045	0.0022
Arunachal Pradesh	0.0037	-0.0127	0.0055	-0.0327	-0.1164	-0.0236	0.1109
Assam	-0.0017	-0.0682	-0.0636	0.0152	0.0197	0.0106	0.0364
Bihar	0.0001	-0.125	-0.1625	-0.1625	-0.15	-0.0063	0.0219
Chhattisgarh	0.0017	-0.0156	-0.0122	-0.04	0.0122	0.0089	0.0244
Delhi	-0.0054	0.1206	0.1412	-0.0706	0.1029	0.1176	0.1118
Goa	0.0002	-0.0457	-0.0514	0.1057	-0.1114	0.0029	-0.0057
Gujarat	-0.0075	-0.0435	-0.0522	-0.0739	-0.1674	-0.0174	-0.0478
Haryana	0.0049	-0.1294	-0.2029	-0.3412	-0.15	-0.0853	0
Himachal Pradesh	-0.0047	0.0443	0.0586	-0.0014	0.3271	0.0186	0.0257
Jammu & Kashmir	-0.0005	0.0873	0.0273	-0.0182	-0.1145	0.0273	-0.0473
Jharkhand	0.0015	0	-0.0027	-0.0081	-0.0257	0.0054	0.0149
Karnataka	0.0005	0.0116	-0.0217	-0.0275	-0.0478	-0.013	-0.0087
Kerala	-0.0003	-0.0083	0	0.0063	-0.0104	0	0.0021
Madhya Pradesh	0.0006	-0.0246	-0.0507	-0.0783	-0.0304	0.0014	0.0391
Maharashtra	-0.0019	0.0216	0.0622	-0.0054	0.0257	0.0095	0.0162
Manipur	0.0046	-0.0598	-0.0659	-0.122	-0.15	0.011	-0.0134
Meghalaya	0.0051	-0.0605	-0.0842	-0.3421	-0.3947	0	-0.2605
Mizoram	0.0042	-0.0902	-0.1118	-0.1294	0.0137	-0.6961	-0.6039
Nagaland	0.0098	0.0519	0.1426	0.0667	0.013	-0.3648	-0.113
Orissa	0.002	0.0559	0.0324	-0.0029	-0.0529	-0.0618	-0.0471
Punjab	0.0004	-0.0323	-0.0435	-0.0339	-0.0226	-0.0016	-0.0065
Rajasthan	0.0023	-0.0403	-0.0455	-0.061	-0.1	-0.0052	-0.0052
Sikkim	0.0025	0.017	0.0151	0.1	0.3245	-0.0604	-0.0396
Tamil Nadu	-0.0016	-0.0692	-0.075	-0.075	-0.1	0.025	0.0385
Telengana	-0.0005	-0.0188	-0.0275	-0.0463	-0.0675	-0.0213	-0.0275
Tripura	-0.0087	0.0607	0.0494	-0.0011	0.0202	0.0011	0.0034
Uttar Pradesh	-0.0006	-0.0136	-0.0339	-0.0678	-0.0254	-0.0034	-0.0136
Uttarakhand	-0.0025	-0.0565	-0.1536	-0.1884	-0.0507	-0.0667	0.0087
West Bengal	0	0.0087	0.0196	-0.0348	-0.0652	0.0152	0.0022

Table S5.B: Estimated Z_{score} (95% confidence intervals, CIs) across the population subgroups in rural regions of the states in NFHS-5 (2019-21). We performed the 1st sensitivity analysis of assigning 2 km buffers for both urban and rural clusters, respectively.

State	Male-Female	Richest - Richer	Richest – Middle class	Richest - Poorer	Richest - Poorest	General - OBC	General – (SC+ST)
Andhra Pradesh	-0.0009	-0.0059	-0.0118	-0.0196	-0.002	-0.002	0.0098
Arunachal Pradesh	0.0013	0.0138	0.008	0	-0.0299	0.0264	0.0989
Assam	0.0003	0.0111	0.0167	0.0319	0.0444	0.0167	0.0292
Bihar	-0.0004	0.013	0.0091	0.0143	0.0195	0	0.0065
Chhattisgarh	0.0005	0	0.0107	0.0067	0.0373	-0.004	0.0267
Delhi	-0.0042	-0.0898	-0.1102	0.0186	1.578	0.0695	0.039
Goa	-0.0009	0	-0.0024	-0.0071	-0.0119	-0.0048	0.0119
Gujarat	-0.0002	-0.0047	0.0016	0.0094	0.0219	-0.0047	0.0219
Haryana	0	0	0.0053	0	-0.0079	0.0053	0.0368
Himachal Pradesh	-0.0002	-0.0016	0	0.0016	-0.0016	-0.0145	0.0081
Jammu & Kashmir	-0.0002	0.0694	0.0944	0.1083	0.0917	0.0361	-0.0361
Jharkhand	0.0006	-0.0023	-0.0034	-0.0125	-0.0182	0.0182	0.0341
Karnataka	0	0.0012	0	-0.0131	-0.0226	-0.0012	-0.0048
Kerala	-0.0003	0	0.0067	0.03	0.0933	-0.0067	0.02
Madhya Pradesh	-0.0001	-0.0018	0.0018	0.0123	0.0404	0.0316	0.0579
Maharashtra	0	-0.0027	0	-0.0027	-0.012	-0.0293	-0.028
Manipur	0.0005	-0.1406	-0.1594	-0.2281	-0.2594	-0.0156	0.0438
Meghalaya	-0.0014	-0.186	-0.2628	-0.2047	-0.2093	-0.2326	0.1419
Mizoram	0.0011	0.0044	-0.0133	-0.0444	-0.1556	-0.9556	-0.8111
Nagaland	-0.0014	0.0314	0.0657	0.1114	0.1214	-0.2714	-0.0671
Orissa	-0.0002	-0.0113	-0.0081	-0.0032	0.0065	0.0097	0.0339
Punjab	0	-0.014	-0.008	-0.01	-0.014	0.016	0.008
Rajasthan	0.0005	-0.025	-0.035	-0.0225	0.045	0.01	0.065
Sikkim	0.0009	0.007	-0.0105	0	-0.007	0.0053	0.0088
Tamil Nadu	-0.0004	0.0032	0.0032	-0.0032	0.0194	0.0065	0.0097
Telengana	-0.0003	-0.0058	-0.0012	0.0012	-0.0012	0.0012	0
Tripura	-0.0006	0.1154	0.0904	0.0788	0.0442	-0.0038	-0.0058
Uttar Pradesh	0	0.024	0.034	0.046	0.06	0.016	0.026
Uttarakhand	0.0026	0.0684	0.1789	0.1895	0.1711	-0.3474	-0.2
West Bengal	-0.0004	-0.0026	-0.0013	-0.0077	-0.009	-0.0154	-0.0179

Table S5.C: Estimated Z_{score} (95% confidence intervals, CIs) across the population subgroups in urban regions of the states in NFHS-4 (2015-16). We performed the 1st sensitivity analysis of assigning 2 km buffers for both urban and rural clusters, respectively.

State	Male-Female	Richest - Richer	Richest – Middle class	Richest - Poorer	Richest - Poorest	General - OBC	General – (SC+ST)
Andhra Pradesh	0.0011	0.0205	0.0386	0.0023	0.0545	0.0159	0.0205
Arunachal Pradesh	0.0031	-0.0014	-0.0057	-0.0243	-0.0529	-0.0143	0.0943
Assam	0.0009	-0.0452	-0.056	-0.0905	-0.0893	-0.0024	0.0048
Bihar	-0.004	-0.0719	-0.1109	-0.1281	-0.1344	-0.0016	0.0031
Chhattisgarh	0.0008	-0.024	-0.054	-0.038	-0.024	0.004	0.014
Delhi	0.0004	-0.2433	-0.3933	-0.15	0.9167	0.0233	-0.03
Goa	0	0	0.0231	0.0756	-0.0372	-0.0038	-0.0013
Gujarat	-0.0008	-0.0094	-0.0625	-0.025	0.2094	-0.0656	-0.0188
Haryana	-0.0016	-0.0765	-0.0741	-0.1235	0.0296	-0.037	-0.0086
Himachal Pradesh	0.0041	-0.0029	0.0103	0.0074	-0.0618	-0.1029	0.0103
Jammu & Kashmir	-0.0008	0.1694	0.0776	0.0429	-0.0224	-0.0449	0.0184
Jharkhand	0	-0.052	-0.044	-0.1213	-0.1053	-0.0107	0.0213
Karnataka	-0.0003	-0.0133	-0.0644	-0.0978	-0.08	0.0111	0.02
Kerala	-0.0005	0.0097	0.0032	0.0097	-0.0113	0.0081	0.0032
Madhya Pradesh	0	-0.0265	-0.0794	-0.1103	-0.1118	-0.0103	0.0074
Maharashtra	-0.0005	-0.0046	-0.0046	-0.0262	-0.0369	-0.0123	-0.0046
Manipur	0.0033	-0.0415	-0.1208	-0.1585	-0.2038	0	-0.0377
Meghalaya	0.0002	-0.0269	-0.0846	-0.2654	-0.3596	-0.4135	-0.0173
Mizoram	0.0026	-0.0018	-0.1109	-0.2182	-0.1655	-0.0364	-0.0473
Nagaland	-0.0011	-0.0576	-0.1186	-0.1695	-0.178	0.1322	0.2475
Orissa	-0.0022	0.0824	0.1843	0.2216	0.2275	0.0608	0.5804
Punjab	0.001	-0.0867	-0.1267	-0.11	-0.21	-0.02	-0.0233
Rajasthan	0.0013	-0.0554	-0.1108	-0.1477	-0.1415	-0.0323	-0.0077
Sikkim	0.0016	0	0.0066	-0.0033	0.159	0.0115	0.0049
Tamil Nadu	-0.0002	0	0.0139	-0.025	-0.05	0.0333	0.0778
Telengana	0	0.0207	0.0276	-0.0534	-0.0621	-0.0086	0.0207
Tripura	0.0007	-0.1241	-0.1907	-0.2185	-0.3056	0.0056	0.0056
Uttar Pradesh	0.0011	-0.0012	-0.0202	-0.0417	-0.0429	0.0238	0.0274
Uttarakhand	0.0002	-0.041	-0.1918	-0.3115	-0.2951	-0.2131	-0.0377
West Bengal	0.0018	0.0129	-0.0161	-0.0306	-0.0468	0.0161	-0.0145

Table S5.D: Estimated Z_{score} (95% confidence intervals, CIs) across the population subgroups in rural regions of the states in NFHS-4 (2015-16). We performed the 1st sensitivity analysis of assigning 2 km buffers for both urban and rural clusters, respectively.

State	Male-Female	Richest - Richer	Richest – Middle class	Richest - Poorer	Richest - Poorest	General - OBC	General – (SC+ST)
Andhra Pradesh	-0.0003	-0.0043	-0.0058	0.0014	0.0058	-0.0116	-0.0072
Arunachal Pradesh	0.0018	0.0303	0.0281	0.0101	0.0011	0.0292	0.1135
Assam	-0.0008	-0.0205	-0.0136	-0.0273	-0.0273	0.0545	0.0364
Bihar	0	-0.0026	-0.0038	0.0013	0.0064	0	0.0077
Chhattisgarh	0.0013	0.003	-0.0015	0.0136	0.0348	-0.0394	-0.0076
Delhi	0.0051	-0.0707	-0.369	0.0328	1.6517	0.1224	0.0207
Goa	0	-0.0015	-0.0075	-0.0104	-0.009	0.003	0.0104
Gujarat	0.0003	0.0085	0.0085	0.0255	0.0468	0	0.0723
Haryana	0.001	0.0195	0.0415	0.0195	-0.0024	-0.0073	0.0146
Himachal Pradesh	0.0011	0.0079	0.0079	0.0213	0.0337	-0.0225	-0.0034
Jammu & Kashmir	0	0.1	0.1583	0.1806	0.1778	0.0639	-0.0139
Jharkhand	0.0009	-0.0153	-0.0167	-0.0264	-0.0389	-0.025	0.0069
Karnataka	0	0.0234	0.0255	0.017	0.0255	-0.0128	0
Kerala	-0.0004	0.0077	0.0212	0.0481	0.0615	0.0019	0.0212
Madhya Pradesh	0.0004	-0.0073	-0.0073	-0.0036	0.0291	0.0255	0.0727
Maharashtra	0.0003	0.0042	0.0042	-0.0021	-0.0125	-0.0542	-0.0396
Manipur	-0.0009	-0.0184	-0.0299	-0.0552	-0.0874	0.0092	0.031
Meghalaya	0.0002	0.1	0.1556	0.0389	0.0333	0.2639	0.1972
Mizoram	-0.0009	-0.0297	-0.0324	-0.1324	-0.3108	-0.2	0.0865
Nagaland	-0.0012	0.0258	0.0455	0.0636	0.0333	-0.0485	0.1636
Orissa	-0.0006	-0.0239	-0.05	-0.075	-0.0432	0.0193	0.0159
Punjab	0	-0.0016	-0.0098	-0.0213	0.0033	0.0131	0.0016
Rajasthan	0.0007	0.0077	0	0.0031	0.0508	0.0031	0.0569
Sikkim	-0.0009	-0.0113	-0.0113	0.0113	-0.0161	-0.0081	0.0032
Tamil Nadu	-0.0011	0.02	0.03	-0.0067	0.0033	-0.05	-0.0567
Telengana	0.0008	-0.0059	0	0.0118	0.0103	-0.0103	-0.0176
Tripura	0.0002	-0.155	-0.1833	-0.1883	-0.18	0.0033	0.0033
Uttar Pradesh	0.0001	0.0235	0.0329	0.0494	0.0612	0.0071	0.0153
Uttarakhand	0.0032	0.0076	0.0439	0.0379	0.0273	-0.2682	-0.2015
West Bengal	0	-0.0069	-0.0028	-0.0153	-0.0194	-0.0153	-0.0097

Table S5.E: Estimated Z_{score} (95% confidence intervals, CIs) across the population subgroups in urban regions of the states in NFHS-5 (2019-21). We performed the 2nd sensitivity analysis of assigning 5 km buffers for both urban and rural clusters, respectively.

State	Male-Female	Richest - Richer	Richest – Middle class	Richest - Poorer	Richest - Poorest	General - OBC	General – (SC+ST)
Andhra Pradesh	0.0012	0.0189	-0.0135	0.0432	-0.0027	0.0108	0.0054
Arunachal Pradesh	0.0037	-0.0088	0.0037	-0.0225	-0.08	-0.0163	0.0762
Assam	-0.0018	-0.1023	-0.0955	0.0227	0.0295	0.0159	0.0545
Bihar	0.0002	-0.08	-0.104	-0.104	-0.096	-0.004	0.014
Chhattisgarh	0.0011	-0.0341	-0.0268	-0.0878	0.0268	0.0195	0.0537
Delhi	-0.0061	0.0745	0.0873	-0.0436	0.0636	0.0727	0.0691
Goa	0.0002	-0.0193	-0.0217	0.0446	-0.047	0.0012	-0.0024
Gujarat	-0.0033	-0.0625	-0.075	-0.1062	-0.2406	-0.025	-0.0687
Haryana	0.0042	-0.1	-0.1568	-0.2636	-0.1159	-0.0659	0
Himachal Pradesh	-0.0041	0.0449	0.0594	-0.0014	0.3319	0.0188	0.0261
Jammu & Kashmir	-0.0004	0.0686	0.0214	-0.0143	-0.09	0.0214	-0.0371
Jharkhand	0.001	0	-0.0065	-0.0194	-0.0613	0.0129	0.0355
Karnataka	0.0008	0.0094	-0.0176	-0.0224	-0.0388	-0.0106	-0.0071
Kerala	-0.0003	-0.0098	0	0.0073	-0.0122	0	0.0024
Madhya Pradesh	0.0015	-0.0258	-0.053	-0.0818	-0.0318	0.0015	0.0409
Maharashtra	-0.0014	0.0205	0.059	-0.0051	0.0244	0.009	0.0154
Manipur	0.0066	-0.1361	-0.15	-0.2778	-0.3417	0.025	-0.0306
Meghalaya	0.0034	-0.0277	-0.0386	-0.1566	-0.1807	0	-0.1193
Mizoram	0.0045	-0.0836	-0.1036	-0.12	0.0127	-0.6455	-0.56
Nagaland	0.0045	0.0483	0.1328	0.0621	0.0121	-0.3397	-0.1052
Orissa	0.0015	0.0322	0.0186	-0.0017	-0.0305	-0.0356	-0.0271
Punjab	0.0002	-0.0667	-0.09	-0.07	-0.0467	-0.0033	-0.0133
Rajasthan	0.0036	-0.0838	-0.0946	-0.127	-0.2081	-0.0108	-0.0108
Sikkim	0.0038	0.0167	0.0148	0.0981	0.3185	-0.0593	-0.0389
Tamil Nadu	-0.0011	-0.0783	-0.0848	-0.0848	-0.113	0.0283	0.0435
Telengana	-0.0004	-0.0246	-0.0361	-0.0607	-0.0885	-0.0279	-0.0361
Tripura	-0.0041	0.135	0.11	-0.0025	0.045	0.0025	0.0075
Uttar Pradesh	-0.0014	-0.0131	-0.0328	-0.0656	-0.0246	-0.0033	-0.0131
Uttarakhand	-0.0014	-0.0929	-0.2524	-0.3095	-0.0833	-0.1095	0.0143
West Bengal	0	0.0082	0.0184	-0.0327	-0.0612	0.0143	0.002

Table S5.F: Estimated Z_{score} (95% confidence intervals, CIs) across the population subgroups in rural regions of the states in NFHS-5 (2019-21). We performed the 2nd sensitivity analysis of assigning 5 km buffers for both urban and rural clusters, respectively.

State	Male-Female	Richest - Richer	Richest – Middle class	Richest - Poorer	Richest - Poorest	General - OBC	General – (SC+ST)
Andhra Pradesh	-0.0006	-0.0045	-0.0091	-0.0152	-0.0015	-0.0015	0.0076
Arunachal Pradesh	0.0011	0.0167	0.0097	0	-0.0361	0.0319	0.1194
Assam	0.0002	0.0151	0.0226	0.0434	0.0604	0.0226	0.0396
Bihar	-0.0003	0.0182	0.0127	0.02	0.0273	0	0.0091
Chhattisgarh	0.0009	0	0.0145	0.0091	0.0509	-0.0055	0.0364
Delhi	-0.0065	-0.0671	-0.0823	0.0139	1.1785	0.0519	0.0291
Goa	-0.0008	0	-0.0013	-0.0039	-0.0065	-0.0026	0.0065
Gujarat	-0.0002	-0.0094	0.0031	0.0188	0.0437	-0.0094	0.0437
Haryana	0	0	0.0029	0	-0.0044	0.0029	0.0206
Himachal Pradesh	-0.0002	-0.0029	0	0.0029	-0.0029	-0.0257	0.0143
Jammu & Kashmir	-0.0001	0.0316	0.043	0.0494	0.0418	0.0165	-0.0165
Jharkhand	0.0003	-0.0049	-0.0073	-0.0268	-0.039	0.039	0.0732
Karnataka	0	0.0013	0	-0.0139	-0.0241	-0.0013	-0.0051
Kerala	-0.0001	0	0.0024	0.0107	0.0333	-0.0024	0.0071
Madhya Pradesh	-0.0002	-0.0031	0.0031	0.0219	0.0719	0.0562	0.1031
Maharashtra	0	-0.0022	0	-0.0022	-0.01	-0.0244	-0.0233
Manipur	0.0007	-0.0672	-0.0761	-0.109	-0.1239	-0.0075	0.0209
Meghalaya	-0.0016	-0.2222	-0.3139	-0.2444	-0.25	-0.2778	0.1694
Mizoram	0.0012	0.0054	-0.0162	-0.0541	-0.1892	-1.1622	-0.9865
Nagaland	-0.0019	0.0256	0.0535	0.0907	0.0988	-0.2209	-0.0547
Orissa	-0.0006	-0.0132	-0.0094	-0.0038	0.0075	0.0113	0.0396
Punjab	0	-0.0086	-0.0049	-0.0062	-0.0086	0.0099	0.0049
Rajasthan	0.0007	-0.0164	-0.023	-0.0148	0.0295	0.0066	0.0426
Sikkim	0.0007	0.0048	-0.0072	0	-0.0048	0.0036	0.006
Tamil Nadu	-0.0004	0.002	0.002	-0.002	0.0122	0.0041	0.0061
Telengana	-0.0002	-0.0081	-0.0016	0.0016	-0.0016	0.0016	0
Tripura	-0.0005	0.08	0.0627	0.0547	0.0307	-0.0027	-0.004
Uttar Pradesh	0	0.0293	0.0415	0.0561	0.0732	0.0195	0.0317
Uttarakhand	0.0021	0.0426	0.1115	0.118	0.1066	-0.2164	-0.1246
West Bengal	-0.0003	-0.0036	-0.0018	-0.0109	-0.0127	-0.0218	-0.0255

Table S5.G: Estimated Z_{score} (95% confidence intervals, CIs) across the population subgroups in urban regions of the states in NFHS-4 (2015-16). We performed the 2nd sensitivity analysis of assigning 5 km buffers for both urban and rural clusters, respectively.

State	Male-Female	Richest - Richer	Richest – Middle class	Richest - Poorer	Richest - Poorest	General - OBC	General – (SC+ST)
Andhra Pradesh	0.0012	0.0265	0.05	0.0029	0.0706	0.0206	0.0265
Arunachal Pradesh	0.0035	-0.0014	-0.0056	-0.0239	-0.0521	-0.0141	0.093
Assam	0.0007	-0.0585	-0.0723	-0.1169	-0.1154	-0.0031	0.0062
Bihar	-0.0074	-0.1211	-0.1868	-0.2158	-0.2263	-0.0026	0.0053
Chhattisgarh	0.0015	-0.0218	-0.0491	-0.0345	-0.0218	0.0036	0.0127
Delhi	0.0005	-0.1304	-0.2107	-0.0804	0.4911	0.0125	-0.0161
Goa	0	0	0.0228	0.0747	-0.0367	-0.0038	-0.0013
Gujarat	-0.0005	-0.01	-0.0667	-0.0267	0.2233	-0.07	-0.02
Haryana	-0.0011	-0.1292	-0.125	-0.2083	0.05	-0.0625	-0.0146
Himachal Pradesh	0.0036	-0.005	0.0175	0.0125	-0.105	-0.175	0.0175
Jammu & Kashmir	-0.0006	0.1339	0.0613	0.0339	-0.0177	-0.0355	0.0145
Jharkhand	0	-0.0684	-0.0579	-0.1596	-0.1386	-0.014	0.0281
Karnataka	-0.0001	-0.0182	-0.0879	-0.1333	-0.1091	0.0152	0.0273
Kerala	-0.0012	0.0171	0.0057	0.0171	-0.02	0.0143	0.0057
Madhya Pradesh	0	-0.03	-0.09	-0.125	-0.1267	-0.0117	0.0083
Maharashtra	-0.0002	-0.0063	-0.0063	-0.0354	-0.05	-0.0167	-0.0062
Manipur	0.0067	-0.0449	-0.1306	-0.1714	-0.2204	0	-0.0408
Meghalaya	0.0001	-0.0259	-0.0815	-0.2556	-0.3463	-0.3981	-0.0167
Mizoram	0.0023	-0.0032	-0.1968	-0.3871	-0.2935	-0.0645	-0.0839
Nagaland	-0.0013	-0.0944	-0.1944	-0.2778	-0.2917	0.2167	0.4056
Orissa	-0.0028	0.1355	0.3032	0.3645	0.3742	0.1	0.9548
Punjab	0.0019	-0.0565	-0.0826	-0.0717	-0.137	-0.013	-0.0152
Rajasthan	0.0014	-0.0947	-0.1895	-0.2526	-0.2421	-0.0553	-0.0132
Sikkim	0.0024	0	0.0121	-0.0061	0.2939	0.0212	0.0091
Tamil Nadu	-0.0002	0	0.0065	-0.0117	-0.0234	0.0156	0.0364
Telengana	0	0.0267	0.0356	-0.0689	-0.08	-0.0111	0.0267
Tripura	0.0005	-0.1136	-0.1746	-0.2	-0.2797	0.0051	0.0051
Uttar Pradesh	0.001	-0.0014	-0.023	-0.0473	-0.0486	0.027	0.0311
Uttarakhand	0.0001	-0.0472	-0.2208	-0.3585	-0.3396	-0.2453	-0.0434
West Bengal	0.0019	0.0095	-0.0119	-0.0226	-0.0345	0.0119	-0.0107

Table S5.H: Estimated Z_{score} (95% confidence intervals, CIs) across the population subgroups in rural regions of the states in NFHS-4 (2015-16). We performed the 2nd sensitivity analysis of assigning 5 km buffers for both urban and rural clusters, respectively.

State	Male-Female	Richest - Richer	Richest – Middle class	Richest - Poorer	Richest - Poorest	General - OBC	General – (SC+ST)
Andhra Pradesh	-0.0003	-0.0035	-0.0047	0.0012	0.0047	-0.0094	-0.0059
Arunachal Pradesh	0.0012	0.0529	0.049	0.0176	0.002	0.051	0.198
Assam	-0.0006	-0.02	-0.0133	-0.0267	-0.0267	0.0533	0.0356
Bihar	0	-0.0045	-0.0068	0.0023	0.0114	0	0.0136
Chhattisgarh	0.0007	0.0062	-0.0031	0.0281	0.0719	-0.0813	-0.0156
Delhi	0.0101	-0.0532	-0.2779	0.0247	1.2442	0.0922	0.0156
Goa	0	-0.0013	-0.0063	-0.0089	-0.0076	0.0025	0.0089
Gujarat	0.0003	0.0069	0.0069	0.0207	0.0379	0	0.0586
Haryana	0.0009	0.0096	0.0205	0.0096	-0.0012	-0.0036	0.0072
Himachal Pradesh	0.0011	0.014	0.014	0.038	0.06	-0.04	-0.006
Jammu & Kashmir	0	0.059	0.0934	0.1066	0.1049	0.0377	-0.0082
Jharkhand	0.0017	-0.0162	-0.0176	-0.0279	-0.0412	-0.0265	0.0074
Karnataka	0	0.0229	0.025	0.0167	0.025	-0.0125	0
Kerala	-0.0003	0.0056	0.0155	0.0352	0.0451	0.0014	0.0155
Madhya Pradesh	0.0011	-0.0093	-0.0093	-0.0047	0.0372	0.0326	0.093
Maharashtra	0.0002	0.0024	0.0024	-0.0012	-0.0071	-0.0306	-0.0224
Manipur	-0.0008	-0.041	-0.0667	-0.1231	-0.1949	0.0205	0.0692
Meghalaya	0.0001	0.1161	0.1806	0.0452	0.0387	0.3065	0.229
Mizoram	-0.0009	-0.0175	-0.019	-0.0778	-0.1825	-0.1175	0.0508
Nagaland	-0.0013	0.0246	0.0435	0.0609	0.0319	-0.0464	0.1565
Orissa	-0.0005	-0.0677	-0.1419	-0.2129	-0.1226	0.0548	0.0452
Punjab	0	-0.0014	-0.0086	-0.0186	0.0029	0.0114	0.0014
Rajasthan	0.0007	0.0125	0	0.005	0.0825	0.005	0.0925
Sikkim	-0.0009	-0.0108	-0.0108	0.0108	-0.0154	-0.0077	0.0031
Tamil Nadu	-0.001	0.0162	0.0243	-0.0054	0.0027	-0.0405	-0.0459
Telengana	0.0009	-0.0075	0	0.0151	0.0132	-0.0132	-0.0226
Tripura	0.0002	-0.1162	-0.1375	-0.1412	-0.135	0.0025	0.0025
Uttar Pradesh	0.0002	0.027	0.0378	0.0568	0.0703	0.0081	0.0176
Uttarakhand	0.0082	0.0075	0.0433	0.0373	0.0269	-0.2642	-0.1985
West Bengal	0	-0.0111	-0.0044	-0.0244	-0.0311	-0.0244	-0.0156

Table S6: Estimated results from the concordance test among the NFHS and GHSL datasets. *'1'* and *'0'* denote the rural-rural and urban-urban coincidence, respectively, and *'N'* is the total number of clusters across the NFHS rounds. a[1,1] and d[0,0] count the number of geo-locations with matching rural-rural and urban-urban coincidence across these two datasets, respectively; while b[1,0] and c[0,1] denote the mismatches.

	**a[1,1]**	**b[1,0]**	**c[0,1]**	**d[0,0]**	**Concordance coefficient [(a+d)/N]**
**NFHS-5 and GHSL-2020** (N=30197)	15074	5368	3177	6578	0.717
**NFHS-4 and GHSL-2015** (N = 28527)	14012	5187	2959	6369	0.714

Table S7: Ambient $PM_{2.5}$ concentration statistics (mean national estimates across 10th, 33rd, 50th, 67th, and 90th percentiles) in the urban and rural regions across both the NFHS rounds. We used the 33rd and 66th percentile estimates to segregate ambient $PM_{2.5}$ distribution into high, moderate, and low concentration categories.

	**NFHS-5 (2019-21)**		**NFHS-4 (2015-16)**	
	**Urban**	**Rural**	**Urban**	**Rural**
Mean	51.5 µgm^{-3} (28.6-86)	53 µgm^{-3} (30-80.6)	57.7 µgm^{-3} (31.2-93.2)	58 µgm^{-3} (30.9-86.8)
10th percentile	34 µgm^{-3}	30.6 µgm^{-3}	35 µgm^{-3}	35.6 µgm^{-3}
33rd percentile	40.8 µgm^{-3}	37.6 µgm^{-3}	42.8 µgm^{-3}	43.3 µgm^{-3}
50th percentile	49.6 µgm^{-3}	47.9 µgm^{-3}	52.6 µgm^{-3}	55.5 µgm^{-3}
67th percentile	67.3 µgm^{-3}	60.8 µgm^{-3}	72.3 µgm^{-3}	76 µgm^{-3}
90th percentile	76.9 µgm^{-3}	78.4 µgm^{-3}	85.5 µgm^{-3}	82.7 µgm^{-3}

Table S8: Assessment of ambient $PM_{2.5}$ exposure across the population subgroups stratified by gender, wealth-index, and caste subgroups of NFHS-4. "*CV*" signifies the *Coefficient of Variation*. In the "*No. of districts*" columns, the *numbers* denote the number of districts where the corresponding subgroups breathed in higher ambient $PM_{2.5}$ concentration (p<0.1) as compared to their reference subgroup counterparts. The numeric values in parentheses indicate the number of districts (out of 707) where the standardized Z_{score} estimates have *p*-values within the considered significance threshold (*p*<0.1).

**Urban region**								
**Population subgroups**		**Ambient $PM_{2.5}$ estimates**		**Sub-population fraction (in-%)**	**Population fraction (%) of subgroup**			**No. of districts**
		PWC (CV)	Z_{score} (95% CI)		>66th percentile (>72.3 μgm^{-3})	34th-66th percentile (42.8-72.3 μgm^{-3})	<34th percentile (<42.8 μgm^{-3})	
Gender	Male	56.3 (0.339)	Reference	51.3	52.1	50.9	48.8	Reference
	Female	57.1 (0.337)	-0.0171 (-0.014 – -0.0202)	48.7	47.9	49.1	51.2	293 (637)
Wealth	Richest	57.5 (0.315)	Reference	20.3	20.25	19.59	19.24	Reference
	Richer	55.2 (0.317)	0.0073 (0.0062 – 0.0084)	28.2	28.85	26.59	29.11	131 (435)
	Middle class	56.9 (0.317)	0.0279 (0.0218-0.034)	24.4	24.66	27.64	25.38	139 (435)
	Poorer	59.4 (0.315)	-0.0002 (-0.0001 – -0.0003)	17.1	16.94	17.35	17.54	139 (435)
	Poorest	64.1 (0.316)	-0.0625 (-0.0462 – -0.0788)	9.9	9.3	8.83	8.73	137 (435)
Caste	General	59.8 (0.323)	Reference	41	38.26	31.19	28.6	Reference
	OBC	56.2 (0.323)	0.0014 (-0.0011 – -0.0017)	35.2	36.58	46.11	45.58	263 (575)
	SC+ST	56.2 (0.322)	0.0276 (0.0224-0.0328)	23.8	25.16	22.7	25.83	284 (575)
**Rural region**								
**Population subgroups**		**Ambient $PM_{2.5}$ estimates**		**Sub-population fraction (in-%)**	**Population fraction (%) of subgroup**			**No. of districts**
		PWC (CV)	Z_{score} (95% CI)		>66th percentile (>76 μgm^{-3})	34th-66th percentile (43.3-76 μgm^{-3})	<34th percentile (<43.3 μgm^{-3})	
Gender	Male	59.2 (0.318)	Reference	51.2	52	51.5	52.2	Reference
	Female	60.7 (0.316)	-0.0255 (-0.0194 – -0.0316)	48.8	48	48.5	47.8	304 (626)

Wealth	Richest	58.9 (0.314)	Reference	12.2	12.23	11.34	11.46	Reference
	Richer	56.1 (0.311)	0.0842 (0.0707-0.0977)	19.5	20.58	21.08	24.18	255 (578)
	Middle class	57.1 (0.31)	0.0977 (0.0684-0.127)	26	26.78	30.43	30.6	251 (578)
	Poorer	61.2 (0.313)	-0.0114 (-0.0095 – -0.0133)	25.4	24.85	25.44	22.7	261 (578)
	Poorest	66.2 (0.316)	-0.0945 (-0.0765 – -0.1134)	16.9	15.56	11.7	11.05	261 (578)
Caste	General	61.2 (0.305)	Reference	43.3	26.47	28.41	24.37	Reference
	OBC	62.3 (0.309)	-0.1067 (-0.0864 – -0.127)	25.2	39.07	41.38	44.86	277 (581)
	SC+ST	58.1 (0.306)	0.0137 (0.0101 – 0.0173)	31.5	34.46	30.21	30.77	267 (581)

Table S9: Estimated absolute disparities (in μgm^{-3}, difference between 99th and 1st percentiles of the distributions) across the urban and rural regions of the states in both NFHS rounds. For this sensitivity assessment, we used the NFHS-defined urban and rural clusters.

	NFHS-5 (2019-21)		NFHS-4 (2015-16)	
**State**	**Urban**	**Rural**	**Urban**	**Rural**
Andhra Pradesh	16.6	17.3	20.2	22.1
Arunachal Pradesh	30.2	28.2	30.4	32.7
Assam	30.8	29.6	37.7	35.3
Bihar	21.6	19.5	24.7	29.7
Chhattisgarh	19.5	18.9	20.9	21.4
Delhi	25.8	25.9	33	24.4
Goa	4.9	2.5	4.1	3.3
Gujarat	13.1	15.5	21.3	26.4
Haryana	44	32.5	57.2	42.9
Himachal Pradesh	17.2	15.3	26.4	27.1
Jammu & Kashmir	16	15.2	26.4	25.4
Jharkhand	28.2	28.1	42.3	43.4
Karnataka	15.1	16.1	17.4	17.3
Kerala	6.9	8.7	11.5	15.2
Madhya Pradesh	41.6	40.7	45.7	45
Maharashtra	19.2	17.7	17.1	16.9
Manipur	25.4	22.1	30.3	25.6
Meghalaya	37	35.7	38.5	43.6
Mizoram	26.9	23.8	26.8	26.4
Nagaland	27.1	26.8	30.8	29.8
Orissa	19.1	19	26.1	27.4
Punjab	13.6	14.7	15.6	16.2
Rajasthan	36.4	38.2	40.6	40.8
Sikkim	5.8	9.1	15	15.9
Tamil Nadu	12.7	12.9	27.1	26.1
Telengana	13.5	12.8	15.2	14.9
Tripura	3.2	4	5.3	7.6
Union Territories	12.4	13.6	11.5	14.2
Uttar Pradesh	59.1	47.3	56	57.3
Uttarakhand	37.3	38	50.4	49.9
West Bengal	33	30.4	52.2	45.3

Table S10.A: Estimated effect size (mean Z_{score}) across the population subgroups in urban regions of NFHS-5 (2019-21).

	Male - Female	Richest - Richer	Richest - Middle class	Richest - Poorer	Richest - Poorest	General - OBC	General - (SC+ST)
Andhra Pradesh	0.053	0.001	-0.002	0.003	-0.001	0.024	-0.005
Arunachal Pradesh	0.000	0.001	0.001	0.001	0.002	0.013	-0.004
Assam	-0.012	-0.019	0.003	-0.009	0.004	0.010	-0.021
Bihar	0.023	0.054	0.038	0.035	0.044	0.017	0.002
Chhattisgarh	-0.014	0.001	0.024	-0.009	0.014	0.038	0.006
Delhi	0.047	0.000	0.000	0.000	0.000	-0.017	0.073
Goa	0.000	0.000	0.000	0.000	0.000	0.266	-0.056
Gujarat	0.023	-0.006	-0.019	-0.033	-0.002	-0.019	-0.021
Haryana	0.050	0.204	0.052	0.023	-0.005	0.009	0.008
Himachal Pradesh	0.035	0.000	0.000	0.000	0.000	-0.023	-0.024
Jammu & Kashmir	-0.033	0.000	0.000	0.000	0.000	0.000	0.000
Jharkhand	0.026	0.002	-0.006	-0.003	-0.003	0.001	0.001
Karnataka	-0.023	0.032	0.023	0.041	0.023	0.001	0.011
Kerala	-0.057	0.000	0.000	0.000	0.000	0.001	0.084
Madhya Pradesh	0.034	-0.009	0.004	0.025	0.049	-0.042	0.001
Maharashtra	-0.018	-0.045	-0.070	-0.037	-0.061	0.007	0.019
Manipur	0.087	-0.011	0.008	-0.014	-0.011	0.042	-0.040
Meghalaya	-0.054	0.034	-0.023	-0.106	-0.059	0.000	0.000
Mizoram	0.215	0.000	0.000	0.000	0.000	---	---
Nagaland	0.050	-0.009	0.014	0.052	0.083	---	---
Orissa	-0.015	0.010	-0.006	0.002	-0.020	0.016	-0.011
Punjab	-0.163	0.022	0.096	0.070	0.034	-0.060	-0.054
Rajasthan	0.007	0.011	0.028	0.035	0.001	0.016	0.005
Sikkim	0.307	0.000	0.000	0.000	0.000	-0.290	0.424
Tamil Nadu	0.028	-0.005	0.006	0.030	-0.028	-0.010	-0.007
Telengana	-0.042	-0.003	0.002	-0.004	0.000	-0.002	0.008
Tripura	-0.119	0.076	0.099	0.052	0.019	0.137	0.089
Uttar Pradesh	0.000	0.003	-0.001	-0.002	-0.001	0.011	0.001
Uttarakhand	-0.078	-0.001	-0.005	-0.007	-0.003	-0.061	-0.045
West Bengal	-0.084	-0.015	0.012	0.004	0.008	0.003	-0.053

Table S10.B: Estimated effect size (mean Z_{score}) across the population subgroups in rural regions of NFHS-5 (2019-21).

	Male-Female	Richest-Richer	Richest-Middle class	Richest-Poorer	Richest-Poorest	General-OBC	General-(SC+ST)
Andhra Pradesh	-0.020	-0.035	-0.040	-0.071	-0.051	-0.008	-0.003
Arunachal Pradesh	0.022	0.010	0.004	0.005	0.003	0.053	0.086
Assam	0.015	0.061	0.035	0.056	0.077	0.024	-0.027
Bihar	0.000	0.005	0.008	0.015	0.033	-0.003	0.043
Chhattisgarh	0.016	0.032	0.030	0.005	0.039	0.013	0.040
Delhi	-0.535	0.000	0.000	0.000	0.000	0.131	0.022
Goa	-0.494	0.000	0.000	0.000	0.000	-0.042	0.653
Gujarat	-0.025	-0.057	-0.021	-0.032	-0.017	-0.006	-0.016
Haryana	-0.018	0.031	0.079	0.096	0.052	0.001	-0.012
Himachal Pradesh	-0.031	0.075	0.106	0.105	0.011	-0.049	-0.009
Jammu & Kashmir	-0.181	0.020	0.001	0.012	-0.004	0.000	0.000
Jharkhand	0.031	-0.043	0.008	0.019	0.017	-0.012	0.016
Karnataka	0.000	0.001	-0.018	-0.030	-0.008	-0.008	0.007
Kerala	0.000	-0.031	0.016	0.027	0.021	0.018	0.072
Madhya Pradesh	-0.015	0.015	0.025	0.008	0.017	0.001	-0.006
Maharashtra	-0.021	0.006	0.007	0.031	-0.001	-0.011	-0.006
Manipur	0.195	0.028	0.007	-0.025	0.003	0.045	-0.094
Meghalaya	-0.039	0.002	-0.013	0.048	0.007	0.000	0.000
Mizoram	0.070	0.005	0.021	0.026	-0.012	---	---
Nagaland	0.000	0.007	0.086	0.136	0.099	---	---
Orissa	-0.033	-0.024	0.014	-0.004	0.006	-0.008	0.019
Punjab	0.000	-0.005	-0.018	-0.048	-0.082	0.023	0.039
Rajasthan	0.007	-0.008	-0.022	-0.018	-0.036	-0.005	0.016
Sikkim	0.227	0.064	-0.039	-0.107	-0.112	-0.102	0.057
Tamil Nadu	-0.029	-0.016	0.019	0.018	0.043	0.000	-0.036
Telengana	0.000	-0.012	0.007	0.002	0.017	-0.015	-0.006
Tripura	0.115	0.021	-0.081	0.009	0.061	-0.068	-0.059
Uttar Pradesh	0.010	-0.006	0.001	0.007	-0.010	-0.010	0.008
Uttarakhand	0.000	0.068	0.112	0.139	0.099	-0.037	-0.034
West Bengal	-0.015	0.007	0.077	0.102	0.141	-0.010	-0.063

Table S10.C: Estimated effect size (mean Z_{score}) across the population subgroups in urban regions of NFHS-4 (2015-16).

	Male-Female	Richest-Richer	Richest-Middle class	Richest-Poorer	Richest-Poorest	General-OBC	General-(SC+ST)
Andhra Pradesh	0.183	0.000	0.000	0.105	0.000	0.023	-0.014
Arunachal Pradesh	-0.102	0.166	0.125	-0.048	0.187	-0.078	0.253
Assam	0.000	-0.001	0.000	0.139	-0.004	0.009	0.003
Bihar	0.014	-0.018	-0.017	0.096	-0.009	-0.002	0.005
Chhattisgarh	0.070	-0.023	-0.045	0.007	-0.032	0.020	0.026
Delhi	-0.234	0.000	0.000	0.000	0.000	0.073	0.077
Goa	0.000	0.000	0.000	-0.107	0.000	-0.029	-0.055
Gujarat	0.227	-0.008	-0.047	0.005	-0.047	0.003	0.013
Haryana	0.022	-0.021	-0.001	0.136	-0.030	0.013	0.001
Himachal Pradesh	0.000	0.000	0.000	0.000	0.000	-0.117	0.077
Jammu & Kashmir	0.065	0.007	0.052	-0.025	0.022	-0.028	-0.049
Jharkhand	-0.035	0.008	0.011	0.026	-0.005	-0.009	-0.016
Karnataka	0.115	0.044	0.046	0.009	0.056	-0.008	0.005
Kerala	0.000	0.000	0.000	0.048	0.000	-0.009	-0.002
Madhya Pradesh	-0.029	0.014	-0.007	0.102	0.013	-0.011	0.000
Maharashtra	0.010	0.006	-0.019	0.019	-0.009	-0.011	-0.021
Manipur	0.050	0.011	-0.017	-0.004	0.113	-0.045	-0.230
Meghalaya	-0.049	-0.036	-0.010	0.005	0.046	0.000	0.000
Mizoram	-0.008	0.001	0.019	0.008	-0.032	---	---
Nagaland	-0.099	0.018	0.036	-0.032	-0.006	0.000	0.000
Orissa	0.000	-0.007	-0.004	0.000	-0.014	0.004	-0.008
Punjab	-0.040	0.000	0.000	-0.071	0.000	0.028	0.016
Rajasthan	0.125	-0.007	0.004	-0.030	-0.014	-0.012	0.057
Sikkim	1.977	0.000	0.000	0.000	0.000	0.170	0.179
Tamil Nadu	-0.021	0.001	0.028	0.027	0.026	0.017	0.038
Telengana	-0.024	-0.002	-0.001	0.012	-0.003	0.008	-0.024
Tripura	0.000	-0.039	-0.068	0.018	-0.080	-0.014	0.087
Uttar Pradesh	0.087	-0.010	-0.016	-0.273	0.018	0.008	0.012
Uttarakhand	1.488	0.017	-0.014	-0.002	0.035	-0.055	-0.025
West Bengal	0.000	0.021	0.014	0.002	0.008	0.057	0.020

Table S10.D: Estimated effect size (mean Z_{score}) across the population subgroups in rural regions of NFHS-4 (2015-16).

	Male-Female	Richest-Richer	Richest-Middle class	Richest-Poorer	Richest-Poorest	General-OBC	General-(SC+ST)
Andhra Pradesh	-0.014	-0.013	0.004	0.030	0.044	0.022	-0.025
Arunachal Pradesh	0.025	0.051	0.028	0.037	-0.021	0.045	0.052
Assam	-0.012	0.006	-0.004	-0.027	-0.024	0.019	-0.036
Bihar	0.000	-0.033	-0.023	-0.040	-0.022	-0.005	0.025
Chhattisgarh	0.014	0.015	0.030	0.047	0.010	-0.012	-0.014
Delhi	0.123	0.000	0.000	0.000	0.000	0.000	0.000
Goa	0.000	0.134	-0.101	-0.166	0.035	-0.148	0.281
Gujarat	0.026	0.019	0.017	0.020	0.000	0.014	0.020
Haryana	0.025	0.021	0.029	0.022	-0.003	-0.010	0.002
Himachal Pradesh	0.064	0.000	0.000	0.000	0.000	-0.049	-0.032
Jammu & Kashmir	0.000	-0.004	0.074	0.058	0.069	0.004	-0.009
Jharkhand	0.025	-0.003	0.017	0.053	0.061	0.035	0.037
Karnataka	0.000	-0.031	0.017	0.018	0.024	-0.019	-0.039
Kerala	-0.022	-0.002	-0.015	-0.011	0.027	-0.007	0.074
Madhya Pradesh	0.007	-0.009	-0.008	-0.014	-0.006	-0.043	-0.006
Maharashtra	0.021	0.014	0.005	0.019	0.004	-0.020	0.004
Manipur	-0.131	0.147	0.047	0.032	-0.023	0.000	0.000
Meghalaya	-0.021	-0.055	0.070	0.031	-0.004	0.000	0.000
Mizoram	-0.046	0.059	0.141	0.069	-0.064	---	---
Nagaland	-0.012	0.039	0.083	0.040	0.061	-0.009	0.082
Orissa	0.011	0.001	0.002	-0.004	-0.007	-0.009	0.023
Punjab	-0.029	0.002	-0.052	0.008	-0.033	0.059	0.018
Rajasthan	0.014	0.014	0.013	-0.004	0.024	-0.005	-0.007
Sikkim	-0.160	0.000	0.000	0.000	0.000	-0.109	0.046
Tamil Nadu	-0.028	0.004	0.024	0.014	-0.007	-0.011	-0.007
Telengana	0.071	0.009	0.024	-0.004	0.039	-0.085	-0.049
Tripura	0.000	-0.020	-0.081	-0.009	0.001	-0.022	-0.065
Uttar Pradesh	-0.009	0.007	0.013	0.010	0.011	-0.006	-0.004
Uttarakhand	0.010	0.044	0.078	0.057	0.049	-0.032	-0.036
West Bengal	-0.069	-0.002	0.029	0.020	0.035	0.049	0.011

Reference

Katoch, V., Kumar, A., Imam, F., Sarkar, D., Knibbs, L. D., Liu, Y., ... & Dey, S. (2023). Addressing biases in ambient PM2.5 exposure and associated health burden estimates by filling satellite AOD retrieval gaps over India. *Environmental Science & Technology*, *57*(48), 19190-19201.
